# Understanding spatial patterns of soils for sustainable agriculture in northern Ethiopia’s tropical mountains

**DOI:** 10.1371/journal.pone.0224041

**Published:** 2019-10-22

**Authors:** Jan Nyssen, Sander Tielens, Tesfamichael Gebreyohannes, Tigist Araya, Kassa Teka, Johan Van de Wauw, Karen Degeyndt, Katrien Descheemaeker, Kassa Amare, Mitiku Haile, Amanuel Zenebe, Neil Munro, Kristine Walraevens, Kindeya Gebrehiwot, Jean Poesen, Amaury Frankl, Alemtsehay Tsegay, Jozef Deckers

**Affiliations:** 1 Department of Geography, Ghent University, Gent, Belgium; 2 Department of Earth and Environmental Sciences, KU Leuven, Leuven, Belgium; 3 Department of Earth Sciences, Mekelle University, Mekelle, Ethiopia; 4 Department of Land Resources Management and Environmental Protection, Mekelle University, Mekelle, Ethiopia; 5 Department of Plant Production Systems, Wageningen University, Wageningen, The Netherlands; 6 Institute of Climate and Society, Mekelle University, Mekelle, Ethiopia; 7 Department of Geology, Ghent University, Gent, Belgium; 8 Research Foundation—Flanders (FWO), Brussels, Belgium; 9 Department of Dryland Crop and Horticultural Sciences, Mekelle University, Mekelle, Ethiopia; Georgia Southern University, UNITED STATES

## Abstract

Knowledge of the geographical distribution of soils is indispensable for policy and decision makers to achieve the goal of increasing agricultural production and reduce poverty, particularly in the Global South. A study was conducted to better understand the soilscapes of the Giba catchment (900–3300 m a.s.l.; 5133 km^2^) in northern Ethiopia, so as to sustain soil use and management. To characterise the chemical and physical properties of the different benchmark soils and to classify them in line with the World Reference Base of Soil Resources, 141 soil profile pits and 1381 soil augerings at representative sites were analysed. The dominant soil units identified are Leptosol and bare rock (19% coverage), Vertic Cambisol (14%), Regosol and Cambisol (10%), Skeletic/Leptic Cambisol and Regosol (9%), Rendzic Leptosol (7%), Calcaric/Calcic Vertisol (6%), Chromic Luvisol (6%) and Chromic/Pellic Vertisol (5%). Together these eight soil units cover almost 75% of the catchment. Topography and parent material are the major influencing factors that explain the soil distribution. Besides these two factors, land cover that is strongly impacted by human activities, may not be overlooked. Our soil suitability study shows that currently, after thousands of years of agricultural land use, a new dynamic equilibrium has come into existence in the soilscape, in which ca. 40% of the catchment is very suitable, and 25% is moderately suitable for agricultural production. In view of such large suitable areas, the Giba catchment has a good agricultural potential if soil erosion rates can be controlled, soil fertility (particularly nitrogen) increased, available water optimally used, and henceforth crop yields increased.

## Introduction

Good land management is characterised by making optimal use of the natural resources including soils in a sustainable way. In the Giba catchment (5133 km^2^), north Ethiopia, poverty has been largely attributed to insufficient crop production [1, 2]. Soil degradation in this area became important when humans started deforestation almost 5000 years ago [3, 4]. The resulting reduced soil protection by vegetation cover, combined with steep slopes and erosive rainfall led to excessive soil erosion [5, 6]. Nutrients and organic matter (OM) were lost and soil depth was reduced. Expanding the agricultural areas into less suitable lands to sustain crop production would further increase soil erosion rates. Yet, the high population density allows a more intensive use of the available agricultural land. In recent decades, many soil and water conservation measures (SWCM) have been carried out to reduce soil erosion rates and to increase crop production. Ex-situ SWCM include the construction of stone bunds, infiltration trenches, check dams in gullies, micro-dams and ponds as well as a range of biological measures (e.g. exclosures), while in-situ soil management measures are being promoted (e.g. intercropping, bed and furrows, zero tillage, zero grazing) [3, 7–11]. Despite these SWCM, soil erosion still is an important problem, which results in low crop yields and biomass production. In view of all this, the Tigray region, where the Giba catchment is located, has chronically suffered of food insufficiencies. To curb such situations, soil maps have proven to be powerful tools for understanding soil processes [12], for the establishment of technical infrastructure[13], and in support of land management policies [14, 15].

Hunting Technical Services [16] prepared landform and land suitability maps of an area largely encompassing Giba catchment at a scale of 1:250,000, and further maps of landforms and soils at 1:50,000 for areas around Mekelle, Hawzien, Sinkata and Wuqro. Soil mapping and land evaluation have been carried out in several parts of Giba basin by student teams of IAO Firenze (led by Luca Ongaro and Valeria Alessandro) [17–21]. Other available baseline soil information for the study area comprises mainly small-scale maps based on FAO [22] at 1:1,000,000; derived maps include the web-based e-SOTER soil information system [23] and the corresponding sheets in the Soil Atlas of Africa [24, 25]. The development of a national soil model at scale of 1:500,000 has been attempted [26], as well as soil nutrient mapping through the EthioSIS and AfroSIS programmes [27], resulting among others in detailed maps of soil fertility status and recommended fertiliser blends with a resolution of 250 m for the whole Tigray region [28]. Whereas the latter have a deliberate focus on chemical fertilizer requirements [29], all other mentioned maps are very generalized, allowing a regional comprehension of the soil distribution, but not at all a full understanding of the spatial patterns of the soils in a given area.

Therefore, the main objective of this study is to contribute to sustainable land management in the Giba catchment through a better understanding of the soil types and their characteristics, which is a prerequisite for analysing soil suitability for sustainable agricultural production.

A good knowledge of the geographical distribution of the soils and their chemical and physical properties is thus indispensable for policy and decision makers to improve land management and hence reduce poverty and increase the welfare of the population in north Ethiopia.

Characterisation of benchmark soils of the catchment, both in the field and in the laboratory, was combined with all available information into a comprehensive spatially explicit database of soils in Giba catchment, at a scale of 1:250,000. This allows a fundamental insight into the soil properties and the soilscapes of the Giba catchment which is needed to enhance sustainable natural resource use and management.

## Study area

**Fig 1:**
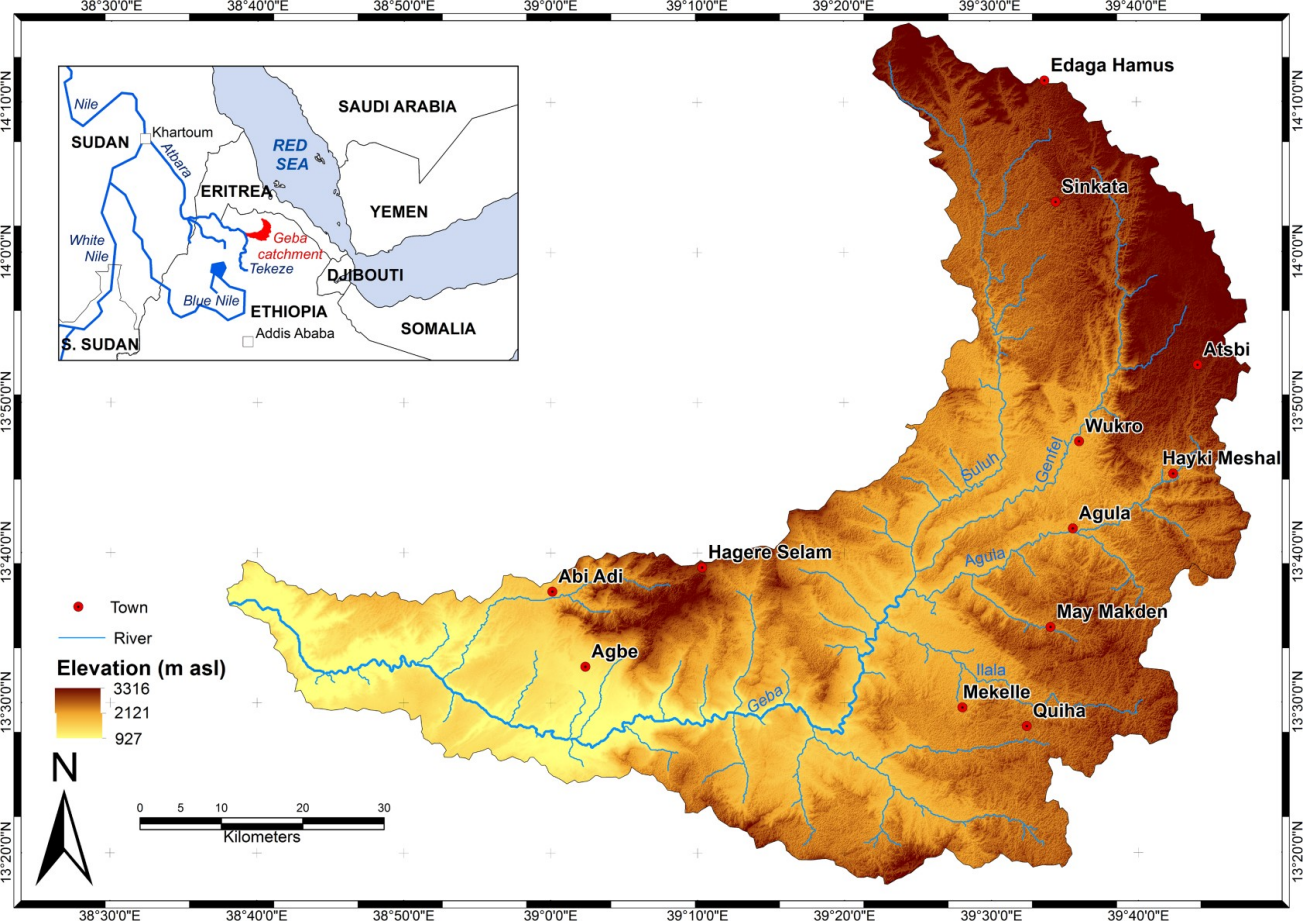
Map of Giba catchment.

The Giba catchment is in Tigray region (North Ethiopia), between 13°18’N and 14°15’N and 38°38’E and 39°48’E, and comprises the region’s capital city Mekelle (Fig 1). The Giba River is a tributary of the Tekezze River, which becomes Atbara River in Sudan where it flows into the Nile. The altitude in the catchment varies from slightly over 900 m a.s.l. in the western part to more than 3300 m a.s.l. in the north. The mean elevation of the catchment is 2144 m with a standard deviation of 361 m indicating that the topography is very rugged. Because of high elevations, the climate is more temperate than would be expected at this latitude [30].

**Fig 2:**
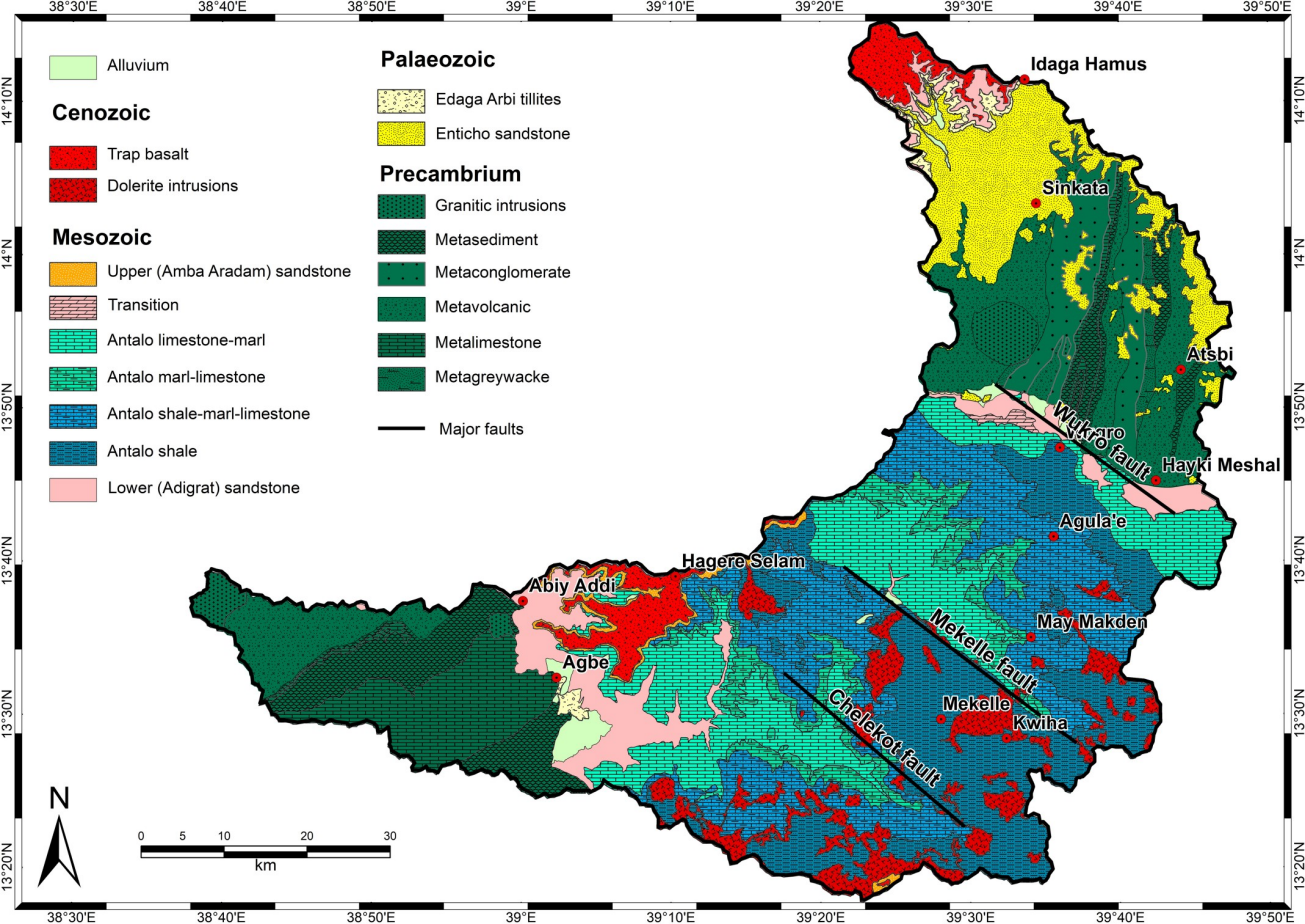
Geological map of the Giba catchment [34].

The geology of the catchment consists of a Precambrian basement complex, Palaeozoic (fluvio-)glacial rocks, Mesozoic sedimentary rocks, Tertiary volcanics and Quaternary deposits [31] (Fig 2). The landscape is characterised by a strongly incised river network. Major faults are responsible for steep cliffs. The alternation of different lithologies resulted in a stepped geomorphology due to selective erosion [32, 33].

According to the Köppen climate classification, the area is hot semi-arid (BSh) [35]. Annual rainfall depth varies between less than 600 mm and 1000 mm, but no significant relationship with altitude exists [30, 36]. Most rains fall during the main rainy season, which typically extends from June to September. Mean annual maximum air temperature ranges from 21 to 31°C and mean annual minimum temperature from 3 to 16°C [37]. Monthly potential evapotranspiration (PET) exceeds monthly rainfall except during the rainy season due to reduced sunshine hours and increased rainfall. However, monthly rainfall is only slightly higher than PET in the northernmost part during the rainy season while elsewhere in the catchment rainfall greatly exceeds PET. Hence, the length of the growing period (LGP), defined as the period during which the precipitation is at least half of the PET [38], is shortest in the northernmost part.

Recent land use maps [34, 39, 40] show that 42–50% of the Giba catchment is covered by cropland, followed by shrubland (37%). Forests are rare (2.3%), however the eastern part of the catchment holds Des’a Forest, one of the few forests in north Ethiopia, on the edge of the Rift Valley escarpment.

**Fig 3:**
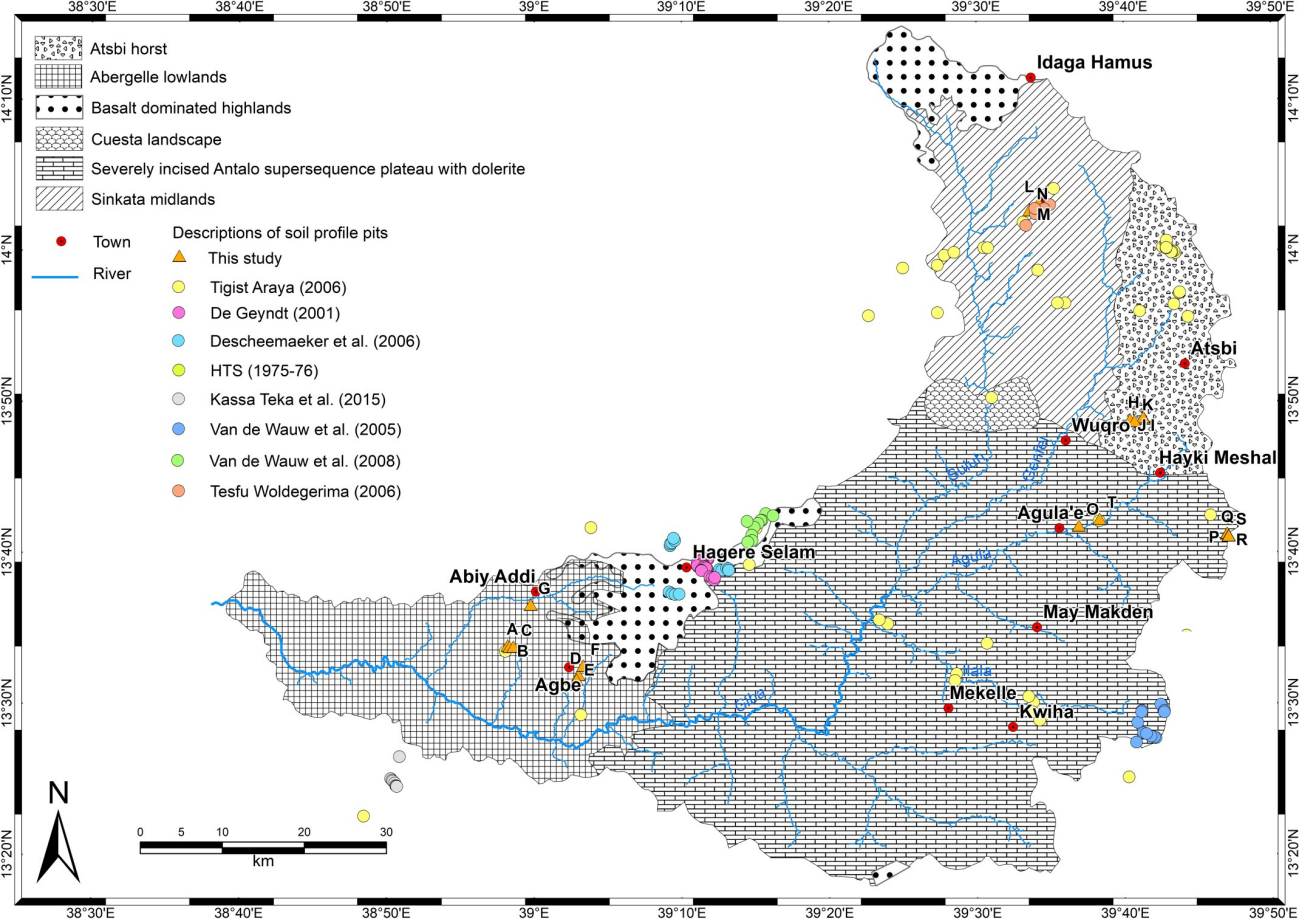
Major geomorphic regions of the Giba catchment, with location of soil profile pits including those characterised in this article (A-T).

Based on lithology, geological structure, geomorphology, elevation and climate, the Giba catchment can be subdivided into 6 major geomorphic regions: the Atsbi horst, the Abergelle lowlands, the basalt-dominated highlands, the cuesta landscape, the severely incised Antalo Supersequence plateau with dolerite, and the Sinkata midlands (Fig 3). According to FAO [22] and the Soil Atlas of Africa [24], the catchment would be dominated by Lithic and Eutric Leptosol, Vertic and Chromic Cambisol, and Haplic Lixisol.

## Part I. Soil characteristics

### Materials and methods

Field work comprised ten field campaigns in the Giba catchment (1974, 1975, 2001–2011), and the analysis of 141 profile pits (Table 1). For every field campaign, district authorities issued permits, and especially, the landholders gave permission for digging profile pits. Generally they dug out the pit themselves manually and were very keen to discuss the observations with the researchers. For pits on communal lands we obtained permission from the village chairperson, who assigned nearby residents for pit excavation. All labour was paid in cash, at a rate approximately 50% above salaries paid locally for similar works. None of the data collection involved endangered or protected animal or plant species. Disturbed and undisturbed samples were taken from the different horizons for further chemical and physical analysis. Undisturbed samples were taken with Kopecki rings, 100 cm^3^ steel cylinders, 5 cm across, driven in the soil using a ring holder. Here we focus on 20 representative profile pits from which 46 soil horizons were sampled.

**Table 1:** Overview of field surveys for soil data collection.

Study area	Geomorphic region	Profile pits	Augerings	Notes	Source
Ruba Feleg	Atsbi Horst	14	175	13 additional augerings for model validation	[41]
May Zegzeg	Basalt-dominated highlands; Antalo plateau	21	206		[42, 43]
Adawro, Khunale, May Bi'ati	Basalt-dominated highlands; Antalo plateau	15	225		[44]
Aqushala	Abergelle Lowlands	9	288	16 additional pits for model validation	[45]
Chichat	Antalo plateau	13			[46]
May Leiba	Basalt-dominated highlands	11	230	18 additional augerings for model validation	[47]
Tsinkaniet	Sinkata Midlands	6	191	20 additional augerings for model validation	[48]
Rift Valley shoulder	all	12			[49]
Rift Valley shoulder	all	20			[16, 30]
Giba catchment	all	20	66		[50]
Total		141	1381		

#### Physical analysis

The texture of all horizons was investigated in the field using the finger method [51]. Further, formal physical analysis was done both on the disturbed and undisturbed samples. The analysis of the undisturbed samples was done in Mekelle University (Ethiopia), the analysis of the disturbed samples was done in KU Leuven (Belgium) soil laboratories.

**Texture** of the soil horizons was analysed by 2 different methods: with a wet sieving followed by a decantation and tentatively with a laser diffraction particle size analyser (LDPSA). Upper texture class boundaries were conventionally set at 2 μm (clay), 50 μm (silt) and 2 mm (sand).

In preparation for the analysis by LDPSA, the samples were dried, roots and small plant remnants removed, mortared and sieved at 2 mm. With the aid of a sample splitter a very small amount (<1 g) was separated into a test tube. Distilled water was added and this mixture was boiled for at least 15 minutes to bring all particles in suspension. This mixture was analysed with the LDPSA. During the analysis, ultrasonic sound or ultrasound bath was used to break the particles apart. The laser beam reflected on the particles and this reflection is dependent on the size of the particles. Large particles (sand) provide a reflection at an angle that is smaller than small particles (clay). Each detector detects a different particle size ranging from < 0.04 μm to 1822 μm. After the analysis, the mass percentage of each particle size was known [52].

Standard wet sieving and decantation was also conducted on eight samples to compare the results with the LDPSA method. About 20 g of the dried and sieved (at 2 mm) soil was weighted. 50 ml of peptiser (sodium oxalate dispersing agent) was added and the mixture was diluted with distilled water to about 150 ml. Then the sample was boiled for at least 10 minutes to destroy the aggregates. After cooling, the mixture was sieved at 50 μm and the filtrate was used for the decantation. The particles that stayed behind on the sieve were collected in a pre-weighed cup and dried in an oven of 105°C for 24 hours, after which the cup was weighed again. The filtrate was put in a decantation column and diluted to 1 litre. The column was shaken well for a few seconds. Immediately after that, 50 ml was tapped and added into a pre-weighed noggin. Three more times 50 ml was tapped at 4’30”, 13’30” and 2h30’, which corresponds to the fractions smaller than 32 μm, 16 μm and 2 μm. Fifteen seconds before each tapping the tube was cleaned by tapping till the next line on the column. The noggins were also put in an oven of 105°C for 24 hours and weighed afterwards.

In the LDPSA measurement, unexpectedly, we found for almost all the samples a silt loam texture [50]. However, the eight samples that were also analysed with the conventional decantation method were clearly finer textured, as clay percentages measured with the decantation method were much higher than those obtained through LDPSA. To further evaluate the difference in preparation method between LDPSA and the conventional method, the same eight samples that were analysed with the conventional method were also analysed again with the LDPSA but with the same preparation as in the conventional method. This includes the addition of peptiser and distilled water followed by boiling of the sample. A supplementary analysis was done with an additional treatment of standard ultrasound before the samples were analysed but without the addition of peptiser. The clay percentages obtained through all different LDPSA methods (Table 2) mostly stay well below the results obtained by decantation, particularly in the clay-rich horizons.

**Table 2:** Clay percentages of eight soil horizons obtained through conventional decantation and Laser Diffraction Particle Size Analyser (LDPSA). The last three rows show results of a second replicate LDPSA analysis, and LDPSA after preliminary sample dispersion. C1 corresponds to the top horizon of profile C (S1 File), C2 corresponds to the second horizon (from the top) of profile C, etc.

	Clay %							
	C1	C2	E1	E2	G2	J2	N2	Q2
**Standard LDPSA 1**	21.6	29.2	24.1	12.8	18.9	11.8	13.5	20.8
**Decantation**	45.9	60.7	33.2	40.6	41.9	16.4	28.6	14.4
**Peptiser + LDPSA**	21.8	27.8	23.9	24.9	25.3	22.4	16.7	25.2
**Ultrasound + LDPSA**	17.0	20.5	15.7	13.7	19.6	19.9	14.3	22.1
**Standard LDPSA 2**	12.6	13.7	13.3	11.5	19.6	20.5	11.6	22.7

The large difference between the measured texture with LDPSA and the decantation measurements shows that LDPSA underestimates the finer fractions (Table 2), as also observed in earlier studies [53–55]. This can somehow be corrected by adding peptiser before LDPSA but the measured clay content still remained far below the values measured by the decantation method for six of the eight samples. Hence, texture obtained through decantation and finger methods will further be used consistently in this study.

The **field capacity** (FC) was measured on the undisturbed samples (in Kopecki rings) with a pressure plate apparatus. After saturation with water, the samples were weighed (M_sat_) and immediately placed in the pressure plate apparatus. A pressure of -1/3 bar (pF 2.53) was applied. When no more water was expelled (after about 7 days) the samples were weighed again (M_FC_), put in an oven at 105°C for 24 hours, and again weighed (M_dry_). Field capacity was calculated as [56]:

$$FC=\frac{M_{FC}-M_{dry}}{V_{kopecki}}*100$$ (1)

in which:

FC = field capacity (%),

M_FC_ = mass of the sample at pF 2.53 (kg),

M_dry_ = oven-dry mass (kg),

V_kopecki_ = volume of the Kopecki ring (l), and

accounting for a water density of 1 kg l^-1^.

The **permanent wilting point** (PWP) was measured with a pressure plate apparatus on disturbed samples. First a paste was made by adding water to about 40 g of soil. The samples were placed in the pressure plate apparatus and a pressure of -15 bar (pF 4.18) was established. Similar to FC measurement, the samples were weighed when no more water was expelled (M_PWP_), and weighed after drying in an oven (105°C) for 24 hours (M_dry_). For each sample, the average of four analyses was taken. The PWP was calculated as [56]:

$$PWP=\frac{M_{PWP}-M_{dry}}{M_{dry}}*\rho_{b}*100$$ (2)

in which:

PWP = permanent wilting point (%),

M_PWP_ = mass of the sample at pF 4.18 (kg),

M_dry_ = oven-dry mass (kg), and

ρ_b_ = dry bulk density of the sample (kg / m^3^).

The **total available water** (TAW) was calculated as [56]:

TAW=FC−PWP (3)

in which:

TAW = total available water (%),

FC = field capacity (%), obtained from (Eq 1), and

PWP = permanent wilting point (%), obtained from (Eq 2).

The **porosity** was calculated as:

$$Porosity=\frac{M_{sat}-M_{dry}}{V_{kopecki}}*100$$ (4)

in which:

Porosity = porosity of the sample (%),

M_sat_ = mass of the saturated sample (kg),

M_dry_ = oven-dry mass of the sample (kg),

V_kopecki_ = volume of the Kopecki ring (l), and

accounting for a water density of 1 kg l^-1^.

The **bulk density** was calculated as:

$$\rho_{b}=\frac{M_{dry}}{V_{kopecki}}$$ (5)

in which:

ρ_b_ = bulk density of the sample (kg / m^3^),

M_dry_ = oven-dry mass of the sample (kg), and

V_kopecki_ = volume of the Kopecki ring (m^3^).

#### Chemical analysis

Before performing the chemical analysis, the disturbed samples were dried at 60°C, the roots were removed, the samples were crushed and sieved at 2 mm.

The **pH**-H_2_0 and pH-KCl were measured after two hours of shaking in water and in a 1 M KCl 1:2.5 solution respectively, with a glass-calomel combination electrode.

The percentage **CaCO**_**3**_ was determined with the ‘rapid titration method’ by Piper [57] was performed. After adding 0.2 M HCL, the solution was titrated the next day with 0.1 M NaOH. The percentage CaCO_3_ was calculated based on the added NaOH. As other carbonates such as dolomite may also be dissolved by this method, the results are referred as ‘calcium carbonate equivalent’ [57].

The **available phosphorus** (Pav) was measured only on the uppermost horizons because P is not very soluble or very mobile. The total amount of available phosphorus was determined by using the Olsen-P method [57]. In this method, the absorbance measured by a spectrophotometer at a wavelength of 720 nm is used to determine the amount P in solution (mg/l), and converted into the amount of P in the soil (mg kg^-1^). However, after centrifuging and filtering of the sample extracts, red-brownish colours were observed in most of the samples and no blue colouring occurred when the mixing reagent was added. This was probably due to the presence of organic matter in the sample extracts. Due to the lacking blue colours no meaningful measurements could be carried out. Three methods were used to try and remove the organic matter but all failed [50]: (i) addition of activated coal (Norit) did not work because large amounts of phosphorus were present in the activated coal; (ii) substituting coal by the polymer polyacrylamide [58] failed to absorb the organic matter; and (iii) the lanthanum (La) precipitation method [59] led to flocculation not only of humic substances but also of phosphorus due to the high phosphate adsorption capacity of lanthanum [60]. Because none of these methods succeeded in filtering the samples without adding or removing phosphorus we chose to measure the influence of the red-brownish colour in the extracting solution on absorbance. For this purpose, we contrasted the standard Olsen solution (standard solution; the extracting solution and the mixing reagent in a 1:1 ratio) with a water solution (the extracting solution and distilled water in a 1:1 ratio). In the water solution no colouring can occur (because no mixing reagent is added) and the spectrophotometer measured the influence of the present colour. In the standard solution the effect of the colouring is measured. By subtracting the absorbance of the water solution from that of the standard solution, the influence of the red-brownish colours was then offset. The influence of the absorbance of distilled water was also taken into account by subtracting this value from the absorbance of the water solution:

$$Absorbance=Abs^{*}-(Abs-water)$$ (6)

in which:

Absorbance = the final measured absorbance,

Abs* = measured absorbance of the standard solution (with mixing reagent),

Abs = measured absorbance of water solution (without mixing reagent), and

water = measured absorbance of a sample of distilled water.

The **electrical conductivity** was measured with a temperature-corrected conductivity meter in a 1:5 solution.

The **cation exchange capacity** (CEC) and **exchangeable bases** were measured with the ‘silver thiourea method’ [57]. The following exchangeable bases were measured: Ca^2+^, Mg^2+^, Na^+^ and K^+^. As the measured pH differs not much from 7, it is assumed that the measured effective CEC (ECEC) is equal to CEC. Base saturation (BS) was calculated as:

$$BS=\frac{\left({exch.Ca}^{2+}+exch.{Mg}^{2+}+{exch.Na}^{+}+{exch.K}^{+}\right)}{CEC}\times 100$$ (7)

in which:

BS = the base saturation (%),

exch. Ca^2+^ = exchangeable Ca^2+^ of the sample (cmol_c_/kg),

exch. Mg^2+^ = exchangeable Mg^2+^ (cmol_c_/kg),

exch. Na^+^ = exchangeable Na^+^ (cmol_c_/kg),

exch. K^+^ = exchangeable K^+^ (cmol_c_/kg), and

CEC = cation exchange capacity (cmol_c_/kg).

High values of exch. Ca^2+^ were found with this method because of partial dissolution of calcite [61]. Due to these high values of exchangeable Ca^2+^, base saturations of more than 100% were found. To solve this problem the values of exchangeable Ca^2+^ were reduced up to reaching a base saturation of 100%.

The **total organic carbon** (TOC) and **total nitrogen** (TN) were determined by combusting pre-weighed samples in a Carlo Erba CHNS-O EA1108 elemental analyser. Before analysis, the carbonates were removed by adding HCL to the samples.

The **interpretation of the measured chemical soil properties** was done using Table 3.

**Table 3:** Interpretation ratings for soil chemical soil properties. Based on Hazelton and Murphy [62].

	Very low	Low	Medium	High	Very high
Ec (dS/m)	0–2	2–4	4–8	8–16	>16
Ca (cmolc/ kg soil)	0–2	2–5	5–10	10–20	>20
Mg (cmolc/ kg soil)	0–0.3	0.3–1	1–3	3–8	>8
Na (cmolc/ kg soil)	0–0.1	0.1–0.3	0.3–0.7	0.7–2.0	>2
K (cmolc/ kg soil)	0–0.2	0.2–0.3	0.3–0.7	0.7–2.0	>2
CEC (cmol_c_/ kg soil)	0–3	3–7	7–15	15–30	>30
Base saturation (%)	0–20	20–40	40–60	60–80	>80
Ntot (g/100 g)	0–0.1	0.1–0.2	0.2–0.3	0.3–0.4	>0.4
Ctot (g/100 g)	0–0.6	0.6–1.2	1.2–3.0	3.0–8.7	>8.7
pH-H_2_O	5–6	6–7	7–8	8–9	9–10
	Moderately acid	Slightly acid	Slightly alkaline	Moderately alkaline	Strongly alkaline
CaCO_3_ (g/100 g)	0–0.5	0.5–2.0	2–5	5–15	>15
Pav (mg/kg)	0–5	6–10	11–14	15–20	>20

### Results

#### Soil chemical properties

The pH_H2O_ of most horizons (Table 4) is around 7 or slightly alkaline, only 5 soil profiles have a moderately to slightly acid pH_H2O._ In all horizons, the pH_KCl_ is lower than the pH_H2O_ except for horizon F2. The CaCO_3_ content varies between low and very high. The EC is very low for all analysed horizons.

**Table 4:** Values of the chemical parameters of the different soil horizons. A1 corresponds to the top horizon of profile A (see S1 File), B2 corresponds to the second horizon (from the top) of profile B, etc.

Horizon	Exch Na^+^	Exch K^+^	Exch Ca^2+^	Exch Ca^2+^*	Exch Mg	ECEC	BS	BS*	pH KCl	pH H_2_O	% CaCO_3_	%N	%C	C/N	EC	Pav
	(cmolc/kg soil)						%	%							μS/ cm	(mg/ kg soil)
A1	0.59	0.35	21.4	17.15	3.21	21.3	119.8	100	7.62	7.79	19.16	0.22	1.89	8.8	191	15.19
B1	0.21	0.21	31.9	29.05	5.46	34.9	108.1	100	6.86	7.28	2.89	0.09	1.52	17.3	135	8.57
B2	0.25	0.30	31.3	28.87	6.58	36.0	106.8	100	6.62	7.15	2.99	0.06	0.89	15.6	91	
C1	0.70	0.65	36.2	31.11	6.08	38.6	113.3	100	6.76	7.5	4.77	0.08	1.37	16.1	167	9.58
C2	0.77	0.42	35.0	30.12	7.60	38.9	112.5	100	6.85	7.61	4.61	0.10	1.39	14.2	187	
D1	0.22	1.09	13.3	13.28	0.97	15.8	98.3	98.3	7.75	8.02	9.57	0.06	0.89	13.7	153	14.61
D2	0.73	1.55	17.6	13.91	4.53	20.7	117.6	100	7.48	8.01	17.39	0.08	0.52	6.6	187	
D3	0.74	0.59	37.8	33.20	1.16	35.7	112.9	100	7.2	7.86	9.12	0.07	0.80	11.3	120	
E1	0.46	0.81	27.3	24.59	5.35	31.2	108.6	100	7.28	7.81	3.26	0.09	0.88	10.2	145	12.03
E2	0.60	0.45	24.9	22.81	7.41	31.3	106.6	100	7.26	7.89	3.17	0.07	0.90	13.3	158	
F1	0.06	0.40	11.1	8.43	0.23	9.1	128.7	100	8.59	8.63	1.78	0.02	0.25	13.5	80	32.38
F2	0.15	0.32	12.6	3.78	6.30	10.6	183.2	100	8.96	8.83	2.19	0.01	0.03	4.1	130	
G1	0.03	0.39	3.9	3.94	0.29	6.5	71.5	71.5	6.97	7.74	1.29	0.02	0.14	5.6	57	10.83
G2	0.25	0.98	10.5	10.53	2.61	15.9	90.4	90.4	7.22	7.65	0.61	0.03	0.21	7.5	125	
H1	0.17	0.48	18.4	18.43	5.10	24.92	97.0	97.0	6.73	7.48	2.54	0.09	0.76	8.8	140	112.68
H2	0.22	0.13	25.0	24.40	13.24	38.0	101.6	100	5.33	7.2	4.55	0.07	0.86	12.7	92	
H3	0.19	0.08	24.2	23.14	13.96	37.4	102.9	100	5.56	7.58	4.51	0.03	0.17	6.4	116	
I1	0.18	0.13	8.4	8.43	3.58	15.0	81.9	81.9	5.61	6.9	1.40	0.05	0.52	11.0	58	12.81
I2	0.12	0.14	4.6	4.59	3.21	10.2	79.0	79.0	5.37	6.62	1.04	0.04	0.39	10.4	133	
I3	0.16	0.16	2.9	2.91	1.99	7.9	66.0	66.0	5.81	6.79	0.75	0.02	0.14	7.2	44	
J1	0.04	0.24	11.4	11.38	4.82	19.6	84.0	84.0	5.31	6.68	1.75	0.07	0.63	9.6	48	24.91
J2	0.15	0.13	9.4	9.40	7.91	17.6	100.0	100	5.25	6.46	1.65	0.05	0.50	10.9	46	
J3	0.11	0.23	8.9	8.90	4.48	16.4	83.9	83.9	5.08	6.31	1.44	0.05	0.41	8.7	59	
K1	0.43	0.14	11.0	11.02	4.60	16.6	97.6	97.6	5.26	5.77	1.17	0.11	0.75	6.6	64	81.11
K2	0.23	0.19	13.8	13.82	5.38	20.0	97.9	97.9	5.05	6.07	1.72	0.11	1.20	11.3	71	
L1	0.02	0.30	1.1	-0.18	3.07	3.2	139.2	100	6.08	6.67	0.43	0.06	0.62	10.6	44	38.25
L2	0.09	0.34	1.8	1.83	0.66	5.5	53.5	53.5	6.64	6.96	0.63	0.02	0.23	9.8	52	
M1	0.12	0.87	6.4	6.39	3.12	11.9	88.0	88.0	5.82	6.7	0.80	0.05	0.50	9.7	56	58.85
M2	0.11	0.81	13.5	13.52	6.14	21.1	97.7	97.7	5.31	6.4	0.86	0.05	0.61	12.7	43	
M3	0.05	0.52	13.8	13.80	6.15	21.3	96.2	96.2	5.51	6.39	0.88	0.03	0.27	8.7	48	
N1	2.29	0.47	10.4	10.40	12.62	28.2	91.5	91.5	6.79	7.49	1.94	0.11	0.98	9.2	46	11.95
N2	14.24	0.40	15.7	9.08	9.87	33.6	119.7	100	7.38	7.81	2.54	0.09	1.45	16.1	140	
O1	0.16	0.58	29.1	28.29	1.70	30.7	102.6	100	7.19	7.75	13.93	0.09	1.32	14.0	146	19.07
O2	0.35	0.69	29.3	26.36	4.65	32.1	109.1	100	7.24	8.03	12.01	0.09	0.96	11.3	138	
O3	0.37	0.52	22.8	22.51	3.50	26.9	100.9	100	7.31	8.07	19.12	0.07	0.39	5.3	186	
P1	0.18	0.86	37.3	33.90	1.92	36.9	109.2	100	7.09	7.65	4.36	0.36	3.96	11.1	191	25.68
P2	0.10	0.75	36.9	33.10	1.20	35.2	110.9	100	7.2	7.81	15.73	0.20	2.69	13.8	177	
Q1	0.11	0.66	29.1	27.77	0.49	29.0	104.4	100	7.45	7.82	19.37	0.21	2.38	11.3	167	16.55
Q2	0.07	0.45	14.2	14.17	0.26	17.2	86.9	86.9	7.75	8.23	18.55	0.30	3.24	10.7	140	
R1	0.13	0.81	29.3	27.94	0.96	29.8	104.6	100	7.56	8.05	19.23	0.19	3.67	18.9	156	13.92
R2	0.09	0.71	20.0	19.98	0.60	21.8	98.2	98.2	7.54	8.16	18.76	0.09	0.81	9.0	128	
R3	0.12	1.48	33.9	29.93	1.76	33.3	111.8	100	7.23	8.04	19.36	0.07	0.54	8.1	139	
S1	0.08	0.98	34.9	28.94	3.62	33.6	117.9	100	7.19	7.83	19.28	0.16	2.43	14.8	171	19.11
S2	0.22	0.67	35.6	33.38	2.49	36.8	106.1	100	6.91	7.58	4.45	0.30	4.07	13.5	216	
T1	0.07	3.69	26.3	23.94	1.91	29.6	107.9	100	7.19	7.72	19.06	0.19	1.86	9.7	162	42.77
T2	0.20	1.81	25.4	25.43	1.43	29.4	98.1	98.1	7.31	7.82	19.09	0.18	1.75	10.0	148	

*: adjusted value for exchangeable Ca^2+^ and BS.

Ca^2+^ is the dominant exchangeable base followed by Mg^2+^; both have high to very high values in most horizons (Table 4). The values for Na^+^ and K^+^ range between very low to high but most horizons have a medium value. ECEC values are high or very high for most horizons. The base saturation is very high in all horizons; most have a value close or equal to 100%.

The %C in most horizons is low to very low but some horizons have high values. The N content is low to very low for almost all the horizons. The measured available P is highly variable between the different horizons but most horizons have medium or high values.

#### Soil physical properties

The average field capacity is 27% (± 7%), with a range between 8 and 37%. Values per horizon are presented in the profile descriptions (see S1 File) and have been tabulated by Tielens [50]. The average permanent wilting point is 21% (± 8%), with a range between 4 and 38%. The average total available water (TAW) is 7% (± 5%), with a range between 1 and 16%. In several horizons the calculated TAW was slighty negative, indicating that the values of PWP and FC were close to each other. In such cases, TAW was not further taken into account. The average prosity is 42% (± 7%), with a range between 30 and 54%. Like for the other soil physical parameters, these descriptive statistics concern all profiles and horizons. The average bulk density is 1.34 (± 0.24 g cm^-3^), with a range between 0.91 and 1.74 g cm^-3^.

### Discussion

#### Soil chemical properties

In neutral soils, the exchangeable base complex is dominated by Ca^2+^ and Mg^2+^, in alkaline soils Na^+^ and K^+^ are more present and in acid soils Al^3+^ and H^+^ are the most abundant [63]. The profiles with a slightly to moderately acid pH (I, J, K, L and M) have indeed significantly lower values of exchangeable K^+^ and Na^+^ (0.32 cmol_c_/kg K^+^ and 0.16 cmol_c_/kg Na^+^) in contrast to 0.75 cmol_c_/kg K^+^ and 0.76 cmol_c_/kg Na^+^ in the other profiles. For Ca^2+^, also a significant difference exists between the two groups: 8.14 cmol_c_/kg for the acid soils compared to 22.18 cmol_c_/kg for the alkaline soils. For Mg^2+^, no significant difference was found between both groups.

When interpreting the soil chemical properties (Tables 2 and 3), the N content of all samples is low to very low (0.01–0.22%, with an outlier of 0.36 in Des’a forest), in line with limited inputs of N [64], high erosion rates and prolonged cultivation. N is the most limiting soil chemical property and increasing the N content is a must to increase crop yields. Even though farmers with livestock dispose of the organic form of N (manure), it was observed that this manure is stored and used in a poor way. In many cases the manure is left exposed to the weather so rain can leach all the valuable nutrients. In other cases, it is thrown away, particularly in the eastern part of the catchment, or it is dried and used as a fuel. The inorganic form (mineral fertiliser) was less popular [29] because of its high cost [64, 65], as well as unreliable rainfall.

Values of soil OC are also low (0.03–1.9%) except in Des’a forest where a mean value of 2.6% was found. Such low soil OC contents are the consequence of severe soil erosion, limited inputs (manure or crop residues) and overgrazing, which results in a low vegetation cover [3, 66]. In line with the OC content, the soil organic matter (SOM) plays an important role in the soil: it improves structure, water holding capacity, nutrient absorption and release to plants [67]. The higher values in Des’a forest are the consequence of the nearly absent soil erosion, and the vegetation cover that leads to larger biomass inputs. Exclosures also trap upslope eroded sediments, which have very high organic carbon content, allowing a fast regeneration of soil productivity [44].

#### Soil physical properties

As may be expected in these landscapes that hold an extremely varied lithology, there are strong contrasts in soil texture. Sandy soils occur in profiles derived from sandstones, silty on the precambrian metamorphic rocks. Despite the fact that the inadequacy of the LDPSA laboratory analysis hampered the study, the decantation analysis showed that soils and horizons with clay contents beyond 40% are common (Table 2). For some part, they represent the pristine soil before major human interventions, either in the topsoil (profiles B, C, J and N) or as a buried horizon (profiles G and K). The mountainous nature of the topography led to frequent occurrence of colluvium, but pedogenesis on such colluvium is commonly leading to textural fining in the top horizons.

Additionally, mass movements have in many places transported materials from the basaltic uplands over the lower-lying sedimentary rocks, increasing the opportunity for clay soils to develop (see Part II).

Overall, the measured TAW (7.6% ± 4.9%) is lower than the expected 10 to 20% for silty clays and clay soils, or 15 to 25% for silt loam, loam and silty clay loam [68]. Such low values for TAW could be the result of low values for FC or high values for the PWP. As PWP measurements, done under extremely low pressure, are subject to errors, our measurements were repeated four times and found to be consistent.

On the other hand, the relatively low FC values (average of 27%) are most probably related to the space occupied by the frequent small rock fragments in the soils (and in the undisturbed samples alike). For instance, a 40% rock fragment content in soils has been demonstrated to reduce the field capacity by 50% [69]. Low TAW is, hence, explained by the stoniness of the soils.

Similarly, Descheemaeker et al. [70] analyzed TAW of top horizons of Cambisols, Calcisols and Phaeozems in exclosures and eucalyptus forest in the Giba catchment. In these soils with relatively high clay percentages (average of 37%) and relatively high SOM content (average of 3.7%) [70], the average TAW was 12% (± 2%) [70]. Taking into account the higher OC content, capable of holding larger amounts of water [71], these values are still at the lower limit of what may be expected based on texture [68]. Overall, the TAW for plant roots is strongly affected by the stoniness of many soils.

## Part II. Soil profiles

### Methodology: Soil auger and profile pit observations

In total 1381 soil auger and 141 profile pit observations were made in the catchment. The exact location of these augering sites and profile pits were determined by discussion among authors, and based on the reconnaissance studies and the interpretation of the digital data. These observations were made along soil catenas across the whole catchment.

The augerings were made with an Edelman auger. If augering depth was limited due to stoniness of the profile, multiple augerings were conducted and the deepest profile was described. For each augering the following properties were recorded: position; elevation; slope gradient; land use; depth; parent material; soil texture (assessed by finger test).

The profile pits were described in detail using the FAO guidelines for soil description [72]. The following properties of the different soil horizons were characterised: depth; colour (Munsell Soil Colour Chart); texture; structure; stickiness; distribution of roots; reaction with HCl; surface stoniness. The profiles were classified according the World Reference Base for Soil Resources [73].

Local land users provided additional information–individuals who appear on profile pit photographs have given written informed consent to publish the photograph. Among the 141 soil profiles, 20 are presented in detail, well distributed over the different geomorphic regions of the Giba catchment (Fig 3).

### Soils on basement and Palaeozoic (fluvio-)glacial deposits

#### Precambrian lithology and Palaeozoic sedimentary rocks

The basement rocks of the catchment belong to the Arabian-Nubian shield [74, 75], which was formed as a consequence of the collision of East and West Gondwana causing low-grade metamorphism of the rocks [76]. They cover about 27% of the catchment and 6 different units exist: granitic intrusions, metalimestone, metasediments, metaconglomerate, metagreywacke and metavolcanic rocks.

**Fig 4:**
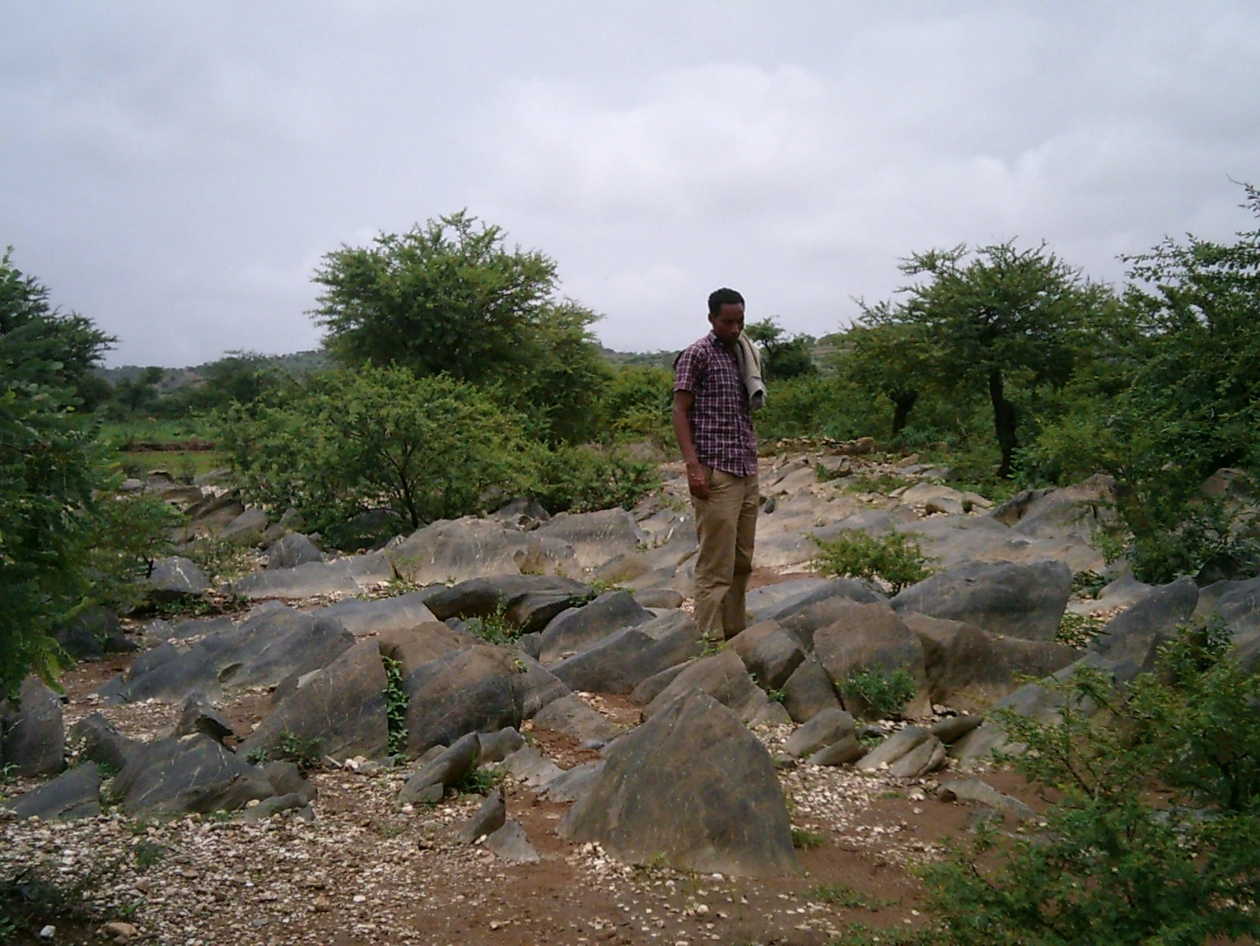
Black meta-limestone outcropping near Taget. Note the presence of white quartz fragments at the soil surface that originate from quartz veins.

The Precambrian metalimestone is blackish (Fig 4) or light grey to white and has quartz veins. It can be found in the Negash syncline where it has undergone strong folding and in the Abergelle lowlands [31].

The metasediments are phyllites and slates, both the result of low graded metamorphism of shale. Both are very fine grained and can be found in the same areas as the metalimestone. The slates are reddish or greyish, very cleavable and quartz veins are common. The reddish colour is due to the presence of hematite (Fe_2_O_3_). The phyllites are more metamorphosed than the slates, which explains the shiny surface, but in contrast to the slates they are not cleavable.

Metaconglomerates are only found in the northern part of the catchment, around Negash and on the Atsbi horst. The metagreywacke is mainly limited to the Atsbi horst. It is coarse grained and probably derived from pyroclastic materials ejected during back-arc volcanism [74, 77]. The sedimentary structure, which distinguishes them from the metavolcanic rocks, is explained by the transportation and reworking by running water [31].

As metavolcanics, both acidic and basic volcanic rocks occur in the western part, near the outlet, in the north around Negash and on the Atsbi horst and show a fine to medium grained texture [78].

Coarse-grained granitic intrusions are the youngest Precambrian formation in the Giba catchment [74]. Its spatial coverage is limited, the most extensive exposures are found west of Negash (almost 50 km^2^), around Abiy Addi and at the outlet of the catchment. These intrusions are a possible source of the granite boulders in the Edaga Arbi tillites.

Palaeozoic (fluvio-) glacial deposits are the oldest sedimentary rocks in the catchment. They are unconformably overlying the first planation surface formed on the basement rocks [33]. Two different units can be found: the Edaga Arbi tillites and the Enticho sandstone. Generally, the tillites overlay the Enticho sandstone, but the two are often interfingering [31].

The Edaga Arbi tillites consist of poorly sorted, unstratified and poorly consolidated fine-grained sediments (silt- to claystones) with colours varying from red, purple to dark grey and black [31, 79]. At some locations varved proglacial deposits can be found. Another evidence of a glacial environment is given by the presence of dropstones of various sizes [79–81]. Glacial landforms like roches moutonnées also occur, with the presence of striations, grooves and chattermarks in the underlying rocks, indicating direct ice contact [79, 81, 82]. The area covered by tillites is small (<1%), and is located near Abiy Addi, Wuqro and Idaga Hamus.

The Enticho sandstone is a white, medium- to coarse-grained sandstone and is characterised by cross beddings [31]. Deposited as glacial outwash [81], it unconformably overlies the basement rocks. Precipitation of iron at the contact between layers made it very resistant to erosion and is the reason why plateaus of Enticho sandstone stand out in the landscape. The Enticho sandstone covers a large area (8%), particularly in the northern part of the Giba catchment.

#### Soils in the Abergelle lowlands

**Fig 5:**
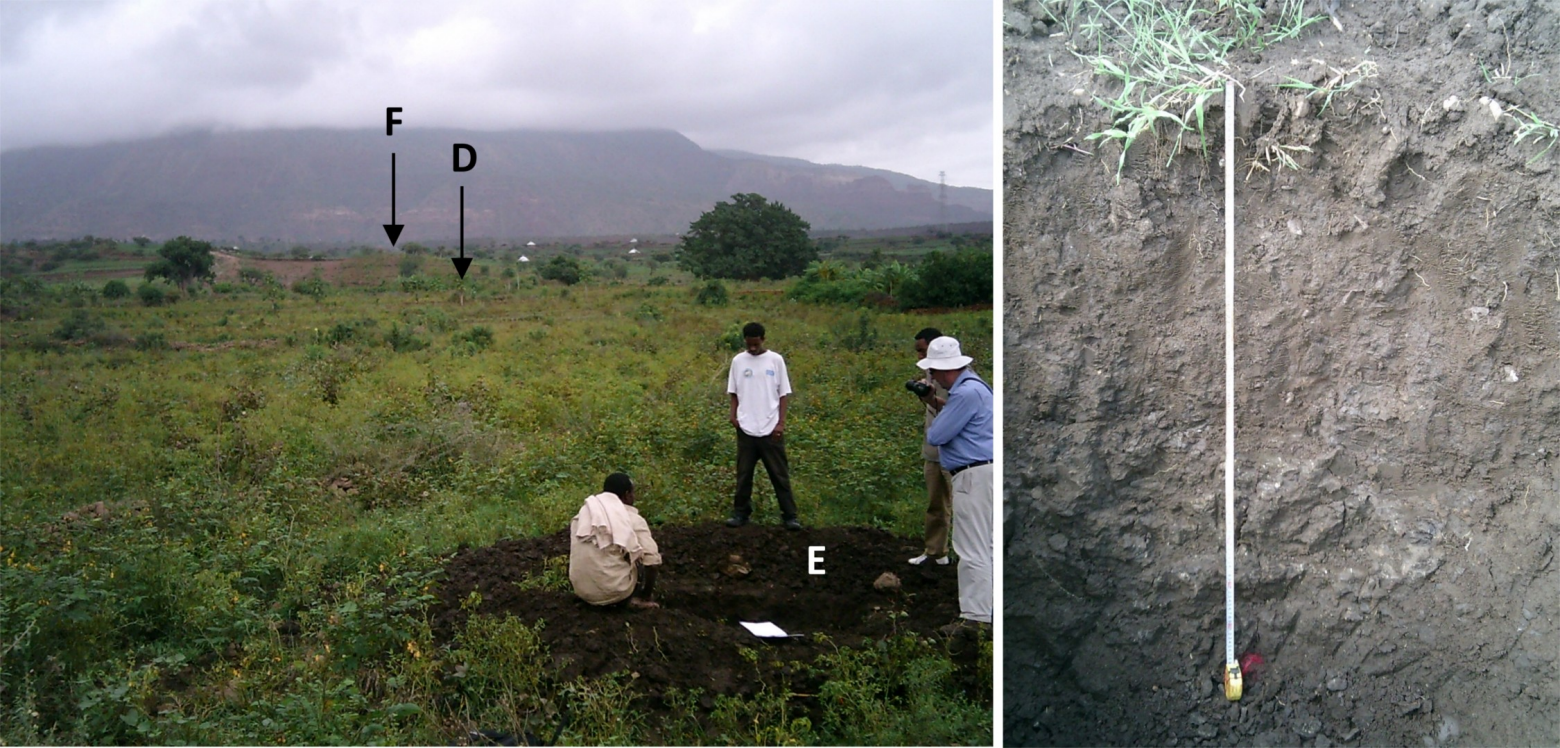
**Location of soil profile pit E in Agbe, Abergelle lowlands (left) and profile E (Chromic Vertisol, right).** At the back, the Adigrat sandstone cliff is visible; arrows indicate the approximate locations of soil profiles D and F.

The Abergelle lowlands are in the western part of the catchment (Fig 3). This area is dominated by Precambrian rocks including metalimestone, metasediments (phyllite and slate), metavolcanic rocks and granitic intrusions [31]. In the east, the geomorphic region is confined by a steep Adigrat Sandstone cliff (Fig 5, left). Typical for the Precambrian rocks are the occurrence of many small rounded hills that are mainly aligned in a NE-SW direction. The vegetation cover is limited due to lower precipitation and higher mean annual temperatures, and provides little protection against erosion by water which resulted in shallow soils. The dry climatic conditions and shallow soils make this area not suitable for cultivation. Most of the land is bare land or rangeland. Deeper, mostly cultivated, soils are observed in the valley bottoms, often corresponding to areas with metalimestone as parent material.

Towards the outlet, the landscape is strongly incised by the Giba River which is reflected in a very rugged terrain with steep slopes, shallow soils and very limited cultivation. At the foot and toeslopes of the steep Adigrat Sandstone cliff, Palaeozoic tillites outcrop and a 0.01 to 10 m thick layer of colluvium has been deposited.

Profile A: Calcaric Rendzic Leptosol. Profile A is situated on the shoulder of a small metalimestone hill (Fig 4) in Taget, Abergelle. Despite the rather steep slope, the shallow soil and the very high topsoil stoniness, this area is used for (marginal) cultivation. The parent material is strongly weathered metalimestone. Chemically this soil is rather rich and characterised by very high CaCO_3_ values (19%). The contents of Ca^2+^ (17.15 cmol_c_/kg) is very high and Mg^2+^ (3.21 cmol_c_/kg) high (Table 3). The available P (15.19 ppm) is high and the C (1.9%) and N (0.22%) contents are medium. The texture is silt loam, but many small rock fragments are present, which reduce the water holding capacity. This soil profile, like all others, is described in detail in the S1 File, which contains also all analytical data.

Profile B: Epileptic Proto-vertic Cambisol. This soil profile is at the toeslope of the same metalimestone hill as profile A. The soil depth is limited to 45 cm. The rock fragment content is less and almost no rocks occur in the B horizon. The parent material is the same strongly weathered metalimestone. All the soils in the area are used for cultivation. The CaCO_3_ content is medium (2.9%) and the pH (7.2) is slightly alkaline. The higher clay percentages result in very high effective cation exchange capacity (ECEC) values (35.5 cmol_c_/kg) which makes this soil rather fertile. The available P (8.57 ppm) and total N (0.07%) contents are however low and very low. The clay has swell-shrink properties as evidenced by small but not completely developed slickensides. Soil that is located near one of the numerous termite mounds gives better crop yields according to the farmers and the mounds are therefore not destroyed [83].

Profile C: Pellic Vertisol. Profile C is situated about 0.5 km east of profile B in the valley bottom where thick black clays cover metalimestone. Small limestone fragments can be found throughout the profile. The whole area is intensively used for cultivation (sorghum). The very high ECEC value (ca. 38 cmol_c_/kg) makes this soil chemically very rich. The organic carbon content (1.4%) is medium but the available P (9.58 ppm) and N (0.09%) contents are low and very low respectively. The clay percentage in the B horizon (61%, in contrast to 46% in the A horizon) is very high and clearly developed slickensides are visible. The swell-shrink properties of the clays result also in an angular blocky structure. At the surface, black metalimestone fragments (up to 1 m across) can be found. Five-centimetre wide and up to one-metre deep cracks develop in the topsoil during the dry season. The surface horizon meets the requirements of the qualifier ‘Grumic’ (strong fine granular structure) but it was chosen not to use this qualifier because the structure might have been caused by recent ploughing.

Profile D: Colluvic Calcic Luvisol. This profile is situated in a gently sloping area about 2.5 km south of the Adigrat Sandstone cliff (indicated by an arrow on Fig 5). The parent material is Adigrat Sandstone colluvium. Besides sandstones, also basalt fragments are found at the surface which confirms the colluvial origin. Maize is cultivated on this location. The colluvial origin can explain the loamy nature of the A horizon. A clear clay jump occurs in the B horizon, which meets the requirements of an ‘argic’ horizon. In this horizon many small CaCO_3_ concretions were observed. Chemically this soil, and particularly the B horizon, is dominated by a high CaCO_3_ content (12.1%) and Ca^2+^ (20.3 cmol_c_/kg) values; it was classified as calcic. The ECEC (24 cmol_c_/kg) is also high but the exchange complex is mainly dominated by Ca^2+^. The organic C (0.71%) and total N (0.07%) contents are low to very low, as reported earlier on for the wider region [84]. The available P (14.61 ppm) is medium.

Profile E: Chromic Vertisol. This profile is situated less than 1 km downhill (south) of profile D (Fig 5). In contrast to profile D, no sandstones but only basalt fragments were found. The area is used for irrigated agriculture; cotton and red pepper are intercropped. Chemically the soil has very high ECEC values (31.24 cmol_c_/kg). The very high values for Ca^2+^ (23.4 cmol_c_/kg) and Mg^2+^ (6.4 cmol_c_/kg) are typical for soils derived from basalt. The clay percentages are also high (33% to 41%) and clear slickensides can be observed in the B horizon which indicates the swell-shrink properties of the clay. Like the other soils, the levels of organic C (0.90%) and N (0.09%) are low to very low.

Profile E was classified as a Vertisol. Other Vertisols were also found in the area (profile C) but the location of this Vertisol is remarkable: it is found a few km downslope from the Adigrat Sandstone cliff whereas the whole surrounding area is covered by reddish, sandy colluvial material of this cliff. In stead, the Vertisol developed on materials derived from basalt; as a mafic rock, it is rich in Ca and Mg and therefore an ideal parent material for smectites [85]. The nearest potential source of basalt are the Hagere Selam highlands: 5 to 6 km north of profile E and are almost 1 km higher (on top of the escarpment depicted in Fig 5). Most probably, a debris flow transported these sediments to the lowlands, the path of which can be identified on aerial photographs [50]. Debris flows in the Hagere Selam highlands [86] are capable of transporting debris over much larger distances than local landslides. The fact that a Vertisol developed on the deposited basalt-derived sediments indicates that the debris flow occurred at least several thousand years ago [87, 88]. Humid periods favourable to pedogenesis existed in the study area roughly between 10 000 and 5000 yr BP and between 2500 and 1500 yr BP [3]. It is therefore likely that the Vertisol formed during one of those two periods.

Profile F: Lithic Leptosol. Profile F is situated about 1 km uphill (north) of profile D, closer to the Adigrat Sandstone cliff (Fig 5). The parent material is strongly weathered Adigrat Sandstone. Because of the very limited soil depth this area is used as rangeland but the vegetation cover is sparse. The sandy characteristics result in a chemically very poor soil with ECEC values around 10 cmol_c_/kg. The amounts of organic C (0.12%) and N (0.01%) are very low.

**Fig 6:**
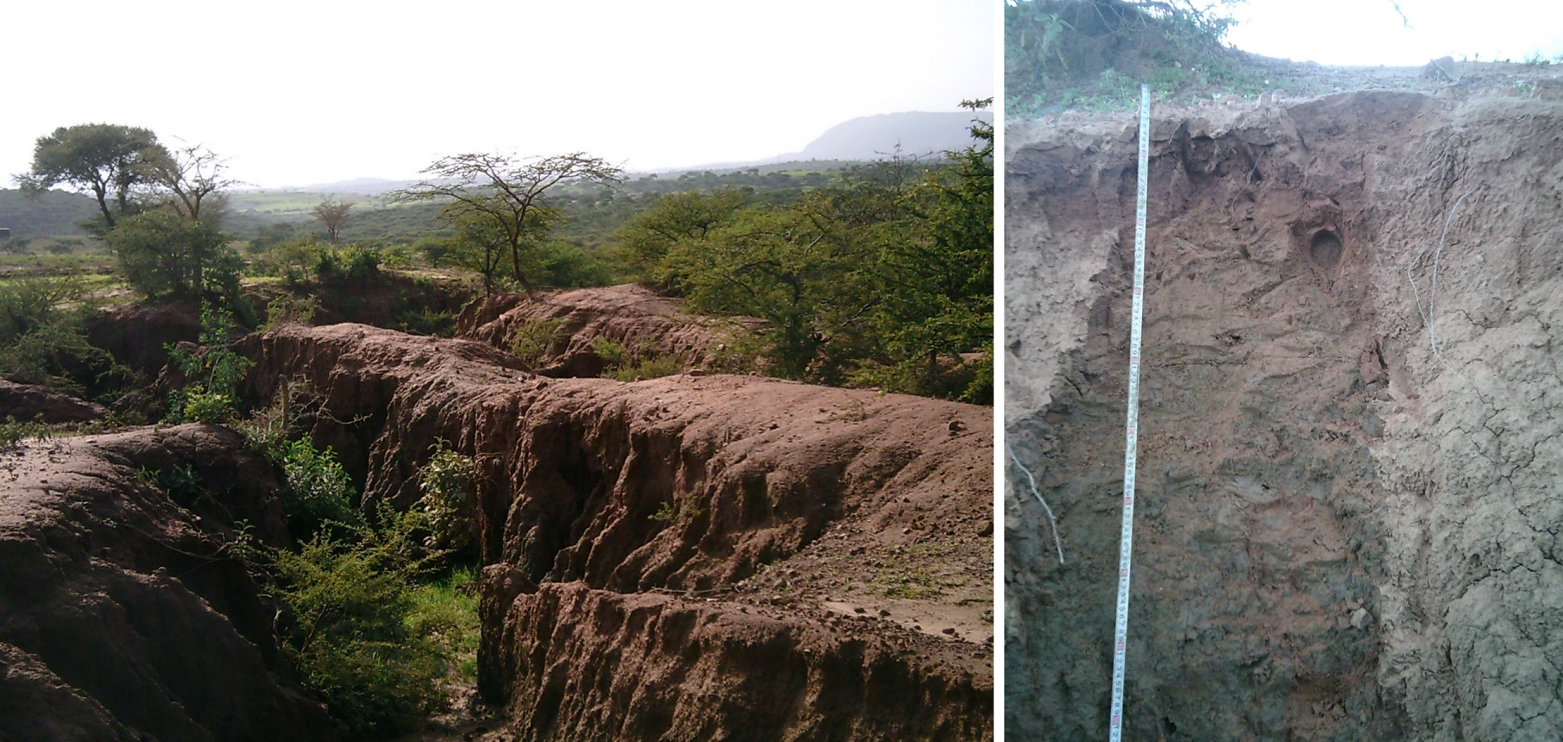
**Location of soil profile pit G** in sandstone colluvium overlying tillites, south of Abiy Addi (left) and profile G (Haplic Planosol, right).

Profile G: Haplic Planosol. This profile is in a large gully, close to the Adigrat Sandstone cliff south of Abiy Addi (Fig 6). The area is, like profile F, used as rangeland and dominated by small trees and shrubs. The A horizon is sandy, but at a depth of 40 cm a very abrupt textural change occurs, with 42% clay in the B horizon. Chemically this soil is very poor. The lower, clayey layer (below a depth of 40 cm) has a higher ECEC (15.9 cmol_c_/kg) but organic C (0.17%) and N (0.02%) content is very low. We classified this soil as a Planosol, given the abrupt textural change in the soil profile [73], between the coarser uppermost layer and the underlying one.

No mineralogical analysis was conducted in this research, but the high pH (around 7) and high base saturation (BS > 71%) indicate that the actual conditions are not suitable for ferrolysis, in line with suggestions elsewhere in Ethiopia [89]. Given the location of these Planosols, the geogenetic process [85] is the most likely cause: they are situated very close (within 1 km) of the Adigrat Sandstone cliff. The coarse surface layer is a colluvial deposit from the cliff, similar to the surface layer of profile D. The finer textured layer underneath is a clayey layer of the Edaga Arbi tillites that outcrop under the sandstone cliff. Hunting T.S. [49] presents a description of a profile (PE/9) that is very similar to the above-described Planosol, in a similar geomorphic setting.

Soil types observed in Aqushala in the Abergelle lowlands [45] comprise (1) in the metamorphosed black limestone, Endoleptic Calcisol at the upper slope; Endoleptic Cambisol and Vertic Leptosol at the middle slope, Hypercalcic Calcisol at the footslope and Grumic Vertisol in the valley bottom; (2) in the metasediments, Leptosol at the upper and footslope, Regosol at the mid slope position and Fluvisol in the valley bottom; and (3) in metamorphosed banded marl, Leptic Calcisol at the upper slope, Haplic Calcisol at the foot slope, and Fluvisol in the valley bottom. The soil-landscape model was successfully tested in the Taget area (where profiles A, B and C are located).

#### Soils on the Precambrian and Palaeozoic rocks of the Atsbi horst

**Fig 7:**
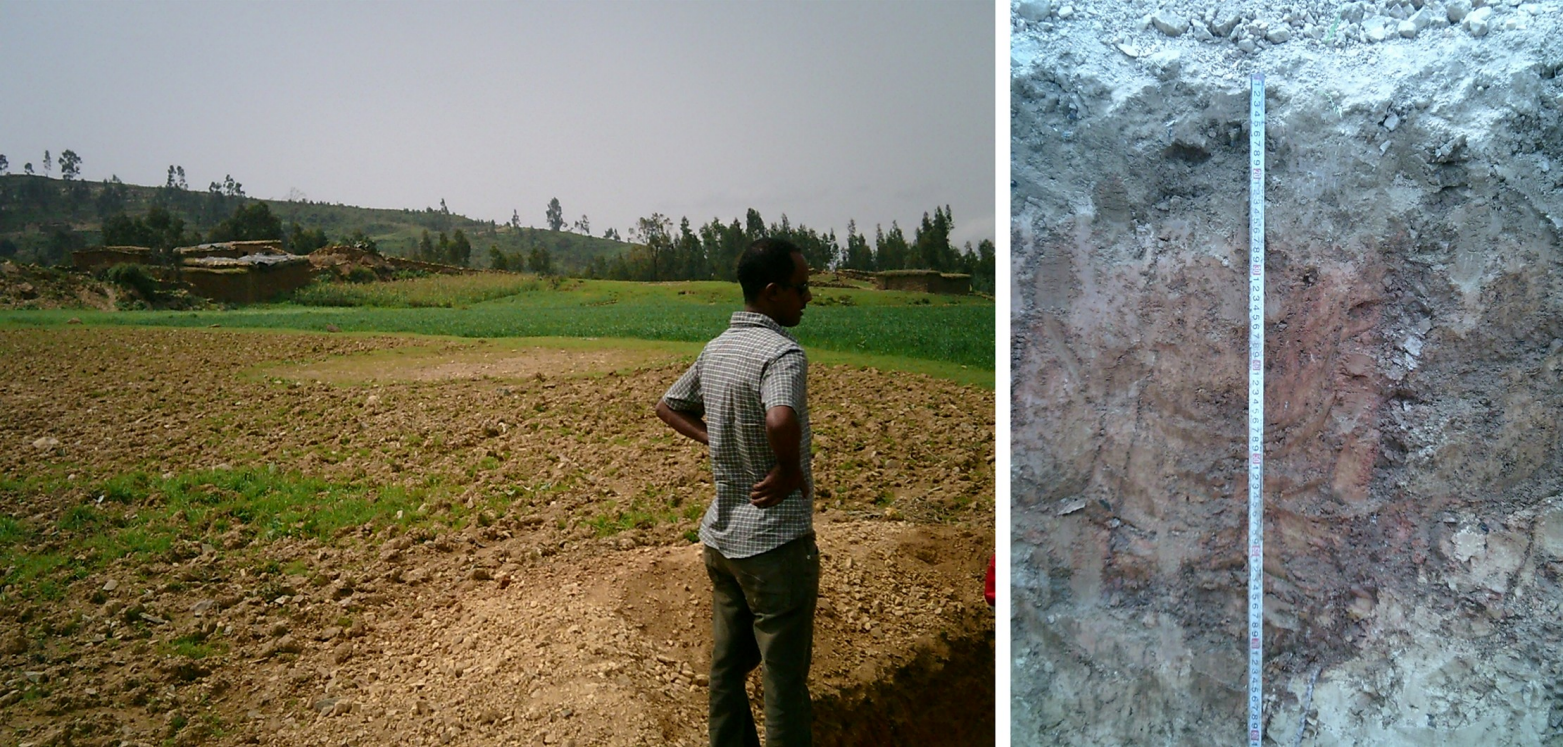
**Location of soil profile pits H (in front) and I (Leptic Cambisol, at the foot of the hill) on the meta-sediments of the Atsbi horst (left) and soil profile H (Leptic Luvisol, right)**.

The Atsbi horst is in the north-eastern part of the catchment (Fig 3). At the west, it is demarcated by the Negash geosynclinal fold and a major normal fault [90]. Both the fault and fold lines are running north-south [31]. In the south, the horst is bordered by the younger Wuqro fault belt. In the eastern, northern and locally in the central part Enticho sandstone outcrops occur. Due to differential erosion, the Enticho sandstone now stands out in the landscape and forms mesas or smaller buttes [16, 49]. However, most of the area is covered by Precambrian rocks: metaconglomerate, metagreywacke, and dominant metavolcanic rocks. Almost all the land except for the steep slopes is under cropland (Fig 7).

Profile H: Leptic Luvisol. This profile is situated on the Atsbi Horst on the level top of a ridge. The parent material is strongly weathered metasediment. Despite of the level topography, soil depth is limited to 60 cm. Chemically, this soil has high ECEC values and in the B horizon even very high values (38 cmol_c_/kg) were measured. The values of Ca^2+^ (22.0 cmol_c_/kg) and Mg^2+^ (10.7 cmol_c_/kg) are high to very high and the pH (7.3) is slightly alkaline. In the B horizon, a clay jump occurs and this horizon is classified as ‘argic’. The organic C (0.6%) and total N (0.06%) contents are low to very low. The top horizon has the highest measured value of available P (112.7 ppm) of all surface horizons. Despite the loamy nature of the C horizon, this horizon has a very high ECEC value (37.4 cmol_c_/kg).

Profile I: Leptic Cambisol. Profile I is situated at the footslope of the same metasediment hill as profile H (Fig 7). Despite its 10% slope gradient, this area is used for cultivation. The surface stoniness is very high and besides metasediments and metavolcanic rocks, quartz fragments are abundant. Chemically, this soil differs greatly from profile H. The amounts of exchangeable cations and the ECEC values (10.4 cmol_c_/kg) are much smaller. Over the entire profile, this soil has one of the lowest measured base saturation (52.7%). The CaCO_3_ content (1.0%) is low and the pH is slightly acidic (6.8). Both the organic C (0.35%) and total N (0.03%) are very low, and decrease with depth.

Profile J: Haplic Cambisol. Profile J is 1.5 km west of profiles H and I, almost in the valley bottom, covering metavolcanic rock. The slope is very gentle and the soil is much deeper than the previous two profiles. Similar to profile I, quartz fragments occur at the surface but are less abundant. At the surface 2–3 cm of overwash was observed. Small nutty structures were observed in the B horizon, but the clay percentages are too low to consider it as a nitic horizon. Consequently, this soil is chemically not as rich as profile H in ECEC and Ca^2+^. The pH is slightly more acid (6.5) than profile I. Like most soils in Giba catchment, this soil has very low to low values of C (0.5%) and N (0.05%). The available P (24.9 ppm) content, however, is very high.

Lithic Leptosol, Leptic Cambisol and Leptic Regosol were also observed on and near rock outcrops in the Ruba Feleg and Kuret sub-catchments whereas in areas where thin colluvium of Enticho Sandstone covers the Precambrian, associations of Haplic Cambisol, Haplic Regosol and Skeletic Regosol were observed [41].

#### Soil profiles in the Sinkata midlands

The Sinkata midlands are in the north-western part of the catchment. They start north of the Wuqro fault and extend northwards to the basalt-dominated highlands of Mugulat. In the East, the midlands are bounded by the Atsbi horst. The midlands can be subdivided into three units with their own geological and geomorphic characteristics.

**Fig 8:**
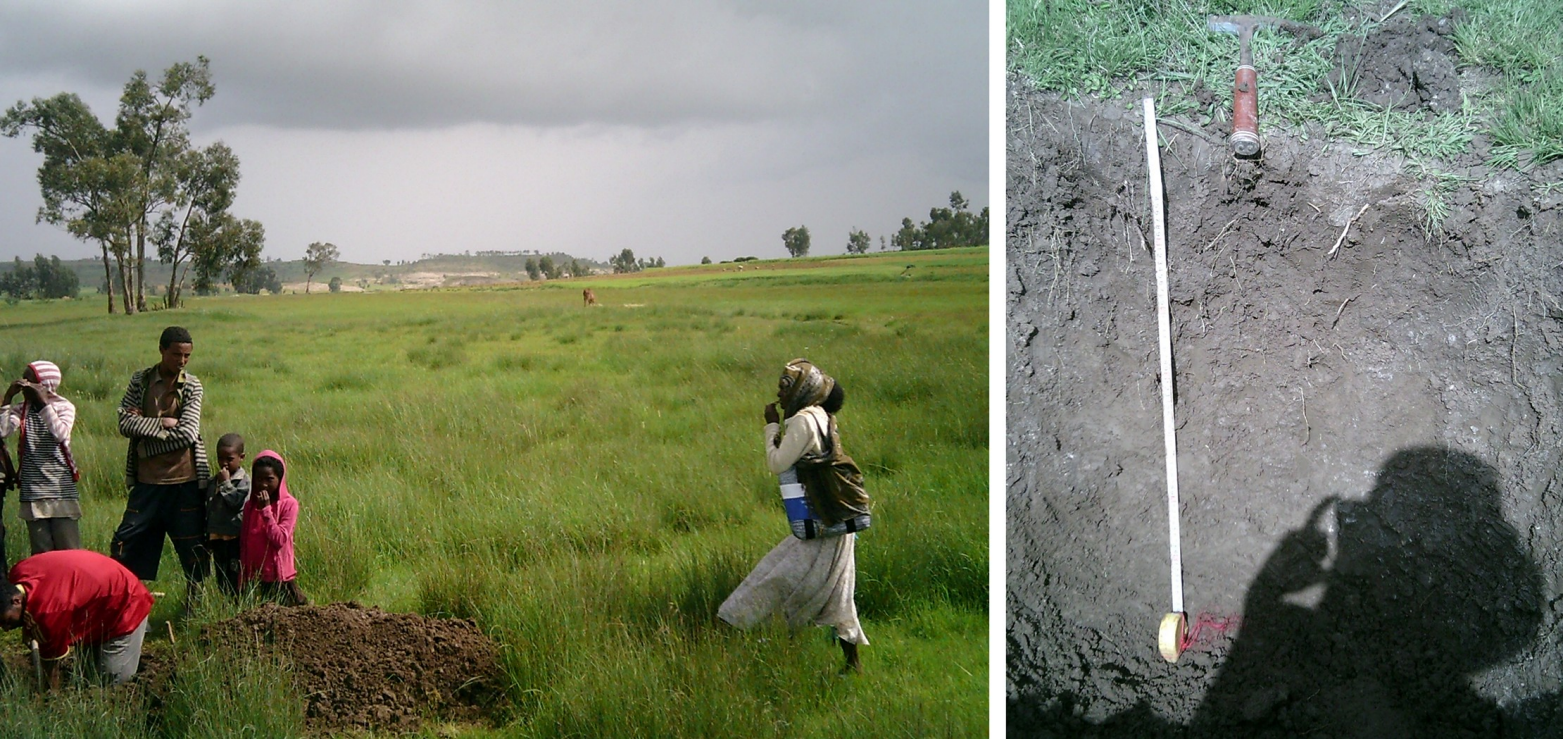
**Location of soil profile pit N in the Sinkata midlands (left) and profile N (Mazic Socid Vertisol, right)**.

The first unit, in the northern part, is covered by Enticho sandstone, with occurrences of Edaga Arbi glacials in the lower positions. The area is a very gently undulating plain and most of the area is used as agricultural land (Fig 8, left). A relatively high base saturation was observed in these soils despite a high quartz content which has been attributed to the calcitic cement in the sandstone [30]. Leaching of the bases on the higher sites and accumulation in the depressions together with selective downslope transport of finer particles resulted in coarse-textured and more acidic soils on the higher sites and finer textured and base saturated soils in the depressions [30]. At some locations the soil is very shallow where the Enticho sandstone outcrops with its protective ferruginous sandstone cap. Frequent occurrence of rounded pebbles does mostly not indicate fluvic properties, as such pebbles occur as lenses in the Enticho sandstone, and throughout the tillites.

The second unit can be found south of the previous unit and is covered by Precambrian rocks and some smaller Enticho sandstone outcrops. The metavolcanic rocks cover the largest area, followed by metaconglomerate. In the centre, a large granite batholith is present [33] which forms a series of concentrically oriented ridges with narrow intervening valleys [49]. These ridges are composed of very rocky tors and are thus not suitable for cultivation; in the wider valleys the soil depth allows cropping.

The last unit is the Negash synclinorium [90] and consists of metasediments and metalimestone. The landscape is very steep with locally alluvial terraces with deeper soils which are used for cultivation.

Profile L: Arenic Lixisol. Profile L is situated near the town of Sinkata on a small plateau. Weathered Enticho sandstone is the parent material. Just like most of the northern part of the Sinkata midlands this area is used for cultivation. Chemically this soil is the poorest of all described profiles. The ECEC of the B horizon is low (5.47 cmol_c_/kg). Due to the presence of a clay jump, the B horizon was qualified as an ‘argic’ horizon. Organic C (0.4%) and total N (0.04%) are again low to very low due to the agriculture practices, but the available P content (38.25 ppm) is very high. In view of the presence of an argic horizon, and base saturation of more than 50%, this soil is classified as a Lixisol. In the entire profile small, reddish iron nodules/coatings were observed.

Profile M: Haplic Fluvisol. This profile is situated almost in the valley bottom of the Sinkata midlands, on grazed fallow land, but the adjacent lands were cultivated. Fluvic material was found beneath the shallow A horizon. Two different horizons, separated by a gravel layer (sandstone and metavolcanics) at 40 cm depth, can be distinguished in the fluvic material. Chemically the A horizon differs from the underlying horizons. The ECEC values in the fluvic horizons (21 cmol_c_/kg) are almost double of the uppermost (12 cmol_c_/kg) horizon. The percentages of organic C (0.4%) and total N (0.04%) are very low in the entire profile. Like profile L, the entire profile is slightly acid (pH of 6.5). At the bottom of the profile strongly weathered sandy material with purple colours was present.

Profile N: Mazic Sodic Vertisol. This profile is situated a few metres from profile M but in the thalweg (Fig 8). In contrast to profile M this area is used as permanent grassland. A few metres further, ground water was observed in a small depression but no groundwater nor gleyic colour patterns were observed in the profile. Due to the hardness of the soil, the profile was only excavated to a depth of 80 cm. Chemically and physically this soil differs strongly from profile M. The texture is more clayey and becomes very hard upon drying. In the B horizon, not fully developed slickensides could be observed, indicating vertic properties. At the surface, a gilgai microrelief was observed although it was not very distinct.

ECEC values (30.1 cmol_c_/kg) are high to very high as can be expected with swell-shrink clays. However, the most prominent characteristic are the very high values of exchangeable Na^+^. In the B horizon, this value is even extremely high (more than 14 cmol_c_/kg). 42% of the exchange complex is occupied by Na.

In the Tsinkaniet catchment of the western Midlands, Tesfu Woldegerima (48) found Endoeutric Cambisol and Haplic Regosol on 10–15% slopes, Haplic Leptosol on 5% slopes, Cutanic Luvisol and Mazic Vertisol on 1% slopes, and Arenic Fluvisol along the rivers. The soil-landscape model was applied in another similar area (Sendeda Guims), with a rate of accuracy of 30%. Thin-section analysis showed the presence of soils with an argic horizon in the plains and on plateaux which were mainly classified as Lamelli-Arenic Luvisol [91]. Hunting TS [16] presents profile descriptions for Chromic, Eutric, and Vertic Cambisols as well as for a Chromic Vertisol and a Cambic Arenosol in the wider Hawzien area (just outside and northwest of the Giba catchment, but part of the Sinkata Midlands).

### Soils on Mesozoic sedimentary rock

#### Mesozoic rocks

The Mesozoic rocks cover more than half the catchment (53%) and are the result of a transgression-regression cycle. During the transgression and regression two sandstone formations were deposited: the Lower (Adigrat) and Upper (Amba Aradam) Sandstone formations. During the transgression period, the wider area was below sea level and the Antalo Formation was deposited, which consists of limestone, shale, marl and minor intercalations of gypsum layers [92].

The Adigrat Sandstone has a maximum thickness of nearly 700 m around Abiy Addi and it is further exposed around Wuqro, Hayki Meshal, north of Idaga Hamus and in some deeply incised gorges of the Giba R. and its tributaries [31, 93].

The Antalo Formation can be found south of the Wuqro fault belt, all the way to the southern part, and in the western part it stops around Hagere Selam. The thickest depositions in the east are estimated at 1100 m; the formation pinches away towards the west [31].

The upper sandstone formation (Amba Aradam) is a near-shore deposition with cross beddings. The sandstone is reddish coloured which indicates the presence of oxidised iron (hematite and magnetite). It is fine grained and overlain by flood basalts which created a ‘baked’ contact in the upper part of the formation [94, 95], leading to induration and low permeability. The maximum thickness of the Amba Aradam Sandstone is around 50 m; it covers <1% of the catchment, particularly in the Hagere Selam highlands and on the Amba Aradam mountain in the southern part of the catchment [31].

#### Cuesta landscape

**Fig 9:**
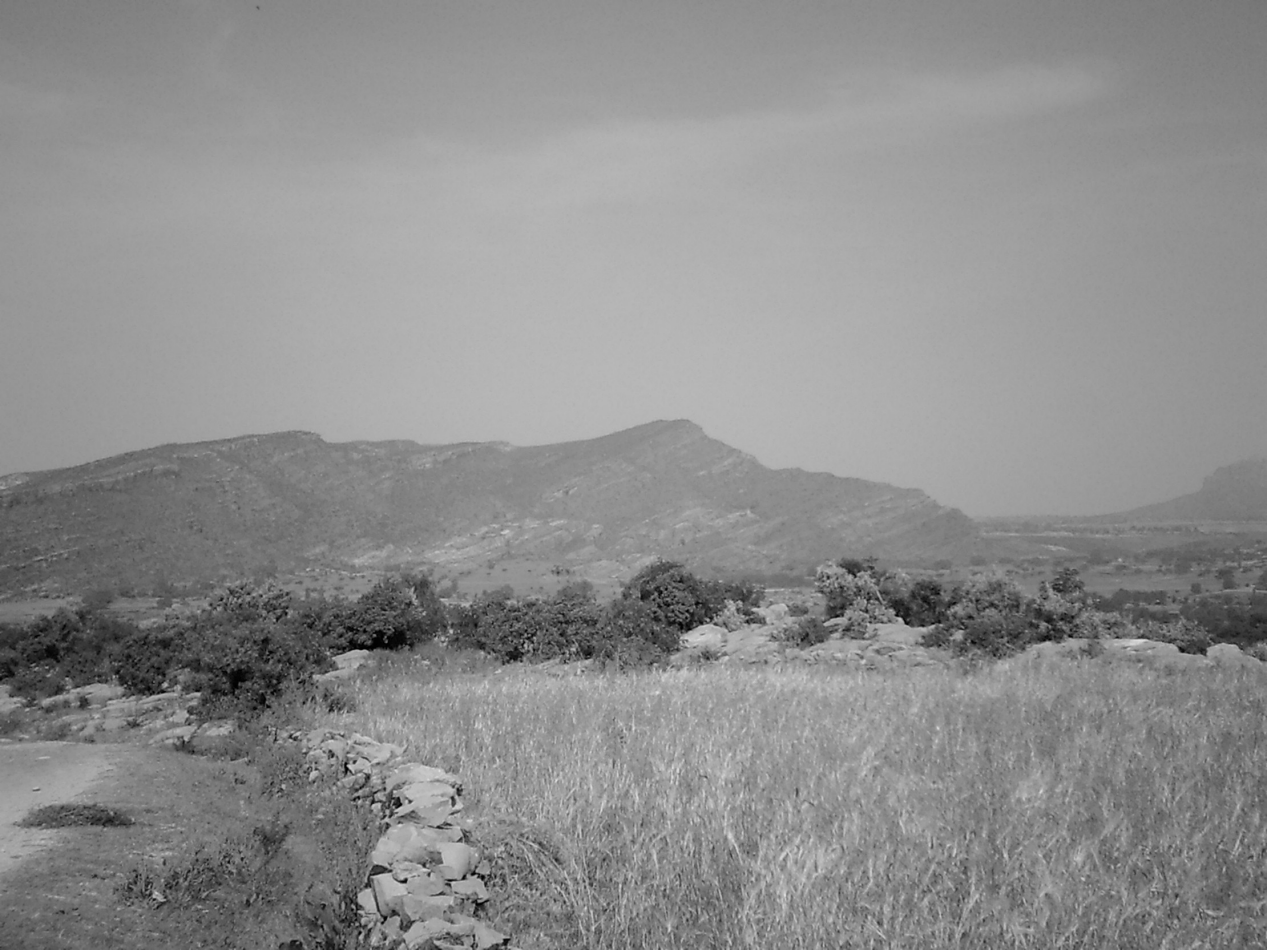
Cuesta landscape between Wuqro to Hawzien.

The cuesta landscape west of Wuqro stretches till Hawzien (outside the Giba catchment). The cuestas, with their back slopes dipping towards the south-southeast, occur both in the Adigrat and the Antalo Formations. The Suluh River cuts consequently through the cuesta fronts. Overall, the slopes are rather steep in this landscape and agriculture is limited to the flatter areas where alluvio-colluvial deposits occur [96], on which Hunting TS [16] described a Cambic Arenosol. The steep slopes, especially on the cuestas, are transformed to exclosure or used as grazing land (Fig 9). Due to these steep slopes, the soils are very shallow and stony. The soils in the colluvial deposits are deeper but still rather shallow with generally a depth around 30 to 40 cm.

#### Soils of the incised Antalo Supersequence plateau

This unit covers the largest area in the catchment; to the north it is confined by the Wuqro fault, in the west it is limited by the Hagere Selam highlands and the steep Amba Aradam Sandstone cliff. The whole area is severely incised due its rapid uplift which started around 25 million years ago and amounted roughly to 2000 metres [97]. The dominant lithologies are limestone, shale and marl (part of the Antalo Supersequence) with many dolerite sills and dykes. When exposed, dolerite forms steep cliffs or flat mountain tops. The dykes are mainly found in the major fault areas, like the Mekelle and Chelekot fault. Because of the high resistance to erosion, these dykes mostly form cliffs when exposed to the surface, Dykes are often associated with tufa dams, when transversal to a river channel. Locally, Adigrat Sandstone outcrops in deeply incised gorges of the larger rivers.

Three main half-grabens are confined by the 3 major faults, from north to south: the Wuqro, the Mekelle and the Chelekot basins (Fig 2). At many places along these fault belts, dolerite has been injected. A more dense and lush vegetation covers the fault escarpments that are lined by dolerite as compared to the limestone cliffs.

**Fig 10:**
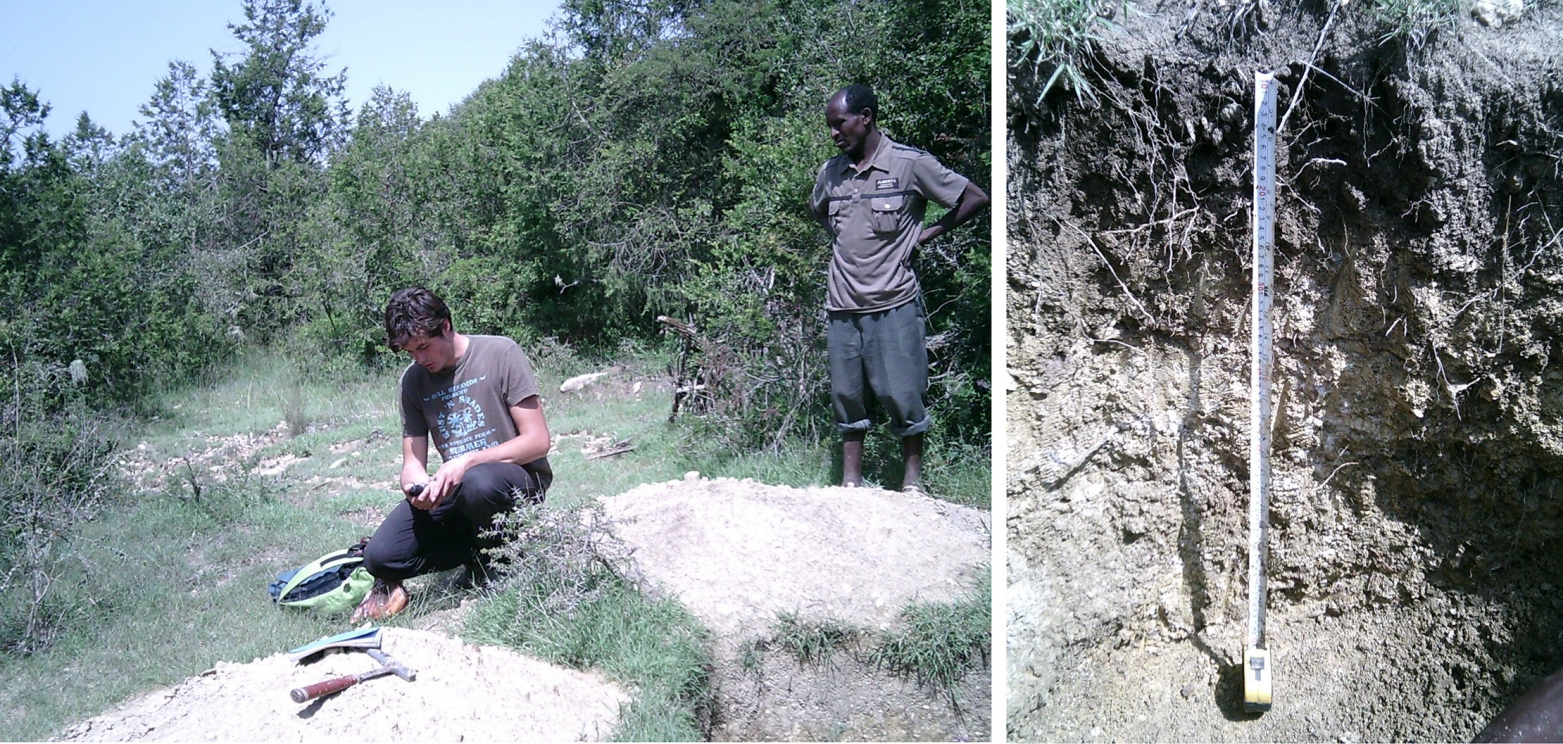
**Location of soil profile pit R in Des’a forest (left) and profile R (Mollic Calcaric Cambisol, right)**.

In the Wuqro basin, limestone and shales each cover half of the area, the terrain morphology is characterised by entrenched river valleys bordered by steep cliffs and separated by undulating to rolling interfluves [49]. On steeper slopes, soil depth is limited and limestone is often outcropping, for instance in Des’a forest (Fig 10). These areas are less suitable for agriculture and mainly used for grazing or transformed to exclosures. On gentle slopes, the soils are rather deep and vertic properties are common especially in the valley bottoms which are often deeply incised. These soils are fertile and almost all of them are under cropland.

The dominant lithology in the Mekelle basin is Agula shale, and dolerite dykes and sills are a common feature. A clear distinction can be made between the western and the eastern part. In the west, the terrain is more hilly and incised. Here, the soils are similar to those of the Wuqro basin. Soil depth is limited on the steeper slopes and not favourable for agriculture; on the gentler slopes soil depth increases and vertic properties are common. The alluvial plains of the larger rivers hold well-developed Fluvisols. The Mekelle basin in the east is rather flat to gently undulating. Vertisol is very common in this semi-graben and provides excellent agricultural lands. The hills that stand out in the landscape consist of dolerite, which is more resistant to erosion compared to the shales.

The Chelekot basin is more incised and more rugged, especially in the area south of the Hagere Selam highlands. South of the Giba River the presence of dolerite resulted in the formation of steep cliffs which demarcate a gentle undulating plateau.

Profile P: Vertic Calcaric Phaeozem. This profile is in Des’a forest (dominated by Juniperus procera and Olea europaea ssp africana [98]) in an open spot in the forest. The parent material is Antalo Limestone. Profile depth is rather limited with bedrock at 70 cm. The A and B horizons have a very good structure and roots are abundant. The A horizon has an organic C content of almost 4% and was classified as a ‘mollic’ horizon. ECEC values (35.7 cmol_c_/kg) are very high in the entire profile and Ca^2+^ (33.4 cmol_c_/kg) is dominant on the exchange complex, in line with the high percentages of CaCO_3_ (10.1%). This soil has some vertic properties (seasonal cracks).

Profiles Q and R: Mollic Calcaric Cambisols. These two profiles are also located in Des’a forest but in more densely vegetated areas (Fig 10). Profile depth is limited to 75 cm with Antalo Limestone as parent material. ECEC values (18.5 cmol_c_/kg) are lower than in profile P but still high. Organic C content (2.4–3.7%) is medium to high in the A horizons which were also classified as mollic horizons. In the topsoil, rock fragments up to 5 cm are common. Very high CaCO_3_ values (18.7%) are found in the entire profiles.

Profile S: Rendzic Leptosol. This profile in Des’a forest is situated in a densely vegetated area. Profile depth is limited (25 cm) due to the outcrop of Antalo Limestone. Like all soils in Des’a forest, this one has also high C values but the highest values are found in the lower layer, below 5 cm (4.1%) which is also darker coloured. The CaCO_3_ content is very high in the topsoil (19.3%) and decreases strongly in the lower layer (4.5%). Both observations tend to indicate that the upper 5 cm may be considered as overwash. ECEC values (34.8 cmol_c_/kg) are again very high and Ca^2+^ (30.4 cmol_c_/kg) is dominant on the exchange complex.

Profile T: Mollic Calcaric Cambisol. The above described forest soils can be considered as the baseline from which most currently occurring soils in the Antalo Supersequence plateau have developed under longstanding human activity, through either truncation of the topsoil or burial by colluvium. Such is for instance the case of profile T along the road between Agula’e and Birki, very close to Birki. The profile is situated on an old river terrace of the Agula’e River at the convex border to the lower lying current terrace. The area is used for cultivation. Profile depth is very limited; at 40 cm depth Antalo Limestone is found.

Chemically this soil is rich with a high ECEC (29.5 cmol_c_/kg), mainly dominated by Ca^2+^, but the high to very high values of exchangeable K^+^ are remarkable (3.69 cmol_c_/kg in the top horizon). As expected by the parent material, the percentages of CaCO_3_ (19.1%) are very high and the pH (7.7) is slightly alkaline. Organic C (1.7%) values are medium, total N (0.18%) values are low and available P (42.8 ppm) is very high. The 25-centimetre thick A horizon has enough organic C (1.9%) to be classified as a mollic horizon.

Similar degraded soils have been described near Mekelle [49]: a Lithic Cambisol and a “Vertic Lithosol”, as well as a Pellic Vertisol. Similarly, two Calcaric Regosol profiles were described in the lower part of the May Zegzeg catchment, which is at the western margin of the Antalo Supersequence plateau, as well as a Phaeozem under forest [43]. In the same area, besides the forest Phaeozems, Calcisol, Calcaric Regosol and Calcaric Cambisol profiles were described on degraded steep slopes [44].

### Soils on Cenozoic volcanics

#### Basalt and dolerite

During the Cenozoic, northern Ethiopia was exposed to very intense magmatic and tectonic activity. It is also in this period that the Ethiopian rift valley formed which caused the uplift of the northern Ethiopian highlands [99]. Two kinds of volcanic depositions can be found in the catchment: flood basalts and dolerite dykes and sills.

The flood basalts were extruded during the Oligocene, and different series of eruptions led to a trap (stepped) landscape [100]. During periods of lesser activity sediment was deposited in lakes that formed in the basalt landscape. Such geological layers are white coloured and consist mainly of very fine grained lacustrine sedimentary rocks which have been silicified [100, 101].

Dolerite is a mafic intrusive rock which has comparable chemical properties as basalt. However, as it did not reach the surface it had more time to crystallise and the crystals are thus bigger compared to basalt. Typical for dolerite is the rounded weathering. Because it is an intrusive rock it is present in the form of sills and dykes. The sills are mainly found in the Antalo Supersequence and may reach a thickness of 80 to 130 m [31]. The dykes are mainly found in the major fault areas, like the Mekelle and Chelekot faults.

#### Basalt-dominated highlands

The basalt-dominated highlands comprise the southern edge of the catchment, the northern Mugulat Mountains (Fig 2) and the central-western part near Hagere Selam. There, the selective erosion of basalt flows led to a trap landscape covering the underlying sedimentary rocks. In the north, the basalt covers the Adigrat Sandstone while around Hagere Selam the Amba Aradam Sandstone is underlying the flood basalts. In both areas, the baked contact of the basalt and the sandstone has (i) increased the resistance against erosion which resulted in a steep cliff, and (ii) induced the local occurrence of perched water tables [102].

Due to the presence of many nutrients in basalt, the soils in these areas are chemically very rich and suitable for cultivation. However, the weathering of basalt may lead to high clay contents, which makes it physically hard to cultivate these soils but if managed properly they are excellent agricultural land. In the Hagere Selam highlands almost all the land is used for cultivation. Especially in the basalt areas, even the steep slopes (up to 30%) are used for cultivation [103].

Hunting TS [49] present a profile description of a Pellic Vertisol some kilometres west of Hagere Selam, most probably in the upper part of the May Zegzeg subcatchment where a detailed soil study was carried out later on [43]. These Vertisols are part of a “red-black soil catena”: from Leptosol over Skeletic Regosol, Cumuli(skeletic) Regosol, Vertic Cambisol to Vertisol [43, 47]. Remnant forest patches also here typically have conserved Phaeozems [44].

Landslide and debris flow deposits are a common feature in this area due to the presence of swelling clays (smectites) derived from basalt, the presence of the lacustrine marl-clayish deposits, the presence of steep slopes and the less permeable baked contact [86, 104]. Basaltic material has been displaced downhill over the sandstone cliff and locally covers sandstone, limestone and marls. A consequence of the occurrence of such ancient landslide and debris flow deposits is the transfer of fertile material to the poorer soils on the sandstone and limestone which provides better conditions for growing crops. In the May Leiba sub-catchment, Van de Wauw [47] noticed that areas covered with basaltic debris were more cultivated than the adjacent fields on limestone and marl of the Antalo Supersequence, and they describe soil profiles in the landslide material that originated from the basalt highlands and covering adjacent limestone: a Vertic Cambisol, a Skeletic Cambisol and a Haplic Vertisol. Therefore, landslides have a significant impact on geomorphology and the spatial pattern of soils in this the landscape and a correct mapping of them is important when making a detailed soil map [47].

### Soils in valley bottoms with Quaternary deposits

**Fig 11:**
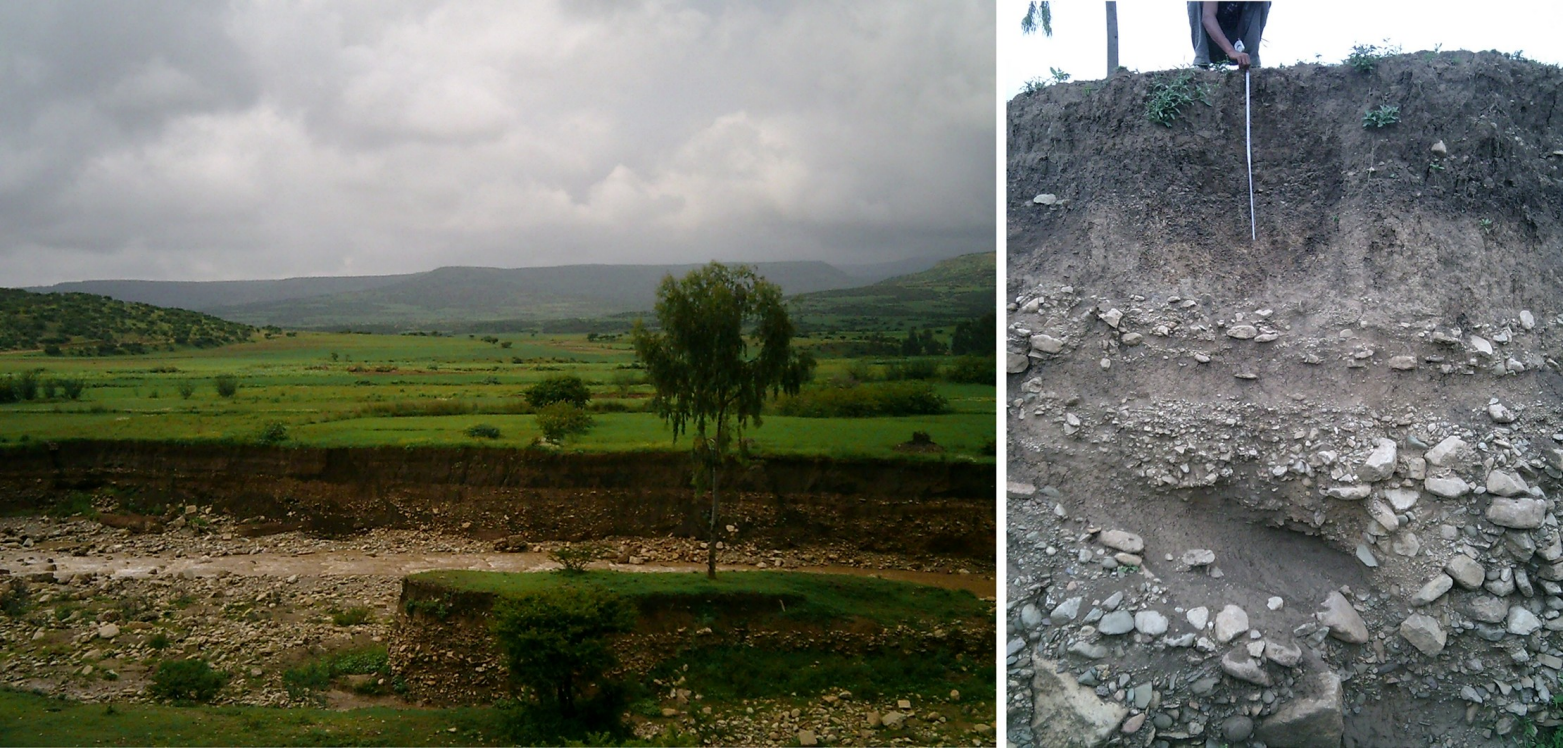
**Location of soil profile O along the Agula’e river (left) and profile O (Mollic Calcaric Fluvisol, right)**.

Two types of recent Quaternary deposits can be found in the catchment: alluvial sediments and carbonate precipitates. The area covered by these deposits comprises only 1% of the catchment, mainly in the floodplains of the Giba River and its tributaries. These deposits may range from well-sorted to poorly-sorted mixtures of clay, silt, sand and pebbles [31] (Fig 11).

Carbonate precipitates include tufa which is rather rare and small in extent, however tufa dams may have a significant impact on landscape evolution. Waterfalls at knickpoints in the longitudinal river channel profile created favourable conditions for CaCO_3_ precipitation due to degassing [105]. Tufa deposition needs more humid conditions than the current climate and is therefore an indication of a wetter period in the past; the dams are generally dated early Holocene [106–108]. At some locations, these tufa deposits could grow up to 10 m high and more, resulting in wide dams, which blocked the course of rivers leading to the formation of small lakes. These tufa dams and their backfill deposits are mainly found on the Mesozoic sedimentary rocks in the central part of the Giba catchment.

Profile K: Haplic Fluvisol. In this profile in a river bank on the Atsbi horst, two layers were distinguished in the fluvic material. The upper layer is of lighter colour and in the lower layer four distinct gravel deposits occur. The area is used as grazing land for the nearby village. The upper layer (C1 horizon) has a lower pH, a lower ECEC value, a lower CaCO_3_ content and a lower organic carbon percentage compared to the underlying stony horizon C2. The darker colour of the lower layer reflects a period of more stability during which organic material could accumulate. The different gravel layers indicate periods of larger stream power that allowed these gravels to be transported.

Profile O: Mollic Calcaric Fluvisol. This profile is in the Agula’e river bank between Agula’e and Birki (Fig 11). The farmland of this profile was not under cultivation, but further away from the river bank almost the entire area is used for cropping. At the bottom of these at least 4 m thick alluvial deposits, large boulders are very abundant, but the deposits become finer towards the top. The uppermost 130 cm was described and three different horizons were distinguished. Chemically and physically the three horizons are quite alike, with high ECEC values (29.9 cmol_c_/kg); CaCO_3_ values (15%) are high to very high and the pH (7.9) indicates slight to moderate alkalinity. The percentages of organic C (1.3%) and BS (100%) are high enough to classify the upper (C1) horizon as a mollic horizon.

## Part III. Soil geography and soil use

### Materials and methods

#### Soil mapping

**Fig 12:**
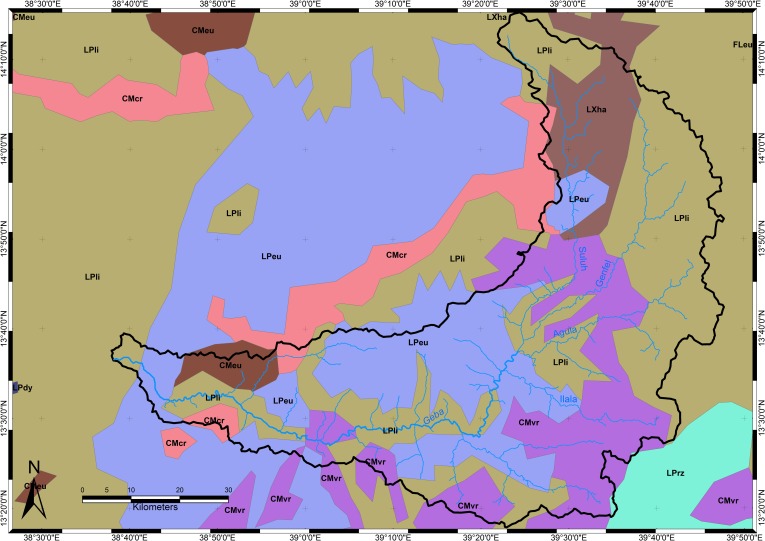
Soil types in Giba catchment, according to the Soil Atlas of Africa [24], which, for Ethiopia, is based on work carried out for FAO in the 1980s [22]. Soil types, by decreasing order of occurrence: LPli = Lithic Leptosol; LPeu = Eutric Leptosol; CMvr = Vertic Cambisol; LXha = Haplic Lixisol; CMcr = Chromic Cambisol; LPrz = Rendzic Leptosol.

Using the description of 141 profile pits, and 1381 soil augerings (Table 1), the soil geography was analysed and mapped. A field-based approach was used in which representative sub-catchments were first mapped in detail (using expert-based delineation of soil polygons [109]), and the obtained recurring land systems and soil groups extrapolated to the larger corresponding geomorphic region. Land systems, conceptualised by CSIRO [110], are areas with specific and unique geomorphic and geological characteristics, and which can be characterised by a particular soil distribution as specified by the soil catena. In line also with the “pédopaysages” approach [111, 112], all available soil information was combined into a comprehensive map at 1:250,000. Given the complex geology and topography of the catchment, this method was preferred over digital or predictive soil mapping [113, 114]). The following digital data were used: the Aster DEM of the catchment, the geological map [31], Landsat images (February 2003), SPOT images (January 2005) and aerial photographs (January 1994). Earlier baseline soil information for the study area was consulted, mainly small-scale maps based on FAO [22] at 1:1,000,000; derived maps include the e-SOTER map [23] and the corresponding sheets in the Soil Atlas of Africa [24, 25] (Fig 12). All these documents follow a different concept from ours, i.e. soil types are overtly generalized and mapped as exclusive polygons.

#### Soil suitability assessment

A soil suitability assessment for agricultural field crops (wheat, barley, teff, lentil, field peas, horse beans and sorghum) was then carried out, in which soil limitations were derived from the soil units following the Soil Fertility Capability System of Sanchez, Palm [115], and the suitability of each soil type was interpreted by the limitation approach [116, 117]. After qualitative assessment, the soil types were grouped into three soil suitability classes (very suitable, moderately suitable and not suitable soils for rainfed cultivation of annual crops), following the principles of the scale-independent FAO Framework for Land Evaluation [118].

S1: Very suitable soils are soils which do not restrict the expected yield much. The yield is thus not negatively affected by their soil properties (both chemically and physically), nor by a steep relief. These are soils that are deep enough, have a good natural fertility and can store sufficient amounts of water.

S2: Moderately suitable soils are soils that restrict the expected yield considerably due to their chemical and/or physical properties or by being located in steep terrain. These can be shallow soils, soils with a limited natural fertility or soils which are not capable of holding and releasing adequate amounts of soil moisture.

N: Not (or marginally) suitable soils are soils that greatly restrict and reduce the expected yields. In our catchment, these are very shallow and stony soils with limited soil depth (generally on steep slopes) or soils with impeded drainage.

### Results

#### Land systems and soil map

Based on soil profile descriptions, augerings, and available soil studies, typical soil units were defined (Table 5), with their main characteristics and classification. The studied region shows a large variability, as can be expected in a mountainous, lithologically contrasted region that has been subject to millennia of land degradation. Within the major geomorphic regions (Fig 3), land systems were defined considering the regional soil-landscape relationships [50]. Soil unit 1 (Leptosol and bare rock) is by far the most dominant soil unit in this very rugged and strongly incised catchment (18.7% coverage). In total, the shallow soils (soil units 1 to 9) cover 39.7% of the area. Soil unit 10 (Vertic Cambisol) is the second most dominant soil unit (13.9% coverage). Together with soil units 11 and 12 (both Vertisol), they cover 24.9% of the catchment. Another dominant soil unit is unit 21 (Eutric Regosol and Cambisol) with a coverage of 9.8%. These are young soils which are mainly found at footslopes.

**Table 5:** Description and WRB [73] classification of the different soil units. The corresponding soil profile description is given between brackets.

	Soil unit	Main soil characteristics	Soil classification
Shallow soils			
1	Undifferentiated, very shallow soils with rock outcrop	Complex of rock outcrops, very stony and very shallow soils	Lithic Leptosol (F), Leptosol, Rock outcrop
2	Undifferentiated, very shallow soils on calcaric material	Complex of rock outcrops, very stony and very shallow soils on calcaric material	Calcaric Leptosol
3	Shallow, stony, dark, silt loamy to loamy soils	Shallow to very shallow soils with a well-structured, dark-coloured surface horizon overlying calcaric material	Rendzic (Calcaric) Leptosol (A,S)
4	Shallow to very shallow, very stony, silt loamy to loamy soils	Shallow to very shallow, somewhat excessively drained soils with very high amounts of stones	(Cumuli)Skeletic Cambisol, Leptic Cambisol (I), Skeletic Regosol
5	Shallow, very stony, silt loamy to loamy soil on calcaric material	Shallow to very shallow, somewhat excessively drained soils with very high amounts of stones on calcaric material	Skeletic Calcaric Cambisol
6	Shallow to moderately deep, dark, silt loamy to loamy soil	Shallow to moderately deep, well drained, dark soils with a good natural fertility	Rendzic Phaeozem, Leptic Phaeozem
7	Shallow to moderately deep silt loamy to loamy soil	Shallow to moderately deep, well drained, brown-yellow soils with a moderate natural fertility	Leptic Luvisol (H)
8	Shallow to very shallow, stony loamy to sandy loam soils	Shallow, stony soils, somewhat excessively drained soils developed on colluvic material	Colluvic Leptosol
9	Shallow sandy to sandy loam soils with indurated layer	Shallow soils with a indurated very hard layer which prevents root penetrating and drainage	Petric Plinthosol
Fine textured			
10	Moderately deep, stony, dark cracking clays	Moderately well or imperfectly drained, moderately deep, very dark greyish brown or black stony clays with good natural fertility	Vertic Cambisol
11	Deep, dark cracking clays on calcaric material with ponded drainage	Moderately well or imperfectly drained, moderately deep to deep, very dark greyish brown to black clays with strong structure and very good natural fertility on calcaric material	Calcaric Vertisol, Calcic Vertisol
12	Deep, dark cracking clays with ponded drainage	Poorly to very poorly drained, deep, dark greyish brown or very dark clays with strong structure and very good natural fertility, temporarily waterlogged during the wet season	Chromic Vertisol (E), Pellic (Calcaric) Vertisol (C)
13	Deep, very hard cracking clays with ponded drainage	Poorly to very poorly drained, deep, very dark clays with very strong structure and very hard upper horizon, good natural fertility, temporarily waterlogged during the wet season	Mazic (Sodic) Vertisol (N)
14	Dark loamy to clay loamy moderately deep soils	Dark, moderately well drained soils with good developed structure and a very good natural fertility	Vertic Phaeozem
15	Deep, dark cracking clays with ponded drainage	Poorly to very poorly drained, deep, very dark clays with strong structure and very good natural fertility, temporarily waterlogged during the wet season	(Pellic) Vertisol
16	Dark, silt loamy to clay loamy moderately deep soils on calcaric material	Dark, moderately well drained soils with good developed structure and a very good natural fertility on calcaric material	Vertic Calcaric Phaeozem (P)
17	Moderately deep, stony, dark cracking clays on calcaric material	Moderately well or imperfectly drained, moderately deep, very dark greyish brown or black stony clays with good natural fertility on calcaric material	Calcaric Vertic Cambisol
18	Shallow, stony, dark clay loamy soils	Moderately well or imperfectly drained, shallow, very dark greyish brown or black stony clays with moderate natural fertility	Epileptic Protovertic Cambisol (B)
Medium to coarse textured			
19	Shallow to moderately deep silt loamy to loamy soils	Moderately well to well drained, shallow to moderately deep, brown, silt loam and loamy soils with a moderate natural fertility	Haplic Cambisol (J)
20	Shallow to moderately deep silt loamy to loamy soils	Moderately well to well drained, shallow to moderately deep, red-brownish, silt loam and loamy soils with a good natural fertility	Chromic Luvisol
21	Shallow, stony silt loamy to sandy loam soils	Well to excessively drained, shallow, stony, dark greyish brown clay loams and sandy loams with weak to moderate structure and moderate fertility	Eutric Regosol, Eutric Cambisol
22	Shallow, stony loam to sandy loam soils on calcaric material	Well to excessively drained, shallow, stony, dark greyish brown clay loams and sandy loams on calcaric material	Calcaric Regosol, Calcaric Cambisol
23	Shallow, dark, stony, silt loamy to loamy soils on calcaric material	Moderately well to well drained, shallow stony soils with a dark well-structured surface layer rich in organic matter with moderate natural fertility	Calcaric Mollic Cambisol (Q, R, T)
24	Sandy clay loams to sands developed on sandy colluvium	Well to excessively drained with weak to moderate structure and moderate natural fertility	Eutric Arenosol, Eutric Regosol, Eutric Cambisol
25	Shallow to moderately deep, stony, brown silt loamy to loamy soils on calcaric material	Moderately well to well drained, shallow to moderately deep, brown, silt loam and loamy soils on calcaric material with a moderate natural fertility	Colluvic Calcic Cambisol (D), Calcic Luvisol
26	Moderately deep, brown silty loamy to loamy soils	Moderately well to well drained, moderately deep, brown, silt loam and loamy soils with a good natural fertility	(Eutric) Luvisol
27	Shallow to very shallow silt loamy to clay loamy soils	Imperfectly to poorly drained, shallow to very shallow, dark soils developed on calcaric material with a moderate natural fertility	Vertic Endoleptic Calcisol
28	Shallow to moderately deep loamy to loamy sandy soils	Moderately well to well drained, moderately deep, (light) brown, loamy to loamy sandy with a moderate to good natural fertility	Chromic Cambisol, Arenic Luvisol, Arenic Lixisol (L)
Stagnic and alluvial soils			
29	Brown, silty loams to loamy sands developed on alluvium	Well drained, deep, dark brown to brown often stratified silty loams to loamy sands with good natural fertility	Fluvisol, Fluvic Cambisol, Mollic Fluvisol (O)
30	Brown to dark, silty clay loams to loamy sands developed on allvium	Well drained to imperfectly drained, deep, brown to gray, dark gray often stratified silty loams to loamy sands with good natural fertility	Vertic Fluvisol, Eutric Fluvisol, Haplic Fluvisol (K, M)
31	Moderately deep clay soils with ponded drainage	Poorly to very poorly drained, moderately to deep, dark brown to dark greyish with strong structure and good natural fertility	Gleyic Vertisol
32	Alluvial clays of flood plains and basins with ponded drainage on calcaric material	Very poorly drained, moderately deep to deep soils with very high water table on calcaric material with moderate to good natural fertility	Calcaric Gleysol
33	Alluvial clays of flood plains and basins with ponded drainage	Very poorly drained, moderately deep to deep soils with very high water table with moderate to good natural fertility	Eutric Gleysol, Gleyic Cambisol
34	Soils with stagnating water due to an abrupt textural change	Poorly to very poorly drained, deep soils with abrupt textural change	Haplic Planosol (G)

#### Soil suitability for agricultural field crops

The different soil units were classified according to their suitability for agricultural field crops [117, 118] (Table 6). Very suitable soils, i.e. soils where the crop yield is not limited by their chemical (fertility) and physical (depth, water holding capacity) soil properties, include, in the study area, soils with vertic properties (Vertisol, Vertic Cambisol), Phaeozem, Luvisol and well-drained Fluvisol. These cover 40.2% of the whole catchment. Moderately suitable soils, i.e. soils that hamper the expected crop yield considerably such as shallow soils, soils with limited natural fertility or other soils which are not capable of holding/releasing large amounts of soil moisture, in the Giba catchment include Leptic Phaeozem and Leptic Luvisol, Rendzic Leptosol, Regosol, Cambisol and shallow soils with vertic properties. They cover 25.1% of the catchment. The soils that are not suitable are very shallow and stony soils (e.g. Leptosol, Skeletic Cambisol), soils with a hard layer which prevents root penetration (Petric Plinthosol) or soils with impeded drainage (e.g. strongly expressed gleyic properties). These cover 34.7% of the entire catchment.

**Table 6:** Classification of the different soil units according to their suitability for agricultural field crops.

Suitability for field crops	Soil units
Very suitable	10, 11, 12, 14, 15, 16, 17, 20, 26, 28, 29, 30
Moderately suitable	3, 6, 7, 13, 18, 19, 21, 22, 23, 24, 25, 27
Not suitable	1, 2, 4, 5, 8, 9, 31, 32, 33, 34

#### Soil distribution and its controlling factors

**Fig 13:**
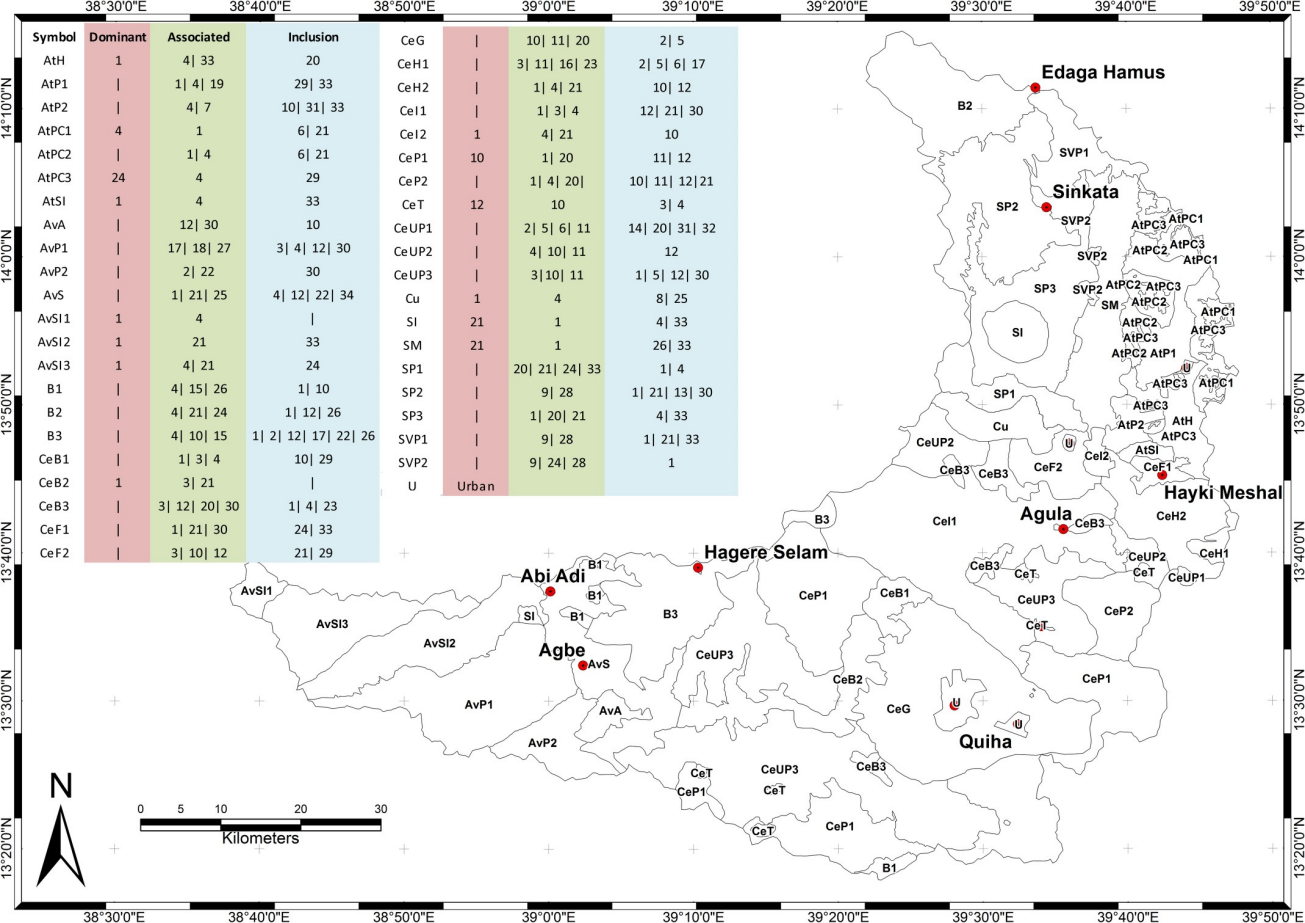
Land systems of Giba catchment with dominant and associated soil types and inclusions. Multiple soil units within a subdivision are separated by |. Soil units are named and characterised in Table 5, and their suitability for agricultural field crops in Table 6. A typical catena for each soil unit has been prepared by Tielens [50]. See the .KMZ file in S2 File, for visualizing the map in Google Earth.

The soil catenas of each land system [50] indicate that topography (relief) and geology (parent material) are the most importing controlling factors that determine the spatial distribution of the different soil units (Fig 13, Table 5, S2 File). Besides these two major factors, vegetation (or land cover) may not be overlooked.

Leptosol and bare rock are found on the steepest slopes (>40%) (soil units 1, 2 and 3). On slopes between 20 and 40% soil depth is still limited but besides Leptosol also shallow and stony Cambisol, Regosol and Phaeozem (if a denser vegetation cover is present) might be found (soil units 4, 5, 6).

On the lower slopes/foot slopes (10–20%), with overal more vegetation cover, more developed but still young soils like Cambisol or Regosol (soil units 4, 5, 21, 22) can be found. The parent material will determine the specific soil unit: a limestone parent material will result in a Calcic or Calcaric qualifier.

**Fig 14:**
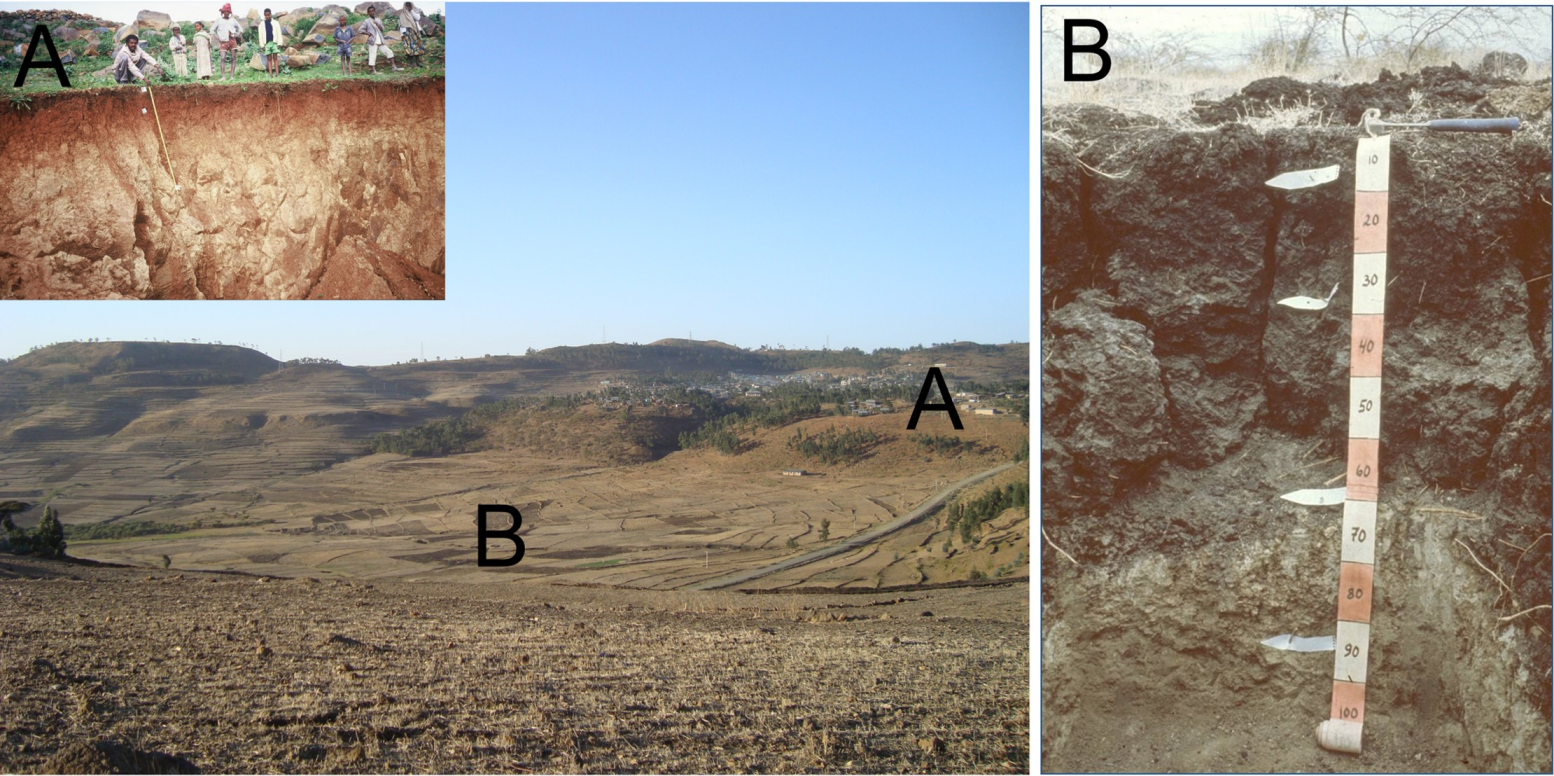
Red-black soil catena near Hagere Selam, after [119]. Luvisol in upper landscape position (A), Skeletic Regosol (stony brownish colluvium) at the foot of the cliff, Vertisol (B) on toeslopes and valley bottom.

On the gentler slopes (2–10%) in the lower situated areas, deeper and the most developed soils can be found. Parent material strongly determines the soil type. Soils with vertic properties (soil units 10, 11, 12, 14, 15, 17, 18) are found on limestones, shales or mafic material. The closer to the valley bottom, the better the vertic properties are developed and the deeper the soil becomes. More reddish soils, Luvisols (soil units 20, 26), can also be found in these areas on more convex areas which results in the typical ‘red-black’ soil catena [85] (Fig 14). If sandstone or Precambrian rocks are the parent material, mainly Cambisol, Regosol and Luvisol/Lixisol are found (soil units 19, 20, 21, 24, 25, 26, 28).

On plateaus, soil depth is also often limited and parent material determines strongly the soil types. On limestone, Rendzic Leptosol (soil unit 3) is very common while on Enticho sandstone Petric Plinthosol (soil unit 9) or shallow soils like Leptosol and shallow, stony Cambisol/Regosol (soil units 1, 4) are found.

In the valley bottoms, fine-textured soils occur with alluvial, stagnic or vertic properties like Fluvisol, Gleysol and Vertisol (soil units 12, 13, 29, 30, 31, 32, 33).

**Fig 15:**
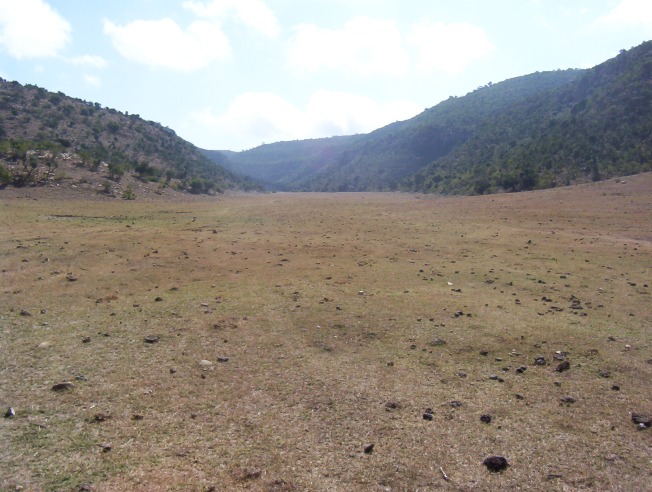
Dambo in Era at the fringe of Des’a forest, after [121]. Gilgai micro-relief is visible indicating the presence of Vertisols with active swell-shrink processes and absence of overwash; on the slopes, under forest (at right) Calcaric Phaeozems, and under degraded forest (at left) Calcaric Cambisols and Rendzic Leptosols.

Areas with sufficient vegetation cover have deeper soils than those without vegetation. If the cover is barely touched by humans, the original soils are at the surface. These are moderately deep to deep Phaeozem on the plateaus and slopes and Vertisol in the lower areas. Such areas have become very scarce, but they can still be found in Des’a forest and the wetter depressions that it holds (“dambos”, *sensu* [120]; Fig 15), as well as in old church forests, where the soils and the vegetation have been protected since a long time [70].

### Discussion

#### Soil suitability for cropping

Despite its strong relief, 40.2% of the Giba catchment is classified as very suitable for agriculture and another 25.1% as moderately suitable. On the other hand, long-standing cropping and soil erosion in this mountainous catchment [3, 4] have led to the presence of large unsuitable areas (26% of the area is covered by bare rock and Leptosols). Furthermore, relatively less erodible clay and sand dominate the soil texture; the high rock fragment contents of topsoils after prolonged tillage and erosion also provide a partial protection against soil erosion [122–124]. Such positive feedback effects have led to a new dynamic equilibrium of the soilscape, not only in relation to tectonic uplift but also in relation to longstanding human impact [125, 126]. In total, 65% would be suitable for crop production. This value is larger than the 42–50% classified as agricultural land in earlier studies [34, 39, 40]. One may however not conclude that the 65% of suitable land indicates that some space is left to be used for cultivation, as some part of these suitable lands are occupied by villages, forest and regenerating semi-natural vegetation. Furthermore, a soil unit might be suitable for agriculture at this moment but it may not be sustainable in the future. For instance, soil units 3, 6 and 7 (7.5% coverage) are moderately suitable for agriculture but these are shallow soils. If used as agricultural land, water and tillage erosion will reduce soil depths even more which may convert them into unsuitable soils in the mid-term. However, the high percentage of suitable soils indicates that the Giba catchment, despite the long-standing soil degradation, still has good agricultural potential.

#### Optimal land use for increased crop productivity

‘Optimal’ land use should minimise soil erosion rates to values less than a tolerable soil loss, i.e. “the maximum level annual amount of soil, which can be removed before the long-term natural soil productivity is adversely affected” [127]. Because soil depth is rather limited, except in the flatter areas towards and in the valley bottoms, this tolerable soil loss should not be larger than the soil formation rates, which, for the semi-arid midlands in northern Ethiopia, were modelled at 6 Mg ha^-1^ year^-1^ [128], but less at higher (due to cold) and lower elevations (due to drought). Measured soil loss rates, at plot, but also at catchment level are well beyond that value [10, 129, 130]. The most efficient way to drastically reduce these erosion rates would be to convert all the cropland that exceeds a critical slope gradient to exclosures [44, 131]. A critical slope gradient of 10% would mean that more than 60% of the whole catchment should be converted into exclosures. This might be a sustainable solution from a long-term view but it is not possible under the current agricultural productivity conditions. At this moment crop yields are even too small for the local population to be self-sufficient–for instance, 66.2% of the population in Ethiopia depended on agriculture for its livelihood in 2018 [132]. Reducing the cropland area cannot be done without increasing specific crop yields.

The use of a wide set of SWCM in the Giba catchment [133] has been proven to drastically reduce the erosion rates [7–9, 134, 135], improve soil quality as well as environment [136, 137] and increase crop yields if they are implemented correctly [103, 138–140]. The participatory approach that is generally implemented in the Tigray Region of Ethiopia combines scientific knowledge and the local knowledge of the farmers, which is likely the most successful approach and is strongly recommended [6, 141].

Deficit or supplementary irrigation, tailor-cut to soil type, is another way to increase crop yields [11]. With the use of crop growth models, like Aquacrop [142, 143], it is possible to develop guidelines for deficit and supplementary irrigation to increase the crop yields. Besides specific information about the crops (e.g. canopy development and transpiration), the climate and the used management practices, Aquacrop requires specific information about the soils in order to calculate the soil water balance [142].

Finally, Integrated Soil Fertility Management (ISFM), i.e. “the application of soil fertility management practices, and the knowledge to adapt these to local conditions, which maximise fertiliser and organic resource use efficiency and crop productivity” [144] would allow to increase the agronomic efficiency, the ratio between the increase in crop yield and the applied nutrients. In the Giba catchment, most of the soils are lacking nitrogen. Incorporating legumes in the rotation system is a simple way to increase the N content [145], which is commonly done by the farmers in the study area [43]. Furthermore, legume-cereal intercropping is especially beneficial in areas with low-input/high-risk environments, such as moisture and nutrient stress in the study area [146]. Despite slow take-off [29], mineral fertilizer has become more popular in recent years. A bottleneck that still needs to be tackled is the insufficient and inappropriate use of manure [29, 147].

## Conclusions

The in-depth study of the Giba catchment soils involved soil profile and augering descriptions, soil type characterisation and comprehension of soil-landscape relations. On the steepest slopes, shallow soils (e.g. Leptosol) and bare rock are found; on the footslopes, more developed but younger soils occur (e.g. Cambisol and Regosol); on the more gentle slopes, the most developed and deeper soils occur but the parent material strongly determines the soil type (e.g. Vertisol, Luvisol, Cambisol); in the valley bottoms, more fine-textured soils with alluvial, stagnic or vertic properties are present; on the plateau, soil depth is often more limited (e.g. Leptosol, Plinthosol).

The geographical distribution of the soil types was determined using land systems, i.e. areas with specific and unique geomorphic and geological characteristics. In this study 41 different and unique land systems were demarcated (Fig 13), each characterised by a particular soil distribution in line with the soil catena. Thirty-four different soil units were distinguished and characterised (Table 1). The most dominant soil units are: unit 1 (Leptosol and bare rock, 19% coverage), unit 10 (Vertic Cambisol, 14% coverage), soil unit 21 (Regosol and Cambisol, 10% coverage), unit 4 (Skeletic/Leptic Cambisol and Regosol, 9% coverage), unit 3 (Rendzic Leptosol, 7% coverage), unit 11 (Calcaric/Calcic Vertisol, 6% coverage), unit 20 (Chromic Luvisol, 6% coverage) and soil unit 12 (Chromic/Pellic Vertisol, 5% coverage). Together these eight soil units cover almost 75% of the catchment.

Topography and parent material are the most important driving factors explaining the soil distribution while vegetation (or land cover) has a less important role, as most parts of the Giba catchment are deforested since many centuries. Without human-induced erosion, the soil distribution would be much more homogenous and topography and parent material would be of lesser importance in controlling the soil distribution. In the new dynamic equilibrium of the soilscape, after major human impacts, younger soils dominate.

Determining the optimal land use for the catchment based on the soil map, strongly depends on the degree to which sustainability is taken into account. Our results show that approximately 65% of the catchment is suitable for agricultural purposes at this moment but not all of these soils can sustain agriculture in the long term. Erosion rates should be reduced to ensure that soil depth will not decrease any further. Yet, the high percentage of suitable soils clearly shows that the Giba catchment has certainly high agricultural potential if correct land management decisions are made.

For most soils, besides water, nitrogen is the most limiting factor for crop growth. Increasing the nitrogen content by e.g. integrated soil fertility management in combination with the ongoing ex-situ and recommended in-situ soil and water conservation measures and irrigated farming will most likely yield the best results.

## Supporting information

S1 File Full profile descriptions of 20 soil profiles. (DOCX)

S2 File Digital map of soil units (.KML file, to be opened in Google Earth). (ZIP)
