# Supplementary material for: Understanding spatial patterns of soils for sustainable agriculture in northern Ethiopia’s tropical mountains
Source: PLoS One. 2019 Oct 22;14(10):e0224041. doi: 10.1371/journal.pone.0224041 (PMC6804989; doi:10.1371/journal.pone.0224041)
Supplement: S1 File — (DOCX) [file pone.0224041.s001.docx]

Understanding spatial patterns of soils for sustainable agriculture in northern Ethiopia’s tropical mountains

Jan Nyssen, Sander Tielens, Tesfamichael Gebreyohannes, Tigist Araya, Kassa Teka, Johan Van de Wauw, Karen Degeyndt, Katrien Descheemaeker, Kassa Amare, Mitiku Haile, Amanuel Zenebe, Neil Munro, Kristine Walraevens, Kindeya Gebrehiwot, Jean Poesen, Amaury Frankl, Alemtsehay Tsegay, Jozef Deckers

S1 Soil profile descriptions

**Profile A**


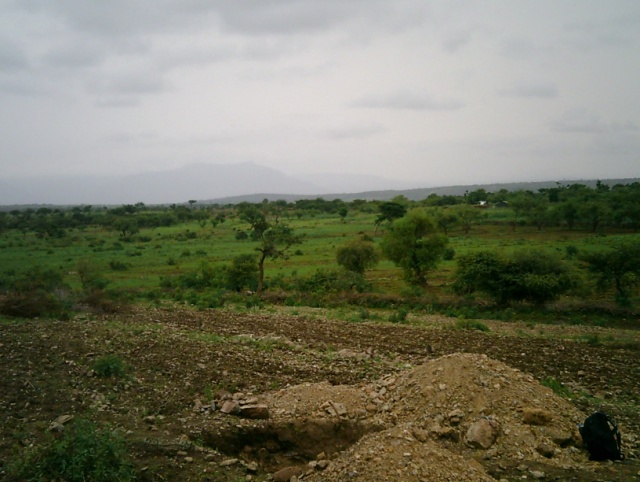


Class: Calcaric Rendzic Leptosol

Date: 16/8/2011

Authors: Sander Tielens & Hagos Mohammedseid

Location: Taget

X 0496769 - Y 1499247 –

Z 1685m


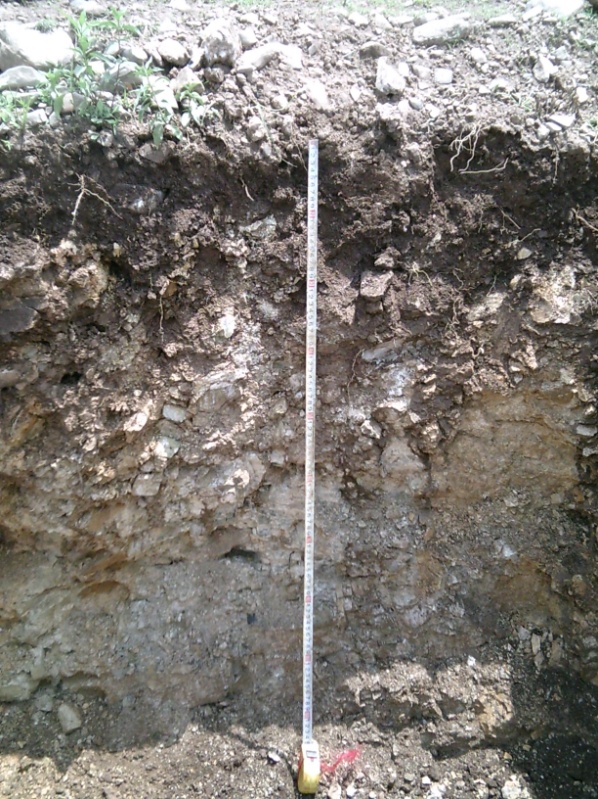


Land use: Cropland

Parent material: Metalimestone

Slope: 14%

Surface stoniness: 47%

Relief: Upper slope


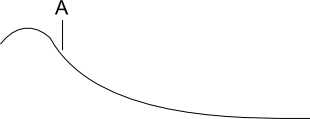


**Profile description**

**Ap** 0-25 cm

Very dark brown 7.5 YR 2.5/3; silt loam; hard when dry; moderate fine crumby; slightly firm when moist; sticky and plastic when wet; strong reaction with HCl; common roots; many very small stones and few small stones.

*Clear sharp boundary to*

**R**

Strong weathered limestone; strong reaction with HCl.

| **Horizon** | **Depth (cm)** | **FC (%)** | **PWP (%)** | **TAW (%)** | **Bulk density (g /cm³)** | **Porosity (%)** |
| --- | --- | --- | --- | --- | --- | --- |
| Ap | 0-25 | 21.69 | 12.67 | 9.02 | 0.98 | 48.50 |

| **Horizon** | **pH H_2_O** | **pH KCl** | **Org. C (%)** | **Tot. N (%)** | **C/N** | **CaCO3 (%)** | **Pav (mg / kg soil)** | **EC (µS/cm)** |
| --- | --- | --- | --- | --- | --- | --- | --- | --- |
| Ap | 7.79 | 7.62 | 1.89 | 0.22 | 8.77 | 19.16 | 15.19 | 190.50 |

| **Horizon** | **Exch. Ca 2+ *** | **Exch. Mg 2+*** | **Exch. K+*** | **Exch. Na+*** | **ECEC*** | **BS (%)** |
| --- | --- | --- | --- | --- | --- | --- |
| Ap | 17.15 | 3.21 | 0.35 | 0.59 | 21.30 | 100.00 |
| *(cmolc/kg) | |  |  |  |  |  |

**Profile B**


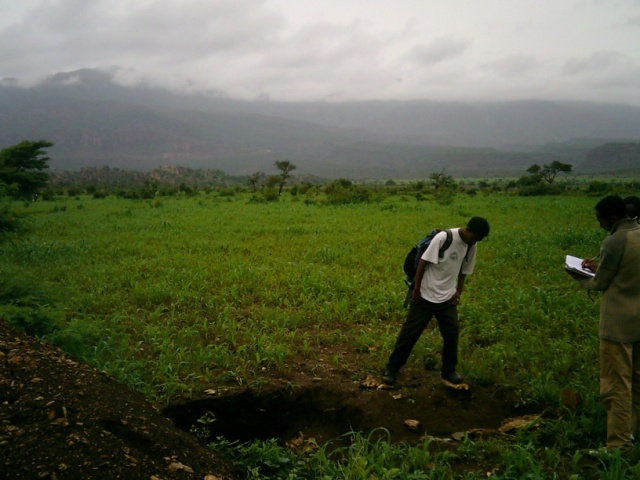


Class: Epileptic Protovertic Cambisol

Date: 16/8/2011

Authors: Sander Tielens & Hagos Mohammedseid

Location: Taget

X 0496991 - Y 1499254 –

Z 1670m


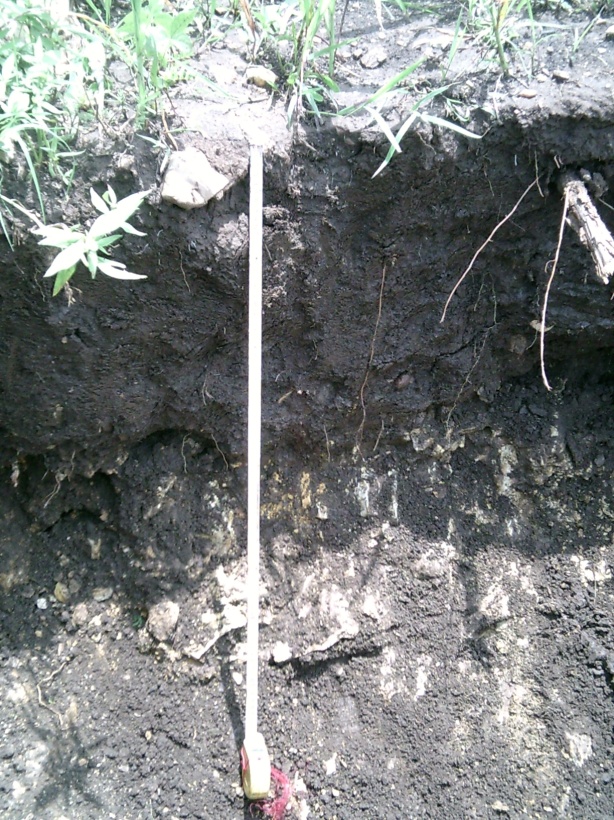
Land use: Cropland (sorghum)

Parent material: Metalimestone

Slope: 0%

Surface stoniness: 14%

Relief: Toe slope


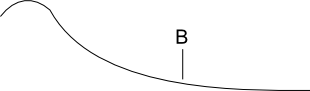


**Profile description**

**Ap** 0-20 cm

Dark brown 7.5 YR 2/1; clay loam; hard when dry; strong medium granular; friable when moist; very sticky and plastic when wet; slight reaction with HCl; common roots; few very small stones.

*Gradual smooth boundary to*

**B** 20-45 cm

Black 10 YR 2/1; light clay; hard/firm when dry; strong medium granular; firm when moist, very sticky and plastic when wet; moderate reaction with HCl; patchy slickensides; few roots

*Clear sharp boundary to*

**R**

Strong weathered limestone; strong reaction with HCl

| **Horizon** | **Depth (cm)** | **FC (%)** | **PWP (%)** | **TAW (%)** | **Bulk density** | **Porosity (%)** |
| --- | --- | --- | --- | --- | --- | --- |
| Ap | 0-20 | 25.87 | 23.00 | 2.87 | 1.06 | 42.26 |
| B | 20-45 | 29.47 | 32.28 |  | 1.37 | 40.17 |

| **Horizon** | **pH H_2_O** | **pH KCl** | **Org. C (%)** | **Tot. N (%)** | **C/N** | **CaCO3 (%)** | **Pav (mg / kg soil)** | **EC (µS/cm)** |
| --- | --- | --- | --- | --- | --- | --- | --- | --- |
| Ap | 7.28 | 6.86 | 1.52 | 0.09 | 17.27 | 2.89 | 8.57 | 134.90 |
| B | 7.15 | 6.62 | 0.89 | 0.06 | 15.57 | 2.99 |  | 90.50 |

| **Horizon** | **Exch. Ca 2+ *** | **Exch. Mg 2+*** | **Exch. K+*** | **Exch. Na+*** | **ECEC*** | **BS (%)** |
| --- | --- | --- | --- | --- | --- | --- |
| Ap | 29.05 | 5.46 | 0.21 | 0.21 | 34.93 | 100.00 |
| B | 28.87 | 6.58 | 0.30 | 0.25 | 36.00 | 100.00 |
| *(cmolc/kg) |  |  |  |  |  |  |

**Profile C**


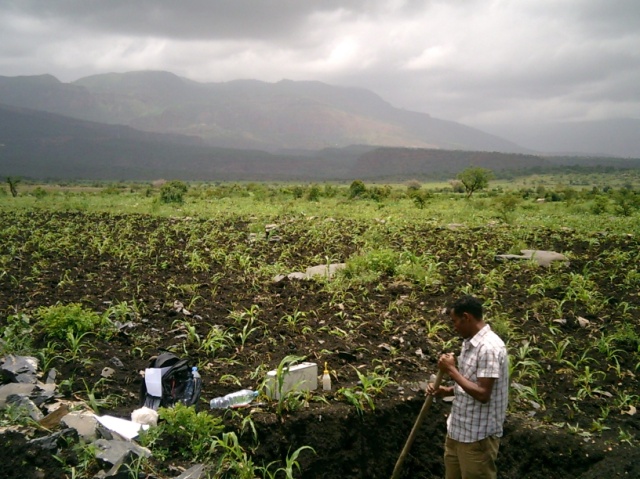


Class: Pellic Vertisol

Date: 16/8/2011

Authors: Sander Tielens & Hagos Mohammedseid

Location: Taget

X 0497470 - Y 1499260 –

Z 1660m


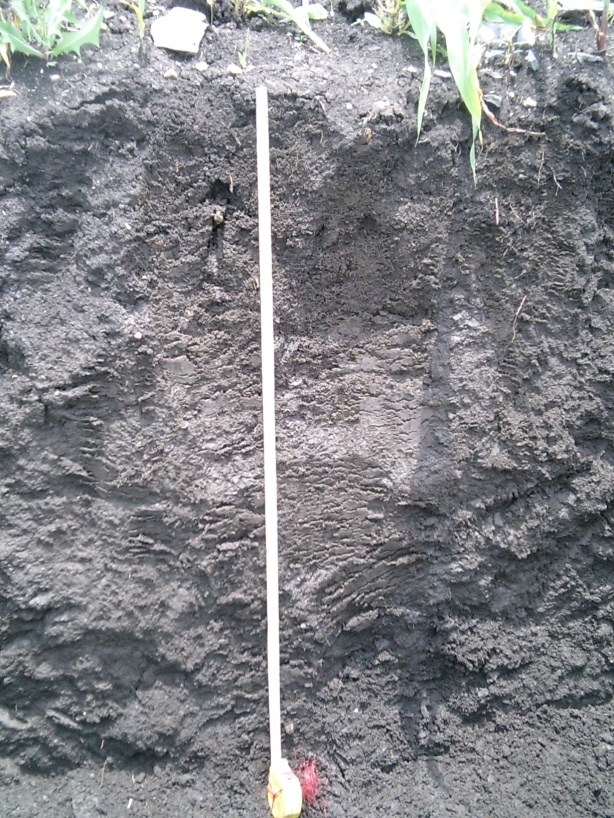
Land use: Cropland (sorghum)

Parent material: Metalimestone

Slope: 2%

Surface stoniness: 16%

Relief: Valley bottom


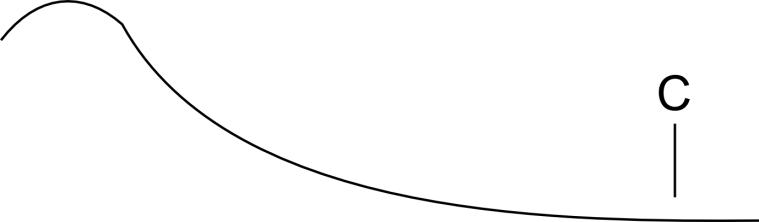


**Profile description**

**Ap** 0-30 cm

Very dark gray 7.5 YR 3/1; heavy clay (decantation: silty clay); hard when dry; strong fine granular; firm when moist; very sticky and plastic when wet; no reaction with HCl; common fine roots.

*Gradual smooth boundary to*

**B** 30-90 cm

Black 10 YR 2/1; heavy clay (decantation: clay); very hard when dry; strong medium angular blocky; firm when moist; very sticky and plastic when wet; no reaction with HCl; small lime stone rocks; clearly visible slickensides and gilgai; few roots.

| **Horizon** | **Depth (cm)** | **FC (%)** | **PWP (%)** | **TAW (%)** | **Bulk density** | **Porosity (%)** |
| --- | --- | --- | --- | --- | --- | --- |
| Ap | 0-30 | 33.43 | 30.21 | 3.22 | 1.02 | 53.56 |
| B | 30-90 | 36.87 | 37.87 |  | 1.30 | 41.64 |

| **Horizon** | **pH H_2_O** | **pH KCl** | **Org. C (%)** | **Tot. N (%)** | **C/N** | **CaCO3 (%)** | **Pav (mg / kg soil)** | **EC (µS/cm)** |
| --- | --- | --- | --- | --- | --- | --- | --- | --- |
| Ap | 7.50 | 6.76 | 1.37 | 0.08 | 16.12 | 4.77 | 9.58 | 167.20 |
| B | 7.61 | 6.85 | 1.39 | 0.10 | 14.23 | 4.61 |  | 187.40 |

| **Horizon** | **Exch. Ca 2+ *** | **Exch. Mg 2+*** | **Exch. K+*** | **Exch. Na+*** | **ECEC*** | **BS (%)** |
| --- | --- | --- | --- | --- | --- | --- |
| Ap | 31.11 | 6.08 | 0.65 | 0.70 | 38.55 | 100.00 |
| B | 30.12 | 7.60 | 0.42 | 0.77 | 38.91 | 100.00 |
| *(cmolc/kg) | | | | |  |  |

**Profile D**


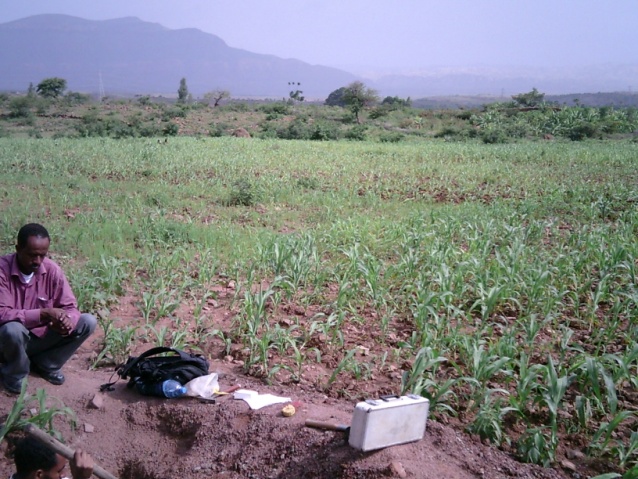


Class: Colluvic Calcic Luvisol

Date: 16/8/2011

Authors: Sander Tielens & Hagos Mohammedseid

Location: Agbe

X 0505437 - Y 1495835–

Z 1542m


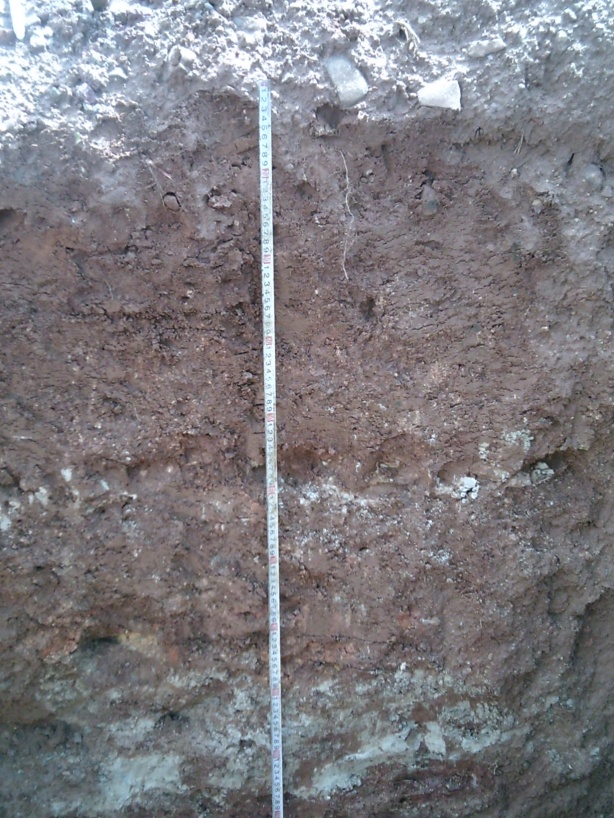
Land use: Cropland (mais)

Parent material: Adigrat sandstone (colluviums)

Slope: 5%

Surface stoniness: 30%

Relief: Foot slope


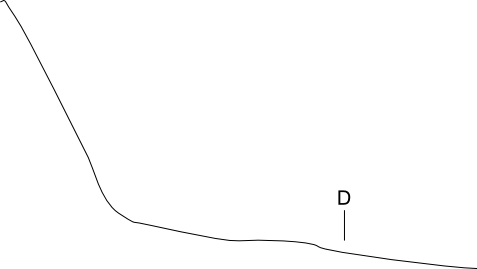


**Profile description**

**Ap** 0-25 cm

Dark red brown 5 YR 3/3; loam; very hard when dry; moderate fine granular; firm when moist; slightly sticky and plastic when wet; strong reaction with HCl; few fine roots; few very small stones.

*Gradual smooth boundary to*

**B** 25-75 cm

Reddish-brown 5YR 4/4; silt loam; hard when dry; moderate medium granular; firm when moist; sticky and plastic when wet; strong reaction with HCl; many hard lime stone concretions (<0.5 mm); very few roots.

*Clear sharp boundary to*

**C**

Strong weathered Adigrat sandstone

| **Horizon** | **Depth (cm)** | **FC (%)** | **PWP (%)** | **TAW (%)** | **Bulk density** | **Porosity (%)** |
| --- | --- | --- | --- | --- | --- | --- |
| Ap | 0-25 | 27.14 | 13.62 | 13.52 | 1.58 | 33.39 |
| B | 25-75 | 25.90 | 26.81 |  | 1.62 | 34.18 |

| **Horizon** | **pH H_2_O** | **pH KCl** | **Org. C (%)** | **Tot. N (%)** | **C/N** | **CaCO3 (%)** | **Pav (mg / kg soil)** | **EC (µS/cm)** |
| --- | --- | --- | --- | --- | --- | --- | --- | --- |
| Ap | 8.02 | 7.75 | 0.89 | 0.06 | 13.72 | 9.57 | 14.61 | 152.60 |
| B | 8.01 | 7.48 | 0.52 | 0.08 | 6.58 | 17.39 |  | 187.10 |
| C | 7.86 | 7.20 | 0.80 | 0.07 | 11.28 | 9.12 |  | 120.30 |

| **Horizon** | **Exch. Ca 2+ *** | **Exch. Mg 2+*** | **Exch. K+*** | **Exch. Na+*** | **ECEC*** | **BS (%)** |
| --- | --- | --- | --- | --- | --- | --- |
| Ap | 13.28 | 0.97 | 1.09 | 0.22 | 15.83 | 98.26 |
| B | 13.91 | 4.53 | 1.55 | 0.73 | 20.72 | 100.00 |
| C | 33.20 | 1.16 | 0.59 | 0.74 | 35.69 | 100.00 |
| *(cmolc/kg) |  |  |  |  |  |  |

**Profile E**


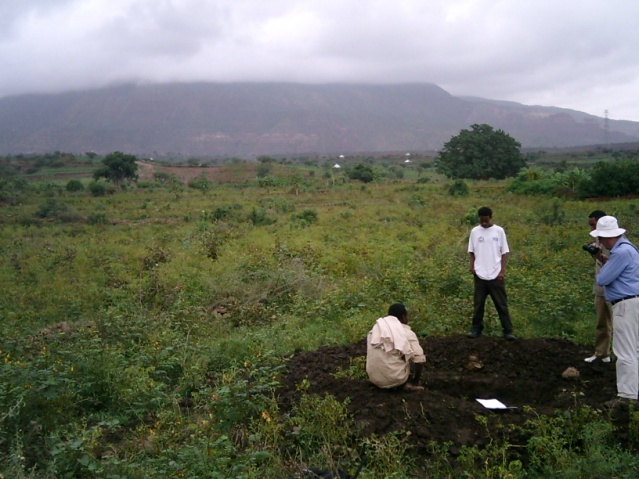


Class: Chromic Vertisol

Date: 16/8/2011

Authors: Sander Tielens & Hagos Mohammedseid

Location: Agbe

X 0505184 - Y 1495243 –

Z 1513m


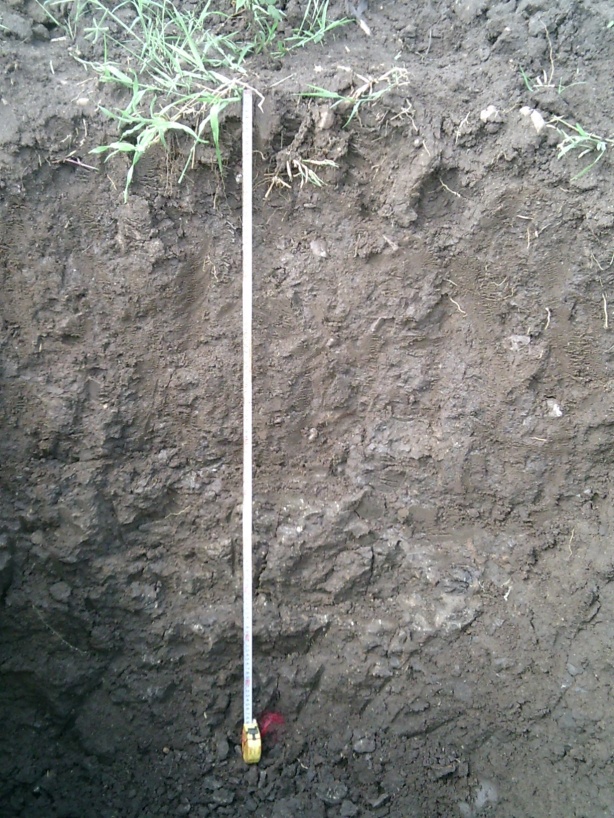
Land use: Cropland (cotton and pepper)

Parent material: Basalt

Slope: 1.5%

Surface stoniness: 24%

Relief: Valley bottom


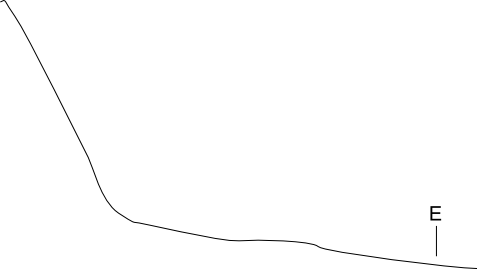


**Profile description**

**Ap** 0-25 cm

Dark reddish brown 5 YR 3/2; heavy clay (decantation: clay loam); hard when dry; moderate fine granular; firm when moist; very sticky and plastic when moist; moderate reaction with HCl; many roots; few very small stones.

*Gradual smooth boundary to*

**B** 25-120 cm

Dark reddish brown 5 YR 2/2; heavy clay (decantation: clay); very hard when dry; strong fine angular blocky; firm when moist; very sticky and plastic when wet; strong reaction with HCl; very clear slickensides; very few roots; few very small stones.

| **Horizon** | **Depth (cm)** | **FC (%)** | **PWP (%)** | **TAW (%)** | **Bulk density** | **Porosity (%)** |
| --- | --- | --- | --- | --- | --- | --- |
| Ap | 0-25 | 26.84 | 22.62 | 4.22 | 1.36 | 39.96 |
| B | 25-120 | 27.11 | 25.78 | 1.33 | 1.53 | 34.84 |

| **Horizon** | **pH H_2_O** | **pH KCl** | **Org. C (%)** | **Tot. N (%)** | **C/N** | **CaCO3 (%)** | **Pav (mg / kg soil)** | **EC (µS/cm)** |
| --- | --- | --- | --- | --- | --- | --- | --- | --- |
| Ap | 7.81 | 7.28 | 0.88 | 0.09 | 10.19 | 3.26 | 12.03 | 145.40 |
| B | 7.89 | 7.26 | 0.90 | 0.07 | 13.26 | 3.17 |  | 158.40 |

| **Horizon** | **Exch. Ca 2+ *** | **Exch. Mg 2+*** | **Exch. K+*** | **Exch. Na+*** | **ECEC*** | **BS (%)** |
| --- | --- | --- | --- | --- | --- | --- |
| Ap | 24.59 | 5.35 | 0.81 | 0.46 | 31.20 | 100.00 |
| B | 22.81 | 7.41 | 0.45 | 0.60 | 31.28 | 100.00 |
| *(cmolc/kg) | | | | |  |  |

**Profile F**


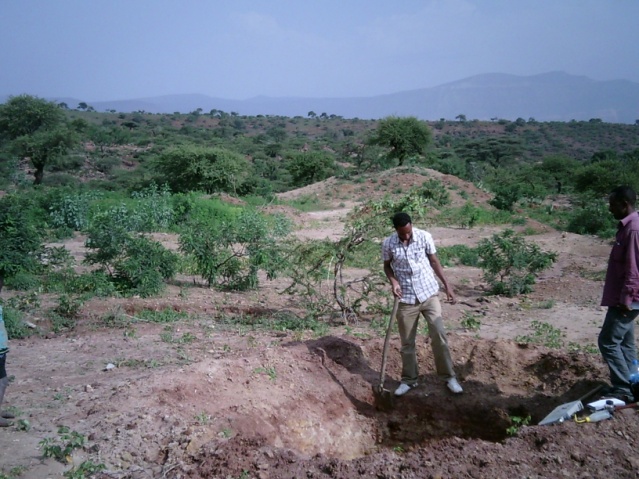


Class: Lithic Leptosol

Date: 16/8/2011

Authors: Sander Tielens & Hagos Mohammedseid

Location: Agbe

X 0505986 - Y 1496951 –

Z 1601m


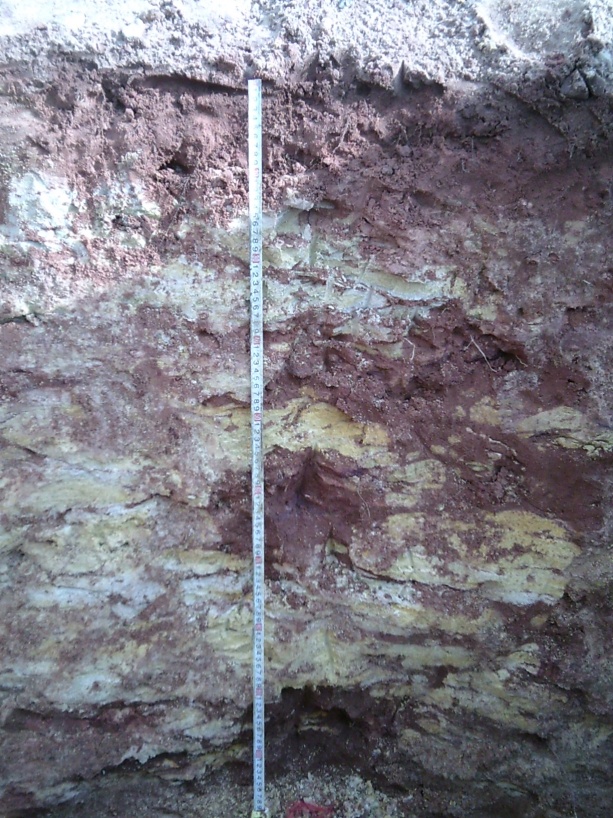


Land use: Rangeland

Parent material: Adigrat sandstone

Slope: 8%

Surface stoniness: 14%

Relief: Toe slope


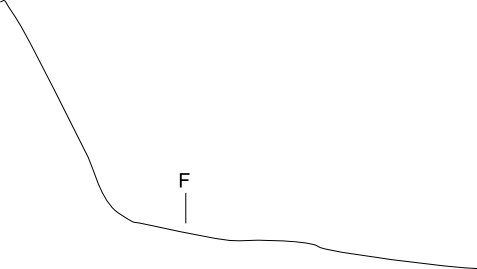


**Profile description**

**Ah** 0-10 cm

Red 10R 4/6; sand; hard/firm when dry; moderate fine crumby; very firm when moist; slightly sticky and plastic when wet; strong reaction with HCl; few roots.

*Clear sharp boundary to*

**C**

Yellow 2.5 Y 7/8; loamy sand; hard/friable when dry; moderate fine granular; slightly firm when moist; slightly sticky and plastic when wet; strong reaction with HCl; few roots; strong weathered banded Adigrat sandstone.

| **Horizon** | **Depth (cm)** | **FC (%)** | **PWP (%)** | **TAW (%)** | **Bulk density** | **Porosity (%)** |
| --- | --- | --- | --- | --- | --- | --- |
| Ap | 0-10 | 8.04 | 4.25 | 3.79 | 1.74 | 32.42 |
| C | >10 | 1.21 | 3.00 |  | 1.58 | 32.38 |

| **Horizon** | **pH H_2_O** | **pH KCl** | **Org. C (%)** | **Tot. N (%)** | **C/N** | **CaCO3 (%)** | **Pav (mg / kg soil)** | **EC (µS/cm)** |
| --- | --- | --- | --- | --- | --- | --- | --- | --- |
| Ap | 8.63 | 8.59 | 0.25 | 0.02 | 13.47 | 1.78 | 32.38 | 79.60 |
| C | 8.83 | 8.96 | 0.03 | 0.01 | 4.11 | 2.19 |  | 129.60 |

| **Horizon** | **Exch. Ca 2+ *** | **Exch. Mg 2+*** | **Exch. K+*** | **Exch. Na+*** | **ECEC*** | **BS (%)** |
| --- | --- | --- | --- | --- | --- | --- |
| Ap | 8.43 | 0.23 | 0.40 | 0.06 | 9.11 | 100.00 |
| C | 3.78 | 6.30 | 0.32 | 0.15 | 10.56 | 100.00 |
| *(cmolc/kg) |  |  |  |  |  |  |


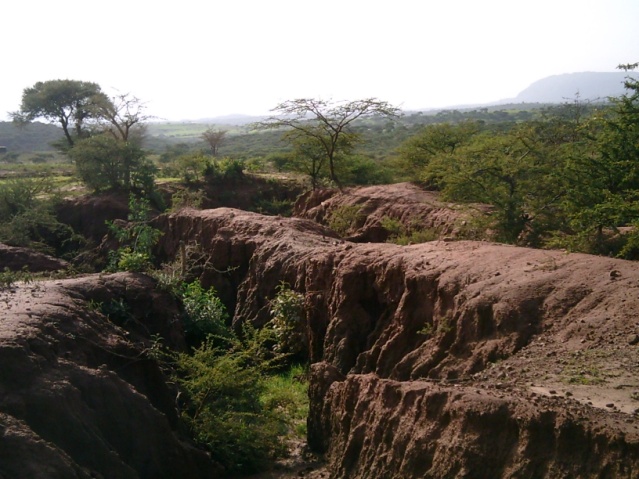
**Profile G**

Class: Haplic Planosol

Date: 16/8/2011

Authors: Sander Tielens & Hagos Mohammedseid

Location: Abiy Addi

X 0499580 - Y 1504386 –

Z 1813m


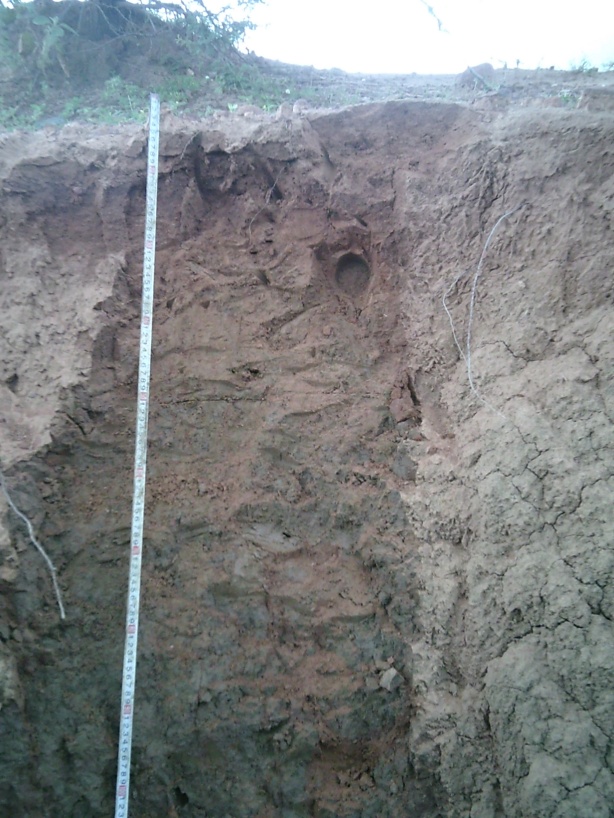


Land use: Rangeland

Parent material: Adigrat Sandstone

Slope: 8%

Surface stoniness: 12%

Relief: Foot slope


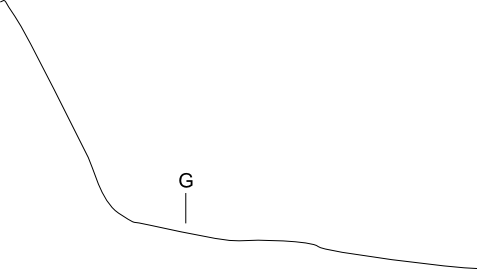


**Profile description**

**AC** 0-40 cm

Yellowish-red 5YR 4/6; loamy sand; friable when dry; weak very fine crumby; friable when moist; sticky and plastic when wet; slight reaction with HCl; very few roots.

*Clear sharp boundary to*

**C**

Olive brown 2.5 Y 4/3; light clay (decantation: clay); very hard when dry; strong fine angular; firm when moist; very sticky and plastic when wet; no reaction with HCl; no roots.

| **Horizon** | **Depth (cm)** | **FC (%)** | **PWP (%)** | **TAW (%)** | **Bulk density** | **Porosity (%)** |
| --- | --- | --- | --- | --- | --- | --- |
| AC | 0-40 | 17.27 | 9.28 | 7.99 | 1.72 | 29.41 |
| C | >40 | 25.74 | 27.35 |  | 1.67 | 30.08 |

| **Horizon** | **pH H_2_O** | **pH KCl** | **Org. C (%)** | **Tot. N (%)** | **C/N** | **CaCO3 (%)** | **Pav (mg / kg soil)** | **EC (µS/cm)** |
| --- | --- | --- | --- | --- | --- | --- | --- | --- |
| AC | 7.74 | 6.97 | 0.14 | 0.02 | 5.65 | 1.29 | 10.83 | 56.70 |
| C | 7.65 | 7.22 | 0.21 | 0.03 | 7.50 | 0.61 |  | 125.30 |

| **Horizon** | **Exch. Ca 2+ *** | **Exch. Mg 2+*** | **Exch. K+*** | **Exch. Na+*** | **ECEC*** | **BS (%)** |
| --- | --- | --- | --- | --- | --- | --- |
| AC | 3.94 | 0.29 | 0.39 | 0.03 | 6.49 | 71.47 |
| C | 10.53 | 2.61 | 0.98 | 0.25 | 15.90 | 90.42 |
| *(cmolc/kg) | | | | |  |  |

**Profile H**


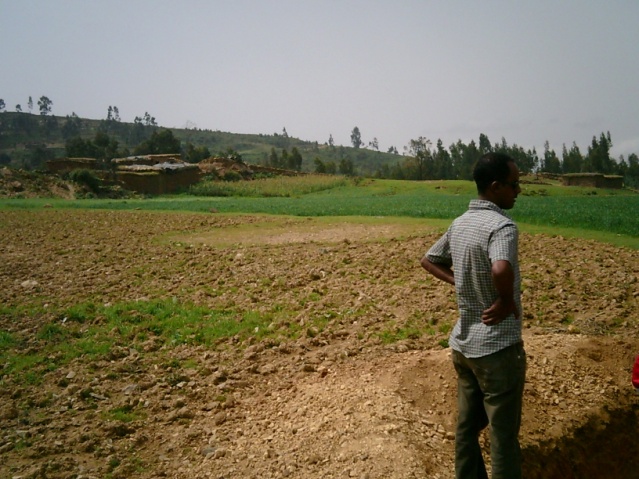


Class: Leptic Luvisol

Date: 26/8/2011

Authors: Sander Tielens & Hagos Mohammedseid

Location: Atsbi

X 0574518 - Y 1527103 –

Z 2609m


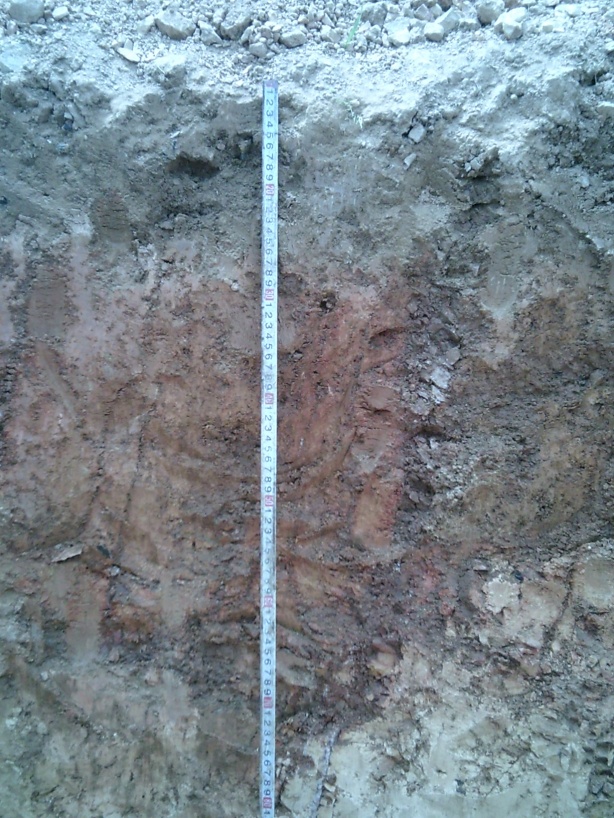
Land use: Cropland

Parent material: Metasediment

Slope: 0%

Surface stoniness: 28%

Relief: Crest, plateau


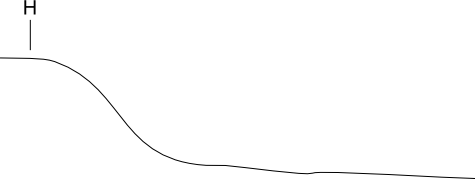


**Profile description**

**Ap** 0-15 cm

Strong brown 7.5 YR 4/6; silt loam; very firm when dry; strong fine granular; firm when moist; slightly sticky and plastic when wet; moderate reaction with HCl; common roots

*Clear sharp boundary to*

**B** 15-60 cm

Reddish yellow 7.5 YR 6/8; silt loam; very firm when dry; strong fine granular; firm when moist; slightly sticky and plastic when wet; strong reaction with HCl; very few fine roots.

*Clear wavy boundary to*

**C**

Olive yellow 2.5 Y 6/8; loam; hard when dry; moderate fine granular; very firm when moist, slightly sticky and plastic when wet; strong reaction with HCl; no roots.

| **Horizon** | **Depth (cm)** | **FC (%)** | **PWP (%)** | **TAW (%)** | **Bulk density** | **Porosity (%)** |
| --- | --- | --- | --- | --- | --- | --- |
| Ap | 0-15 | 31.85 | 18.49 | 13.36 | 1.26 | 50.47 |
| B | 15-60 | 34.71 | 19.59 | 15.12 | 0.94 | 53.57 |
| C | >60 |  |  |  |  |  |

| **Horizon** | **pH H_2_O** | **pH KCl** | **Org. C (%)** | **Tot. N (%)** | **C/N** | **CaCO3 (%)** | **Pav (mg / kg soil)** | **EC (µS/cm)** |
| --- | --- | --- | --- | --- | --- | --- | --- | --- |
| Ap | 7.48 | 6.73 | 0.76 | 0.09 | 8.79 | 2.54 | 112.68 | 139.90 |
| B | 7.20 | 5.33 | 0.86 | 0.07 | 12.71 | 4.55 |  | 92.40 |
| C | 7.58 | 5.56 | 0.17 | 0.03 | 6.35 | 4.51 |  | 116.40 |

| **Horizon** | **Exch. Ca 2+ *** | **Exch. Mg 2+*** | **Exch. K+*** | **Exch. Na+*** | **ECEC*** | **BS (%)** |
| --- | --- | --- | --- | --- | --- | --- |
| Ap | 18.43 | 5.10 | 0.48 | 0.17 | 24.92 | 97.02 |
| B | 24.40 | 13.24 | 0.13 | 0.22 | 37.99 | 100.00 |
| C | 23.14 | 13.96 | 0.08 | 0.19 | 37.37 | 100.00 |
| *(cmolc/kg) |  |  |  |  |  |  |

**Profile I**


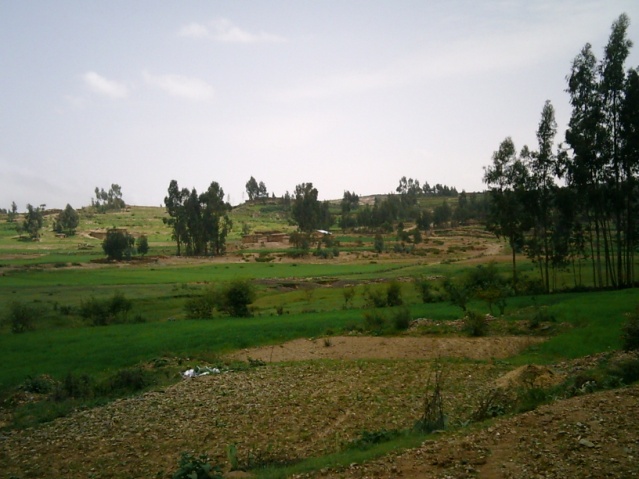


Class: Leptic Cambisol

Date: 26/8/2011

Authors: Sander Tielens & Hagos Mohammedseid

Location: Atsbi

X 0574378 - Y 1527280 –

Z 2581m


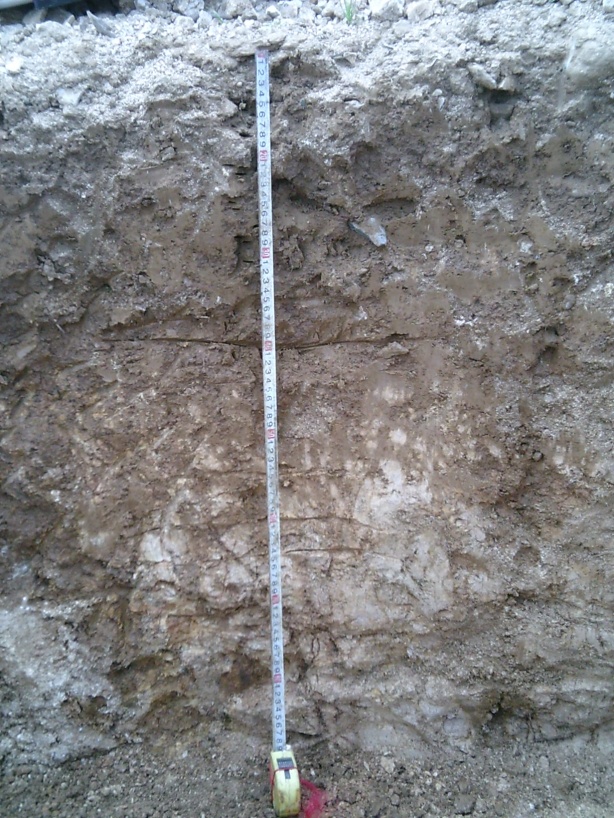


Land use: Cropland

Parent material: Metasediment

Slope: 10%

Surface stoniness: 70%

Relief: Foot slope


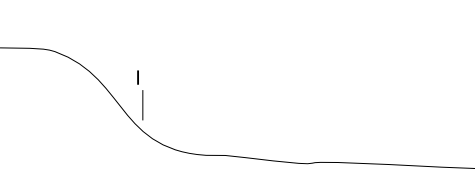


**Profile description**

**Ap** 0-25 cm

Dark yellowish brown 10 YR 4/6; loam; hard when dry; moderate very fine granular; firm when moist; sticky and plastic when wet; slight reaction with HCl; few roots; few very small stones.

*Gradual smooth boundary to*

**B** 25-60 cm

Yellowish brown 10 YR 5/6; loam; friable when dry; moderate fine granular; friable when moist; sticky and plastic when wet; very slight reaction with HCl; few roots; few very small stones.

*Clear sharp boundary to*

**C**

Brownish yellow 10 YR 6/6; loam; friable when dry; moderate fine granular; firm when moist; slightly sticky and plastic when wet; very slight reaction with HCl; no roots.

| **Horizon** | **Depth (cm)** | **FC (%)** | **PWP (%)** | **TAW (%)** | **Bulk density** | **Porosity (%)** |
| --- | --- | --- | --- | --- | --- | --- |
| Ap | 0-25 | 28.60 | 15.91 | 12.69 | 1.36 | 46.65 |
| B | 25-60 | 24.03 | 12.29 | 11.74 | 1.47 | 43.81 |
| C | >60 |  |  |  |  |  |

| **Horizon** | **pH H_2_O** | **pH KCl** | **Org. C (%)** | **Tot. N (%)** | **C/N** | **CaCO3 (%)** | **Pav (mg / kg soil)** | **EC (µS/cm)** |
| --- | --- | --- | --- | --- | --- | --- | --- | --- |
| Ap | 6.90 | 5.61 | 0.52 | 0.05 | 10.95 | 1.40 | 12.81 | 57.60 |
| B | 6.62 | 5.37 | 0.39 | 0.04 | 10.44 | 1.04 |  | 132.60 |
| C | 6.79 | 5.81 | 0.14 | 0.02 | 7.25 | 0.75 |  | 44.10 |

| **Horizon** | **Exch. Ca 2+ *** | **Exch. Mg 2+*** | **Exch. K+*** | **Exch. Na+*** | **ECEC*** | **BS (%)** |
| --- | --- | --- | --- | --- | --- | --- |
| Ap | 8.43 | 3.58 | 0.13 | 0.18 | 15.03 | 81.95 |
| B | 4.59 | 3.21 | 0.14 | 0.12 | 10.22 | 78.97 |
| C | 2.91 | 1.99 | 0.16 | 0.16 | 7.90 | 65.95 |
| *(cmolc/kg) |  |  |  |  |  |  |

**Profile J**


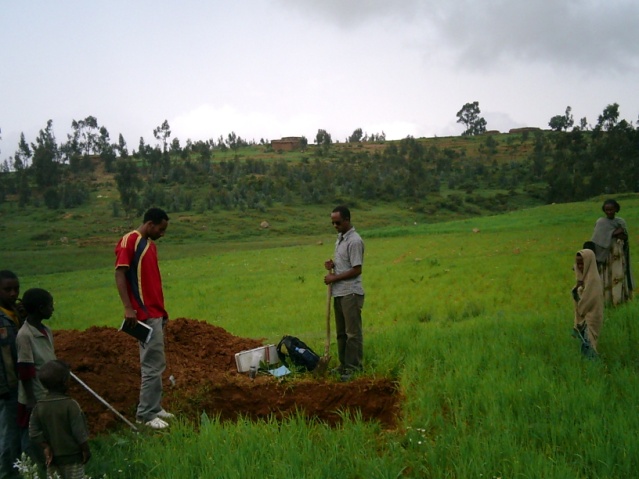


Class: Haplic Cambisol

Date: 26/8/2011

Authors: Sander Tielens & Hagos Mohammedseid

Location: Atsbi

X 0572793 - Y 1527017 –

Z 2558m


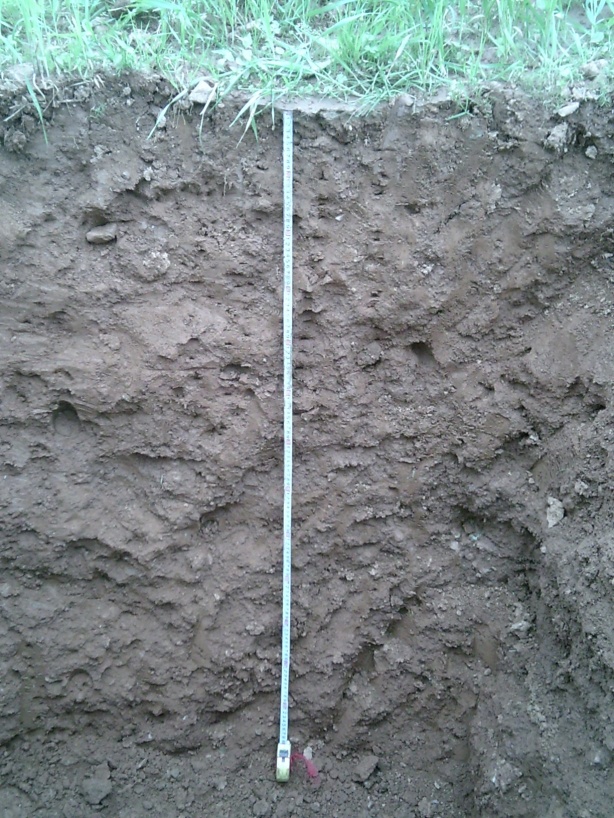


Land use: Cropland (barley)

Parent material: Metavolcanic

Slope: 2%

Surface stoniness: 28%

Relief: Toe slope


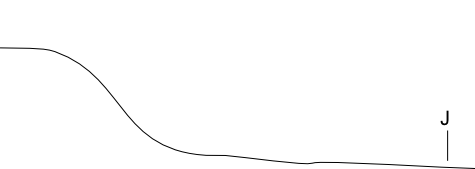


**Profile description**

**Ap** 0-30 cm

Strong brown 7.5 YR 4/6; clay loam; hard when dry; moderate very fine angular; friable when moist; very sticky and plastic when wet; slight reaction with HCl; common roots.

*Gradual smooth boundary to*

**B** 30-130cm

Brown 7.5 YR 4/4; clay loam (decantation: silt loam); very hard when dry; strong fine angular; slightly nutty structure; firm when moist; very sticky and plastic when wet; slight reaction with HCl; few roots.

*Gradual smooth boundary to*

**C**

Reddish brown 5YR 4/4; clay loam; hard when dry; strong fine angular; firm when moist; very sticky and plastic when wet; very slight reaction with HCl no roots; common very small stones.

| **Horizon** | **Depth (cm)** | **FC (%)** | **PWP (%)** | **TAW (%)** | **Bulk density** | **Porosity (%)** |
| --- | --- | --- | --- | --- | --- | --- |
| Ap | 0-30 | 33.17 | 17.43 | 15.74 | 1.43 | 45.06 |
| B | 30-130 | 29.78 | 16.87 | 12.91 | 1.45 | 41.11 |
| C | >130 |  |  |  |  |  |

| **Horizon** | **pH H_2_O** | **pH KCl** | **Org. C (%)** | **Tot. N (%)** | **C/N** | **CaCO3 (%)** | **Pav (mg / kg soil)** | **EC (µS/cm)** |
| --- | --- | --- | --- | --- | --- | --- | --- | --- |
| Ap | 6.68 | 5.31 | 0.63 | 0.07 | 9.58 | 1.75 | 24.91 | 47.80 |
| B | 6.46 | 5.25 | 0.50 | 0.05 | 10.90 | 1.65 |  | 46.20 |
| C | 6.31 | 5.08 | 0.41 | 0.05 | 8.74 | 1.44 |  | 58.80 |

| **Horizon** | **Exch. Ca 2+ *** | **Exch. Mg 2+*** | **Exch. K+*** | **Exch. Na+*** | **ECEC*** | **BS (%)** |
| --- | --- | --- | --- | --- | --- | --- |
| Ap | 11.38 | 4.82 | 0.24 | 0.04 | 19.63 | 84.00 |
| B | 9.40 | 7.91 | 0.13 | 0.15 | 17.60 | 100.00 |
| C | 8.90 | 4.48 | 0.23 | 0.11 | 16.35 | 83.93 |
| *(cmolc/kg) | | | | |  |  |

**Profile K**


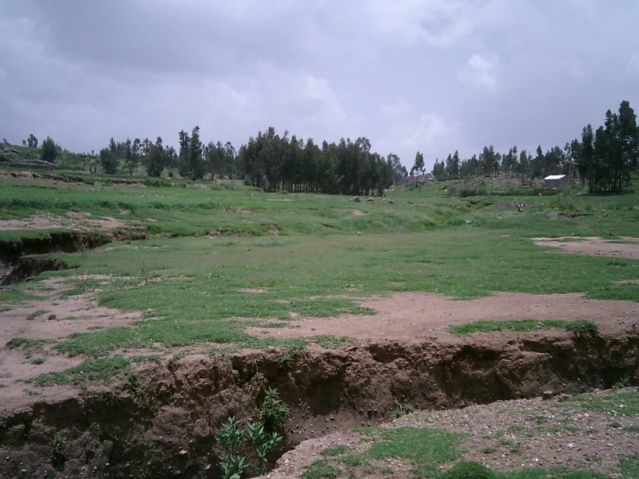


Class: Haplic Fluvisol

Date: 26/8/2011

Authors: Sander Tielens & Hagos Mohammedseid

Location: Atsbi

X 0573333 - Y 1526813 –

Z 2558m


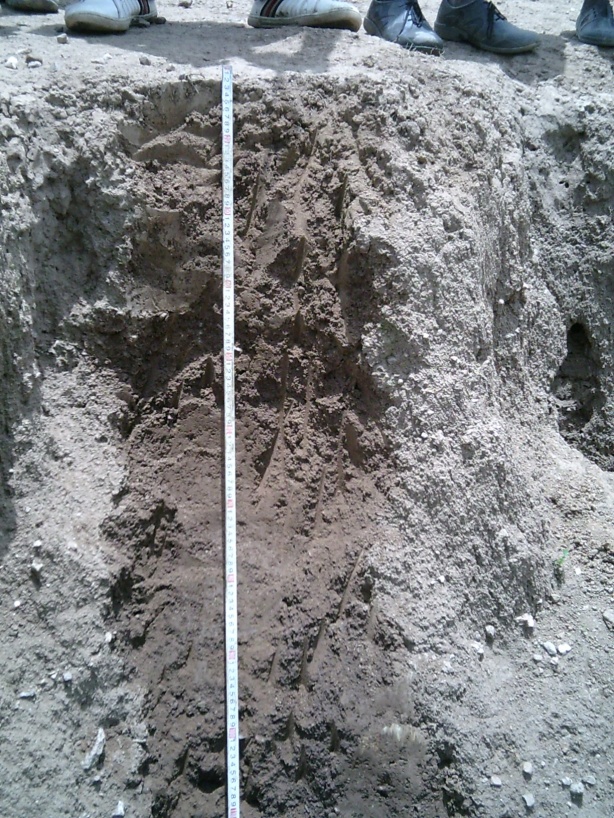


Land use: Grassland

Parent material: Fluvial material

Slope: 2%

Surface stoniness: 8%

Relief: Valley bottom


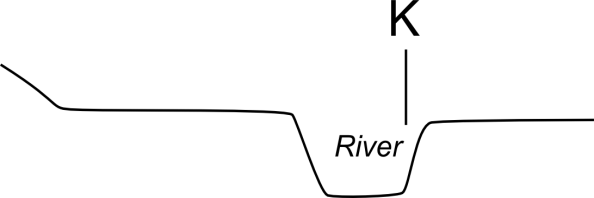


**Profile description**

**C1** 0-35 cm

Brown 7.5 YR 4/4; silt loam; hard when dry; moderate very fine angular; firm when moist; slightly sticky and plastic when wet; slight reaction with HCl; no roots.

*Clear sharp boundary to*

**C2** 35-120 cm

Very dark greyish brown 10 YR 3/2; loam; hard when dry; strong fine angular; friable when moist; sticky and plastic when wet; very slight reaction with HCl; no roots.

| **Horizon** | **Depth (cm)** | **FC (%)** | **PWP (%)** | **TAW (%)** | **Bulk density** | **Porosity (%)** |
| --- | --- | --- | --- | --- | --- | --- |
| C1 | 0-35 | 27.17 | 17.17 | 10.00 | 1.06 | 47.70 |
| C2 | 35-120 | 28.65 | 17.32 | 11.33 | 1.21 | 47.87 |

| **Horizon** | **pH H_2_O** | **pH KCl** | **Org. C (%)** | **Tot. N (%)** | **C/N** | **CaCO3 (%)** | **Pav (mg / kg soil)** | **EC (µS/cm)** |
| --- | --- | --- | --- | --- | --- | --- | --- | --- |
| C1 | 5.77 | 5.26 | 0.75 | 0.11 | 6.65 | 1.17 | 81.11 | 643.00 |
| C2 | 6.07 | 5.05 | 1.20 | 0.11 | 11.27 | 1.72 |  | 71.00 |

| **Horizon** | **Exch. Ca 2+ *** | **Exch. Mg 2+*** | **Exch. K+*** | **Exch. Na+*** | **ECEC*** | **BS (%)** |
| --- | --- | --- | --- | --- | --- | --- |
| C1 | 11.02 | 4.60 | 0.14 | 0.43 | 16.59 | 97.56 |
| C2 | 13.82 | 5.38 | 0.19 | 0.23 | 20.03 | 97.89 |
| *(cmolc/kg) |  |  |  |  |  |  |

**Profile L**


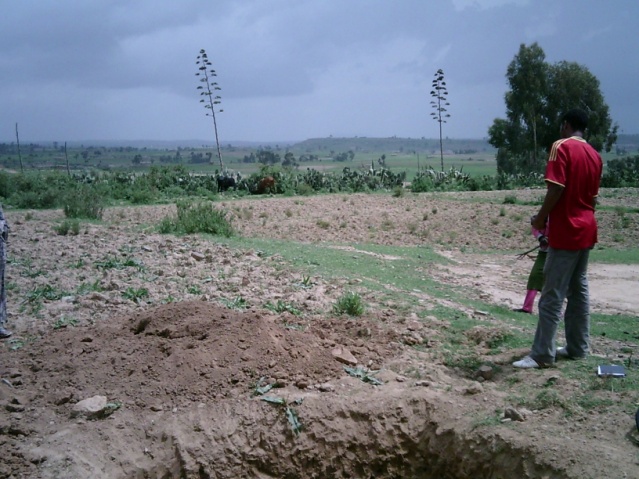


Class: Arenic Lixisol

Date: 26/8/2011

Authors: Sander Tielens & Hagos Mohammedseid

Location: Sinkata

X 0561612 - Y 1553422 –

Z 2400m


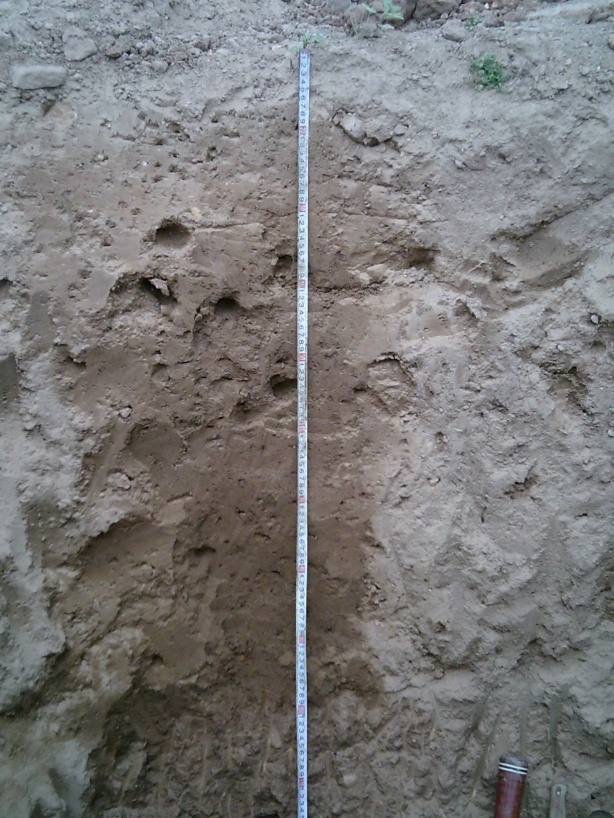


Land use: Cropland

Parent material: Enticho sandstone

Slope: 0%

Surface stoniness: 20%

Relief: Crest, plateau


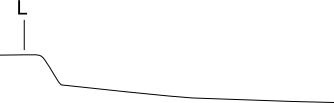


**Profile description**

**Ap** 0-30 cm

Yellowish brown 10 YR 5/6; loamy sand; very friable when dry; moderate fine granular; very friable when moist; very slightly sticky when wet; no reaction with HCl; few roots; very few small stones and common very small stones; Presence of small reddish iron nodules.

*Gradual smooth boundary to*

**B** 30-90 cm

Dark yellowish brown 10 YR 3/4; silt loam; very friable when dry; moderate fine granular; very friable when moist; very slightly sticky and plastic when moist; no reaction with HCl; very few roots; common small stones; Presence of small reddish iron nodules.

| **Horizon** | **Depth (cm)** | **FC (%)** | **PWP (%)** | **TAW (%)** | **Bulk density** | **Porosity (%)** |
| --- | --- | --- | --- | --- | --- | --- |
| Ap | 0-30 | 13.15 | 6.79 | 6.36 | 1.47 | 36.05 |
| B | 30-90 | 12.72 | 7.22 | 5.50 | 1.54 | 33.38 |

| **Horizon** | **pH H_2_O** | **pH KCl** | **Org. C (%)** | **Tot. N (%)** | **C/N** | **CaCO3 (%)** | **Pav (mg / kg soil)** | **EC (µS/cm)** |
| --- | --- | --- | --- | --- | --- | --- | --- | --- |
| Ap | 6.67 | 6.08 | 0.62 | 0.06 | 10.55 | 0.43 | 38.25 | 44.20 |
| B | 6.96 | 6.64 | 0.23 | 0.02 | 9.77 | 0.63 |  | 52.10 |

| **Horizon** | **Exch. Ca 2+ *** | **Exch. Mg 2+*** | **Exch. K+*** | **Exch. Na+*** | **ECEC*** | **BS (%)** |
| --- | --- | --- | --- | --- | --- | --- |
| Ap | -0.18 | 3.07 | 0.30 | 0.02 | 3.21 | 100.00 |
| B | 1.83 | 0.66 | 0.34 | 0.09 | 5.47 | 53.49 |
| *(cmolc/kg) |  |  |  |  |  |  |

**Profile M**


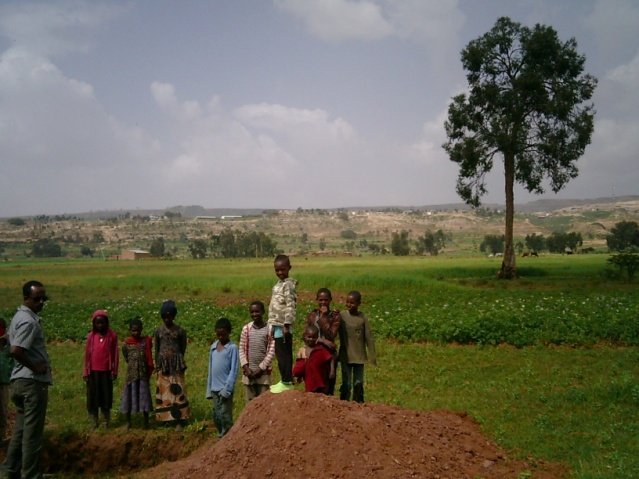


Class: Haplic Fluvisol

Date: 26/8/2011

Authors: Sander Tielens & Hagos Mohammedseid

Location: Sinkata

X 0560532 - Y 1552607 –

Z 2376m


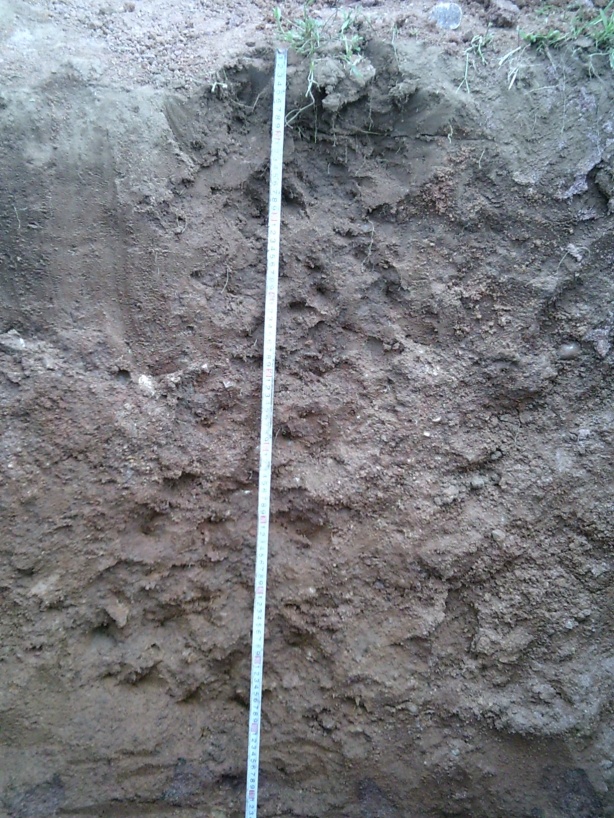


Land use: Cropland

Parent material: Fluvial material

Slope: 0%

Surface stoniness: 2%

Relief: Valley bottom


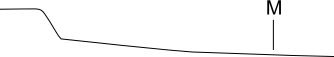


**Profile description**

**Ah** 0-10 cm

Dark brown 7.5 YR 3/4; silt loam; friable when dry; weak fine angular; very friable when moist; very slightly sticky and plastic when wet; very slight reaction with HCl; common roots.

*Smooth gradual boundary to*

**C1** 10-40 cm

Reddish brown 5YR 4/4; silt loam; firm when dry; friable when moist, very slightly sticky and plastic when moist; few roots; slight reaction with HCl; many very small stones.

*Clear sharp boundary to*

**C2** 40-90 cm

Yellowish red 5 YR 4/6; silt loam; hard when dry; firm when moist; very slightly sticky and plastic when wet; no reaction with HCl; few roots; many very small stones and common small stones and even few stones till 5 cm.

| **Horizon** | **Depth (cm)** | **FC (%)** | **PWP (%)** | **TAW (%)** | **Bulk density** | **Porosity (%)** |
| --- | --- | --- | --- | --- | --- | --- |
| Ah | 0-10 | 16.60 | 10.41 | 6.19 | 1.69 | 33.33 |
| C1 | 10-40 |  |  |  |  |  |
| C2 | 40-90 |  |  |  |  |  |

| **Horizon** | **pH H_2_O** | **pH KCl** | **Org. C (%)** | **Tot. N (%)** | **C/N** | **CaCO3 (%)** | **Pav (mg / kg soil)** | **EC (µS/cm)** |
| --- | --- | --- | --- | --- | --- | --- | --- | --- |
| Ah | 6.70 | 5.82 | 0.50 | 0.05 | 9.67 | 0.80 | 58.85 | 56.40 |
| C1 | 6.40 | 5.31 | 0.61 | 0.05 | 12.66 | 0.86 |  | 42.90 |
| C2 | 6.39 | 5.51 | 0.27 | 0.03 | 8.71 | 0.88 |  | 48.40 |

| **Horizon** | **Exch. Ca 2+ *** | **Exch. Mg 2+*** | **Exch. K+*** | **Exch. Na+*** | **ECEC*** | **BS (%)** |
| --- | --- | --- | --- | --- | --- | --- |
| Ah | 6.39 | 3.12 | 0.87 | 0.12 | 11.93 | 87.97 |
| C1 | 13.52 | 6.14 | 0.81 | 0.11 | 21.07 | 97.71 |
| C2 | 13.80 | 6.15 | 0.52 | 0.05 | 21.32 | 96.24 |
| *(cmolc/kg) |  |  |  |  |  |  |

**Profile N**


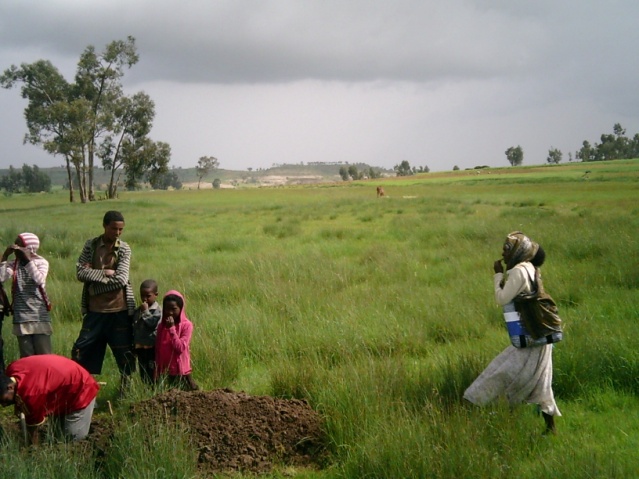


Class: Mazic Sodic Vertisol

Date: 26/8/2011

Authors: Sander Tielens & Hagos Mohammedseid

Location: Sinkata

X 0560474 - Y 1552540 –

Z 2373m


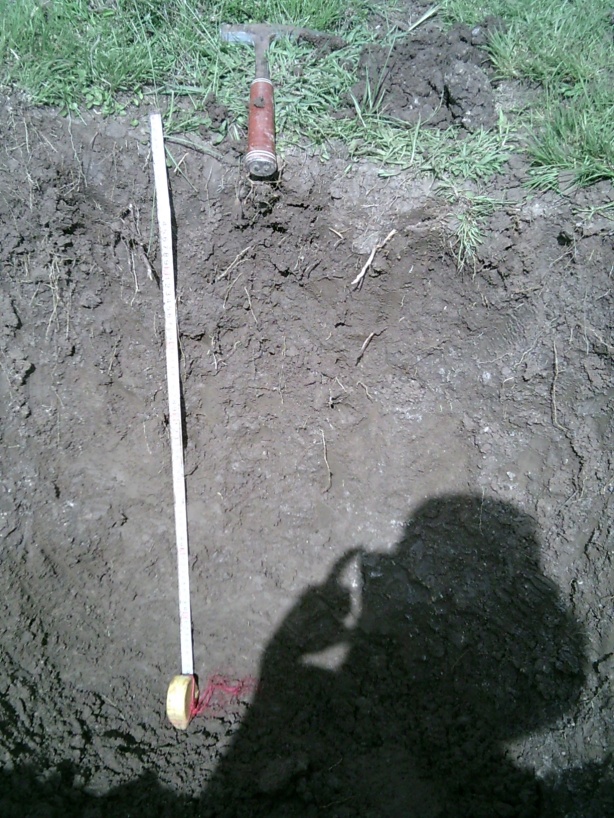
Land use: Grassland

Parent material: Fluvial material

Slope: 0%

Surface stoniness: 0%

Relief: Valley bottom


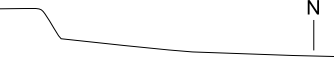


**Profile description**

**Ah** 0-15 cm

Black 10 YR 2/1; clay loam; very hard when dry; moderate very fine angular; firm when moist; sticky and plastic when wet; moderate reaction with HCl; many roots.

*Gradual smooth boundary to*

**B** 15-80 cm

Very dark gray 10 YR 3/1; light clay (decantation: clay loam); very hard when dry; moderate fine angular; firm when moist; sticky and plastic when wet; not fully developed slickensides visible; moderate reaction with HCl; common roots; few very small stones.

| **Horizon** | **Depth (cm)** | **pH H_2_O** | **pH KCl** | **Org. C (%)** | **Tot. N (%)** | **C/N** | **CaCO3 (%)** | **Pav (mg / kg soil)** | **EC (µS/cm)** |
| --- | --- | --- | --- | --- | --- | --- | --- | --- | --- |
| Ah | 0-15 | 7.49 | 6.79 | 0.98 | 0.11 | 9.23 | 1.94 | 11.95 | 461.00 |
| B | 15-80 | 7.81 | 7.38 | 1.45 | 0.09 | 16.07 | 2.54 |  | 1401.00 |

| **Horizon** | **Exch. Ca 2+ *** | **Exch. Mg 2+*** | **Exch. K+*** | **Exch. Na+*** | **ECEC*** | **BS (%)** |
| --- | --- | --- | --- | --- | --- | --- |
| Ah | 10.40 | 12.62 | 0.47 | 2.29 | 28.16 | 91.54 |
| B | 9.08 | 9.87 | 0.40 | 14.24 | 33.59 | 100.00 |
| *(cmolc/kg) | | | | |  |  |

**Profile O**


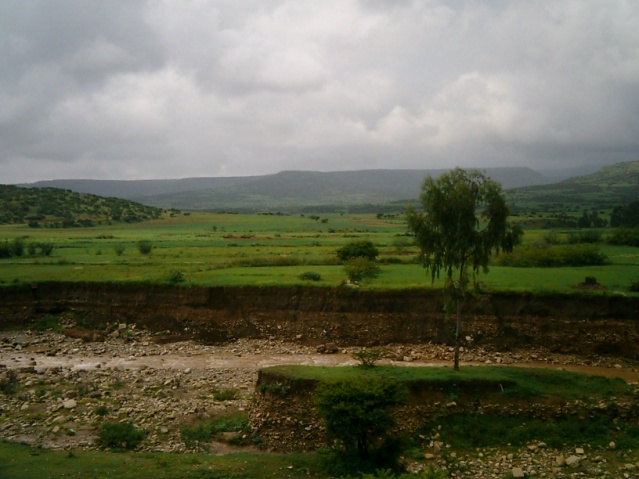


Class: Mollic Calcaric Fluvisol

Date: 2/9/2011

Authors: Sander Tielens & Hagos Mohammedseid

Location: Agula’e

X 0566544 - Y 1514049 –

Z 2131m


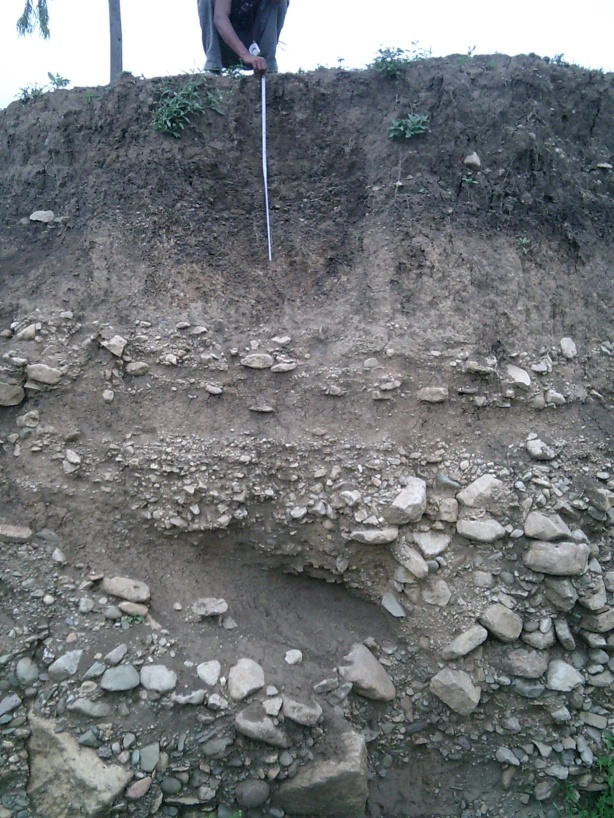


Land use: Cropland

Parent material: Fluvial material

Slope: 0%

Surface stoniness: 2%

Relief: Valley bottom


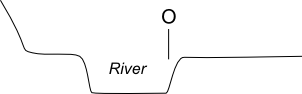


**Profile description**

**C1** 0-40 cm

Dark grey 7.5 YR 4/1; silt loam; hard when dry; moderate fine granular; firm when moist; sticky and plastic when wet; very strong reaction with HCl; many thick roots; few small stones.

*Clear sharp boundary to*

**C2** 40-80 cm

Very dark grey 7.5 YR 3/1; clay loam; firm when dry; moderate fine granular; very firm when moist; very sticky and plastic when wet; many thick roots; very strong reaction with HCl; many small stones; small shells.

*Clear sharp boundary to*

**C3** 80-130 cm

Brown 7.5 YR 5/4; loam; very hard when dry; strong medium granular; firm when moist; slightly sticky and plastic when wet; very strong reaction with HCl; few roots; black mottles.

| **Horizon** | **Depth (cm)** | **FC (%)** | **PWP (%)** | **TAW (%)** | **Bulk density** | **Porosity (%)** |
| --- | --- | --- | --- | --- | --- | --- |
| C1 | 0-40 | 26.37 | 23.32 | 3.05 | 1.22 | 52.66 |
| C2 | 40-80 | 30.78 | 28.17 | 2.61 | 1.47 | 39.70 |
| C3 | 80-130 |  |  |  |  |  |

| **Horizon** | **pH H_2_O** | **pH KCl** | **Org. C (%)** | **Tot. N (%)** | **C/N** | **CaCO3 (%)** | **Pav (mg / kg soil)** | **EC (µS/cm)** |
| --- | --- | --- | --- | --- | --- | --- | --- | --- |
| C1 | 7.75 | 7.19 | 1.32 | 0.09 | 13.97 | 13.93 | 19.07 | 145.70 |
| C2 | 8.03 | 7.24 | 0.96 | 0.09 | 11.26 | 12.01 |  | 137.80 |
| C3 | 8.07 | 7.31 | 0.39 | 0.07 | 5.32 | 19.12 |  | 186.30 |

| **Horizon** | **Exch. Ca 2+ *** | **Exch. Mg 2+*** | **Exch. K+*** | **Exch. Na+*** | **ECEC*** | **BS (%)** |
| --- | --- | --- | --- | --- | --- | --- |
| C1 | 28.29 | 1.70 | 0.58 | 0.16 | 30.73 | 100.00 |
| C2 | 26.36 | 4.65 | 0.69 | 0.35 | 32.05 | 100.00 |
| C3 | 22.51 | 3.50 | 0.52 | 0.37 | 26.90 | 100.00 |
| *(cmolc/kg) |  |  |  |  |  |  |

**Profile P**


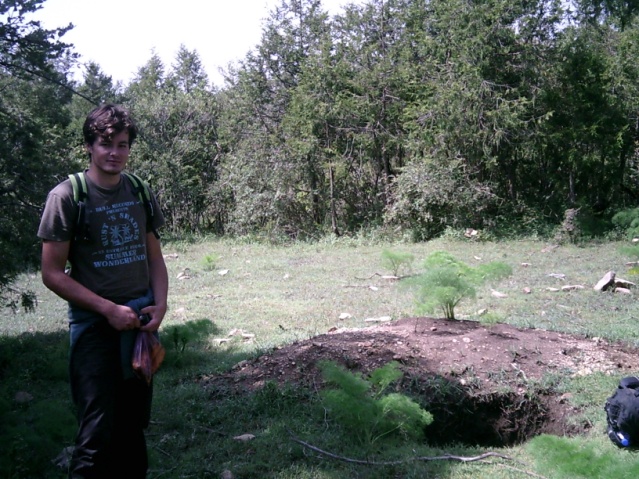


Class: Vertic Calcaric Phaeozem

Date: 6/9/2011

Authors: Sander Tielens & Hagos Mohammedseid

Location: Des’a forest

X 0584612 - Y 1513115 –

Z 2500m


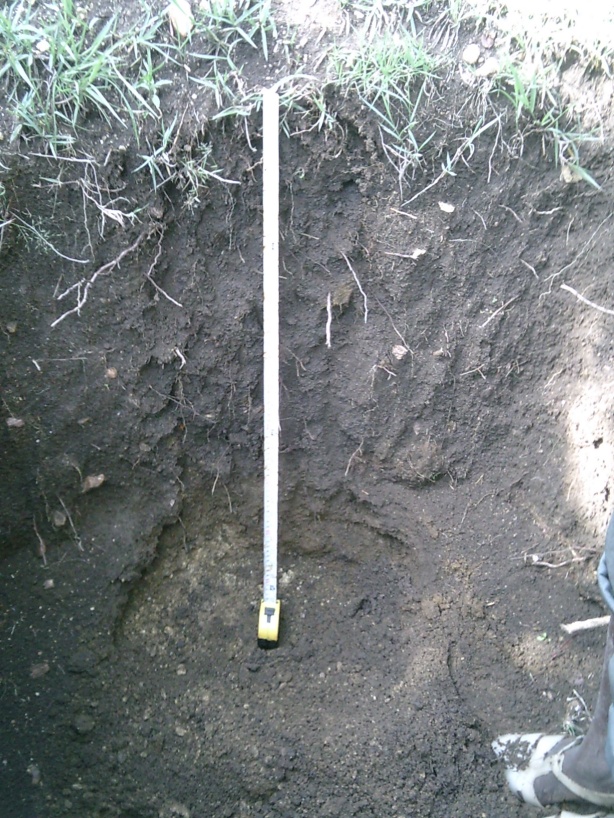
Land use: Forest

Parent material: Antalo limestone

Slope: 5%

Surface stoniness: 0%

Relief: /

**Profile description**

**Ah** 0-15 cm

Very dark grey 5 YR 3/1; silt loam; hard when dry; strong fine crumby; when dry; firm when moist; not sticky and plastic when wet; strong reaction with HCl; many thick and fine roots; very porous.

*Gradual smooth boundary to*

**B** 15-55 cm

Very dark grey 5YR 3/1; silt loam; firm when dry; strong fine crumby; firm when moist; sticky and plastic when wet; very strong reaction with HCl; many thick and fine roots; very porous.

*Clear sharp boundary to*

**B/C** 55-70 cm

Dark brown 7.5 YR 3/3; silt loam; firm when dry; moderate fine crumby; firm when moist; slightly sticky and plastic when wet; very strong reaction with HCl; very few roots.

| **Horizon** | **Depth (cm)** | **FC (%)** | **PWP (%)** | **TAW (%)** | **Bulk density** | **Porosity (%)** |
| --- | --- | --- | --- | --- | --- | --- |
| Ah | 0-15 | 32.77 | 27.24 | 5.53 | 0.91 | 50.85 |
| B | 15-55 | 33.38 | 32.48 | 0.90 | 1.21 | 38.36 |

| **Horizon** | **pH H_2_O** | **pH KCl** | **Org. C (%)** | **Tot. N (%)** | **C/N** | **CaCO3 (%)** | **Pav (mg / kg soil)** | **EC (µS/cm)** |
| --- | --- | --- | --- | --- | --- | --- | --- | --- |
| Ah | 7.65 | 7.09 | 3.96 | 0.36 | 11.08 | 4.36 | 25.68 | 190.60 |
| B | 7.81 | 7.20 | 2.69 | 0.20 | 13.75 | 15.73 |  | 177.30 |

| **Horizon** | **Exch. Ca 2+ *** | **Exch. Mg 2+*** | **Exch. K+*** | **Exch. Na+*** | **ECEC*** | **BS (%)** |
| --- | --- | --- | --- | --- | --- | --- |
| Ah | 33.90 | 1.92 | 0.86 | 0.18 | 36.86 | 100.00 |
| B | 33.10 | 1.20 | 0.75 | 0.10 | 35.15 | 100.00 |
| *(cmolc/kg) |  |  |  |  |  |  |

**Profile Q**


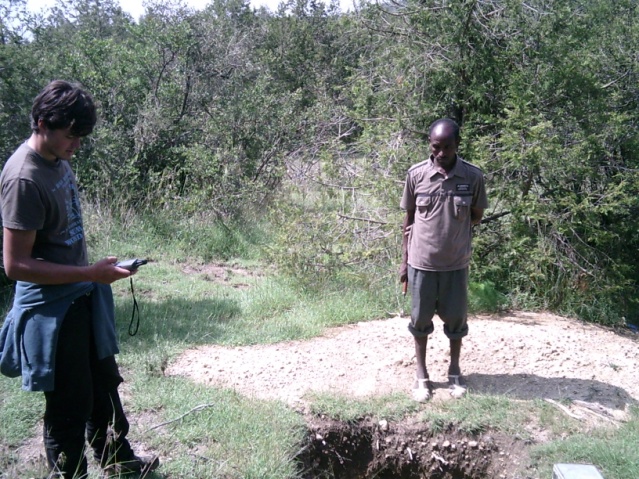


Class: Mollic Calcaric Cambisol

Date: 6/9/2011

Authors: Sander Tielens & Hagos Mohammedseid

Location: Des’a forest

X 0584656 - Y 1513166 –

Z 2509m


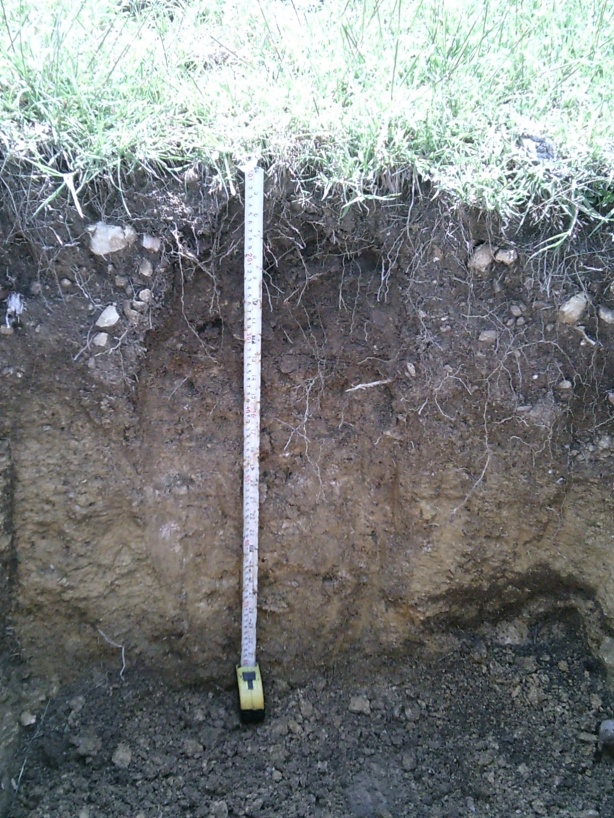
Land use: Forest

Parent material: Antalo limestone

Slope: 4%

Surface stoniness: 0%

Relief: /

**Profile description**

**Ah** 0-20 cm

Brown 10 YR 4/3; silt loam; friable when dry; moderate fine granular; firm when moist; sticky and plastic when wet; very strong reaction with HCl; many fine and medium roots; many medium stones.

*Clear sharp boundary to*

**B** 20-70 cm

Yellow 2.5 Y 7/6; loam (decantation: silt loam); very friable when dry; strong fine crumby; firm when moist; sticky and plastic when wet; very strong reaction with HCl; common fine roots.

| **Horizon** | **Depth (cm)** | **FC (%)** | **PWP (%)** | **TAW (%)** | **Bulk density** | **Porosity (%)** |
| --- | --- | --- | --- | --- | --- | --- |
| Ah | 0-20 | 22.84 | 20.08 | 2.76 | 1.05 | 44.96 |
| B | 20-75 | 30.09 | 15.57 | 14.52 | 1.48 | 42.04 |

| **Horizon** | **pH H_2_O** | **pH KCl** | **Org. C (%)** | **Tot. N (%)** | **C/N** | **CaCO3 (%)** | **Pav (mg / kg soil)** | **EC (µS/cm)** |
| --- | --- | --- | --- | --- | --- | --- | --- | --- |
| Ah | 7.82 | 7.45 | 2.38 | 0.21 | 11.34 | 19.37 | 16.55 | 166.90 |
| B | 8.23 | 7.75 | 3.24 | 0.30 | 10.72 | 18.55 |  | 140.30 |

| **Horizon** | **Exch. Ca 2+ *** | **Exch. Mg 2+*** | **Exch. K+*** | **Exch. Na+*** | **ECEC*** | **BS (%)** |
| --- | --- | --- | --- | --- | --- | --- |
| Ah | 27.77 | 0.49 | 0.66 | 0.11 | 29.04 | 100.00 |
| B | 14.17 | 0.26 | 0.45 | 0.07 | 17.20 | 86.94 |
| *(cmolc/kg) | | | | |  |  |

**Profile R**


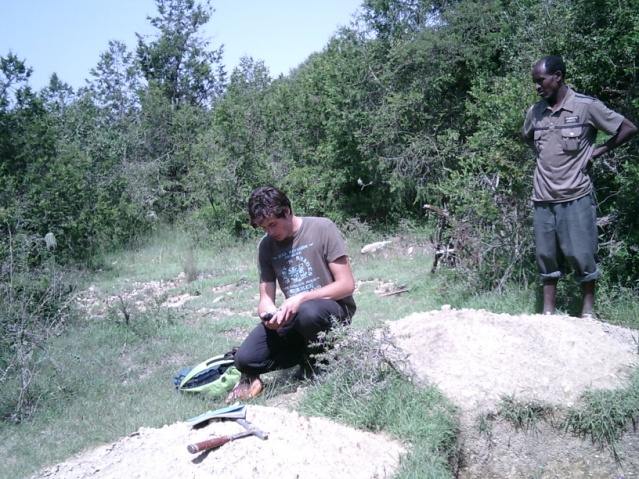


Class: Mollic Calcaric Cambisol

Date: 6/9/2011

Authors: Sander Tielens & Hagos Mohammedseid

Location: Des’a forest

X 0584821 - Y 1512925 –

Z 2489m


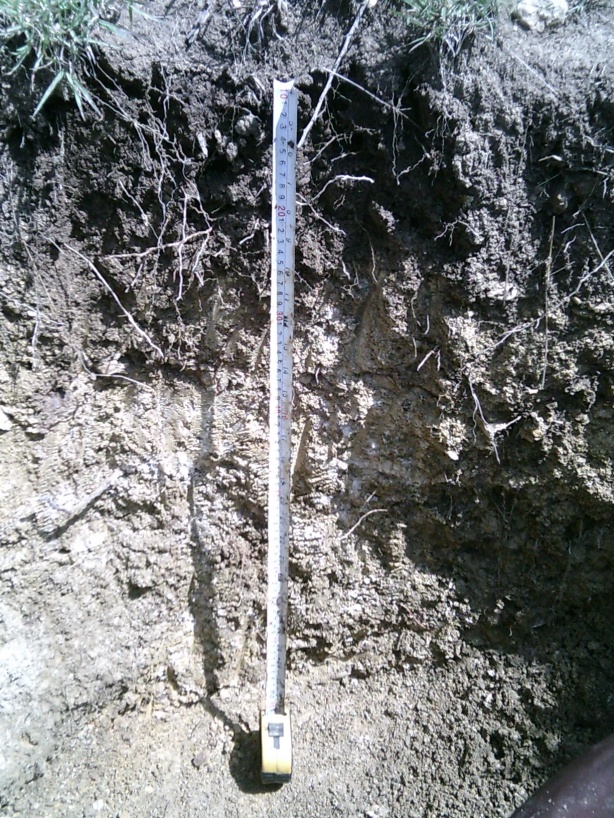


Land use: Forest

Parent material: Antalo limestone

Slope: 7%

Surface stoniness: 0%

Relief: /

**Profile description**

**Ah** 0-15 cm

Dark greyish brown 10 YR 4/2; silt loam; hard when dry; moderate fine granular; firm when moist; sticky and plastic when wet; very strong reaction with HCl; many thick roots; rock fragments till 8 cm.

*Clear sharp boundary to*

**B1** 15-40 cm

Yellowish brown 10 YR 5/4; silt loam; firm when dry; moderate fine granular; friable when moist; slightly sticky and plastic when wet; very strong reaction with HCl; common medium roots; white layers.

*Gradual smooth boundary to*

**B2** 40-80 cm

Yellowish brown 10 YR 5/6; silt loam; firm when dry; moderate fine granular; firm when moist; sticky and plastic when wet; very strong reaction with HCl; very few fine roots.

| **Horizon** | **Depth (cm)** | **FC (%)** | **PWP (%)** | **TAW (%)** | **Bulk density** | **Porosity (%)** |
| --- | --- | --- | --- | --- | --- | --- |
| Ah | 0-15 | 26.13 | 21.62 | 4.51 | 1.20 | 44.25 |
| B1 | 15-40 | 25.52 | 17.03 | 8.49 | 1.46 | 39.09 |
| B2 | 40-80 |  |  |  |  |  |

| **Horizon** | **pH H_2_O** | **pH KCl** | **Org. C (%)** | **Tot. N (%)** | **C/N** | **CaCO3 (%)** | **Pav (mg / kg soil)** | **EC (µS/cm)** |
| --- | --- | --- | --- | --- | --- | --- | --- | --- |
| Ah | 8.05 | 7.56 | 3.67 | 0.19 | 18.91 | 19.23 | 13.92 | 156.10 |
| B1 | 8.16 | 7.54 | 0.81 | 0.09 | 9.03 | 18.76 |  | 128.30 |
| B2 | 8.04 | 7.23 | 0.54 | 0.07 | 8.15 | 19.36 |  | 138.50 |

| **Horizon** | **Exch. Ca 2+ *** | **Exch. Mg 2+*** | **Exch. K+*** | **Exch. Na+*** | **ECEC*** | **BS (%)** |
| --- | --- | --- | --- | --- | --- | --- |
| Ah | 27.94 | 0.96 | 0.81 | 0.13 | 29.84 | 100.00 |
| B1 | 19.98 | 0.60 | 0.71 | 0.09 | 21.77 | 98.23 |
| B2 | 29.93 | 1.76 | 1.48 | 0.12 | 33.30 | 100.00 |
| *(cmolc/kg) |  |  |  |  |  |  |

**Profile S**


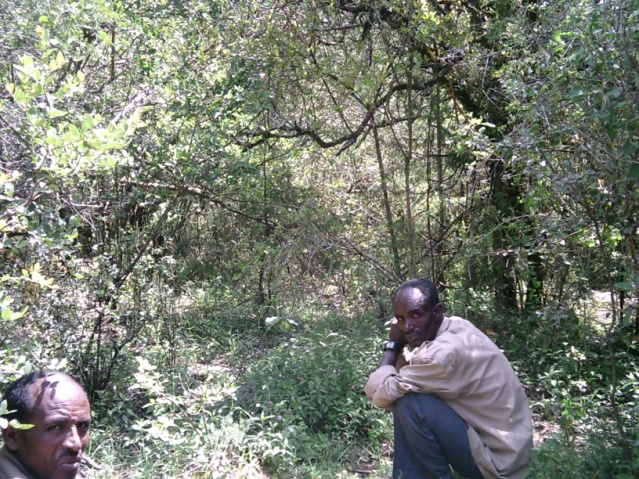


Class: Rendzic Leptosol

Date: 6/9/2011

Authors: Sander Tielens & Hagos Mohammedseid

Location: Des’a forest

X 0584827 - Y 1512858 –

Z 2483m


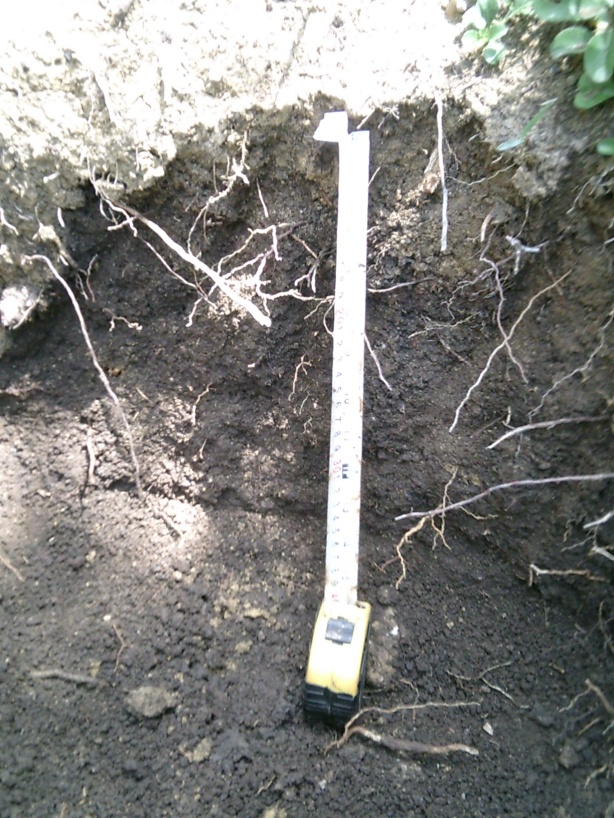
Land use: Forest

Parent material: Antalo limestone

Slope: /

Surface stoniness: 0%

Relief: /

**Profile description**

**Ah** 0-5 cm

Brown 7.5 YR 4/3; loam; friable when dry; strong fine angular; very firm when moist; sticky and plastic when wet; very strong reaction with HCl; many thick and fine roots.

*Clear sharp boundary to*

**B** 5-25 cm

Dark brown 7.5 YR 3/3; silt loam; firm when dry; strong fine granular; firm when moist; slightly sticky and plastic; strong reaction with HCl; many thick roots.

| **Horizon** | **Depth (cm)** | **FC (%)** | **PWP (%)** | **TAW (%)** | **Bulk density** | **Porosity (%)** |
| --- | --- | --- | --- | --- | --- | --- |
| Ah | 0-5 | 32.27 | 21.71 | 10.56 | 1.02 | 49.90 |
| B | 5-25 | 35.36 | 34.64 | 0.72 | 1.17 | 50.44 |

| **Horizon** | **pH H_2_O** | **pH KCl** | **Org. C (%)** | **Tot. N (%)** | **C/N** | **CaCO3 (%)** | **Pav (mg / kg soil)** | **EC (µS/cm)** |
| --- | --- | --- | --- | --- | --- | --- | --- | --- |
| Ah | 7.83 | 7.19 | 2.43 | 0.16 | 14.81 | 19.28 | 19.11 | 171.10 |
| B | 7.58 | 6.91 | 4.07 | 0.30 | 13.48 | 4.45 |  | 216.00 |

| **Horizon** | **Exch. Ca 2+ *** | **Exch. Mg 2+*** | **Exch. K+*** | **Exch. Na+*** | **ECEC*** | **BS (%)** |
| --- | --- | --- | --- | --- | --- | --- |
| Ah | 28.94 | 3.62 | 0.98 | 0.08 | 33.62 | 100.00 |
| B | 33.38 | 2.49 | 0.67 | 0.22 | 36.76 | 100.00 |
| *(cmolc/kg) |  |  |  |  |  |  |

**Profile T**


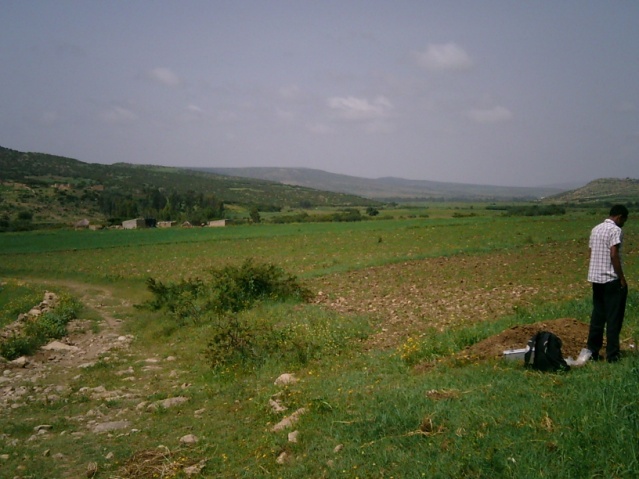


Class: Mollic Calcaric Cambisol

Date: 7/9/2011

Authors: Sander Tielens & Hagos Mohammedseid

Location: Birki

X 0569049 - Y 1515326 –

Z 2069m


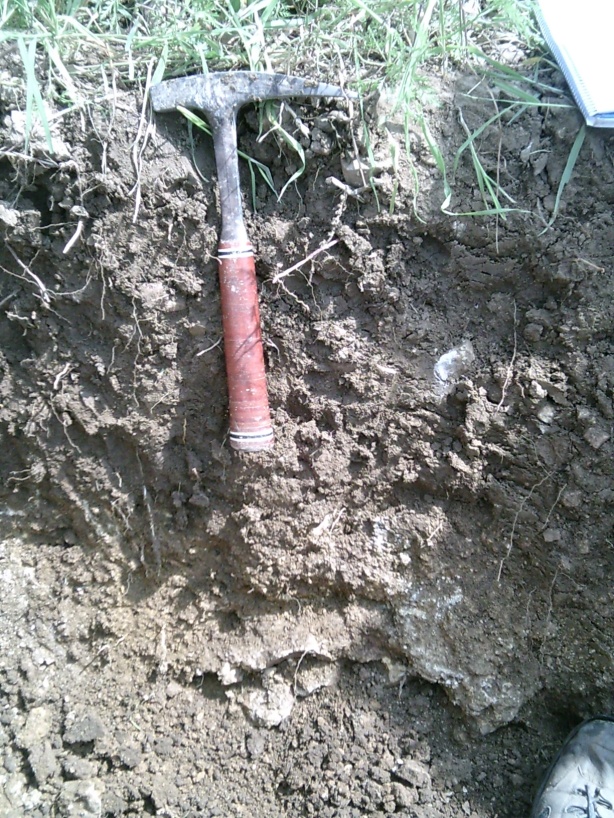


Land use: Cropland

Parent material: Antalo limestone

Slope: 2%

Surface stoniness: 20%

Relief: Plateau


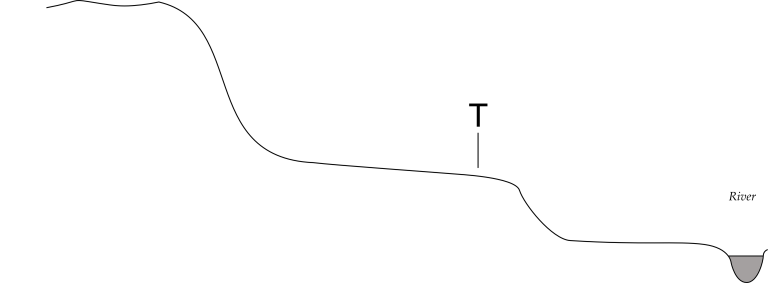


**Profile description**

**Ap** 0-25 cm

Dark yellowish brown 10 YR 4/6; silt loam; hard when dry; weak fine granular; firm when moist; slightly sticky and plastic when wet; very strong reaction with HCl; many thick and fine roots; common rock fragments (4-8 cm).

*Gradual smooth boundary to*

**B** 25-40 cm

Very dark grey 7.5 YR 3/1; silt loam; firm when dry; moderate fine granular; friable when moist; not sticky and plastic when wet; very strong reaction with HCl; common fine roots.

| **Horizon** | **Depth (cm)** | **FC (%)** | **PWP (%)** | **TAW (%)** | **Bulk density** | **Porosity (%)** |
| --- | --- | --- | --- | --- | --- | --- |
| Ap | 0-25 | 30.06 | 25.94 | 4.12 | 1.33 | 40.28 |
| B | 25-40 | 30.80 | 27.81 | 2.99 | 1.44 | 40.98 |

| **Horizon** | **pH H_2_O** | **pH KCl** | **Org. C (%)** | **Tot. N (%)** | **C/N** | **CaCO3 (%)** | **Pav (mg / kg soil)** | **EC (µS/cm)** |
| --- | --- | --- | --- | --- | --- | --- | --- | --- |
| Ap | 7.72 | 7.19 | 1.86 | 0.19 | 9.67 | 19.06 | 42.77 | 161.60 |
| B | 7.82 | 7.31 | 1.75 | 0.18 | 9.97 | 19.09 |  | 148.10 |

| **Horizon** | **Exch. Ca 2+ *** | **Exch. Mg 2+*** | **Exch. K+*** | **Exch. Na+*** | **ECEC*** | **BS (%)** |
| --- | --- | --- | --- | --- | --- | --- |
| Ap | 23.94 | 1.91 | 3.69 | 0.07 | 29.60 | 100.00 |
| B | 25.43 | 1.43 | 1.81 | 0.20 | 29.43 | 98.10 |
| *(cmolc/kg) |  |  |  |  |  |  |
